# Phaeoviruses Present in Cultured and Natural Kelp Species, *Saccharina latissima* and *Laminaria hyperborea* (Phaeophyceae, Laminariales)*,* in Norway

**DOI:** 10.3390/v15122331

**Published:** 2023-11-28

**Authors:** Eliana Ruiz Martínez, Dean A. Mckeown, Declan C. Schroeder, Gunnar Thuestad, Kjersti Sjøtun, Ruth-Anne Sandaa, Aud Larsen, Ingunn Alne Hoell

**Affiliations:** 1 Department of Safety, Chemistry and Biomedical Laboratory Sciences, Western Norway University of Applied Sciences, 5528 Haugesund, Norway; gunnar.thuestad@hvl.no (G.T.); ingunn.hoell@hvl.no (I.A.H.); 2 Department of Veterinary Population Medicine, College of Veterinary Medicine, University of Minnesota, Minneapolis, MN 55108, USA; dmckeow@umn.edu (D.A.M.); dcschroe@umn.edu (D.C.S.); 3 Department of Biological Sciences, University of Bergen, 5006 Bergen, Norway; kjersti.sjotun@uib.no (K.S.); ruth.sandaa@uib.no (R.-A.S.); 4 NORCE Norwegian Research Centre, 5008 Bergen, Norway; aula@norceresearch.no

**Keywords:** phaeovirus, *Phycodnaviridae*, Nucleocytoviricota, kelp, prevalence, phylogeny, MCP

## Abstract

Phaeoviruses (*Phycodnaviridae*) are large icosahedral viruses in the phylum Nucleocytoviricota with dsDNA genomes ranging from 160 to 560 kb, infecting multicellular brown algae (Phaeophyceae). The phaeoviral host range is broader than expected, not only infecting algae from the Ectocarpales but also from the Laminariales order. However, despite phaeoviral infections being reported globally, Norwegian kelp species have not been screened. A molecular analysis of cultured and wild samples of two economically important kelp species in Norway (*Saccharina latissima* and *Laminaria hyperborea*) revealed that phaeoviruses are recurrently present along the Norwegian coast. We found the viral prevalence in *S. latissima* to be significantly higher at the present time compared to four years ago. We also observed regional differences within older samples, in which infections were significantly lower in northern areas than in the south or the fjords. Moreover, up to three different viral sequences were found in the same algal individual, one of which does not belong to the *Phaeovirus* genus and has never been reported before. This master variant therefore represents a putative new member of an unclassified phycodnavirus genus.

## 1. Introduction

The genus phaeovirus belongs to the *Phycodnaviridae* family within the giant virus phylum Nucleocytoviricota [1] and comprises large icosahedral viruses with dsDNA genomes ranging from 160 to 560 kb [2]. Phycodnaviruses infect numerous taxa of algae [3], but only a few seaweed viruses have been fully characterized. Phaeoviruses are the only giant viruses known to infect multicellular algae [4,5,6], more specifically, seven species grouped within four different families within Ectocarpales and eight species belonging to three different families of the order Laminariales, commonly known as kelp [7,8]. Ectocarpoid phaeoviruses are divided into two subgroups based on the concatenated phylogeny of DNA polymerase and major capsid protein (MCP): subgroup A, consisting of one virus genotype that infects *Ectocarpus*, *Pylaiella*, *Myriotrichia*, *Hincksia*, *Ecklonia*, and *Undaria*, and subgroup B, which consists of multiple viral genotypes that only infect *Feldmannia* [8]. Based solely on MCP phylogeny, kelp phaeovirus subgroup C has recently been found in *Laminaria digitata* (Hudson) J.V. Lamoroux, *Laminaria hyperborea* (Gunnerus) Foslie, and *Saccharina latissima* (Linnaeus) C.E.Lane, C.Mayes, Druehl & G.W.Saunders, and subgroup D is represented by a viral sequence found in *Saccharina japonica* (Areschoug) C.E.Lane, C.Mayes, Druehl & G.W.Saunders [7].

Viral particles infecting multicellular brown algae enter their wall-less life-cycle stages; the motile spores or gametes [4,9,10]. Upon its entry, the genome of the virus can integrate within the host’s genome without killing it and persist as a provirus [11]. After the host zoid has settled into a suited substrate, a copy of the virus will be transmitted through mitosis to all cells of the developing alga [10,12,13]. Adult sporophytes or gametophytes carrying the provirus can appear without any visible signals of survival or growth impairment [14] and with the ability to produce viable spores or gametes that contain the proviral genome [15]. However, deformed reproductive organs can appear after viral induction, severely reducing algae reproduction [16,17]. The persistent life strategy represents a stable evolutionary co-existence of a virus and host that is unique within the nucleocytoviruses [16], where r-selected (phaeoviral subgroups B, C, and D) are more commonly found than K-selected ones (phaeoviral subgroup A) [16,18].

Many Laminariales form extensive underwater kelp forests, dominating shallow subtidal rocky habitats in most temperate coastal areas around the world [19]. These structurally complex and highly productive [20,21] habitats enhance local biodiversity [22,23,24] and support food webs in coastal areas through secondary production [24,25]. Kelp forests are under pressure due to herbivore outbreaks, eutrophication, ocean warming, and/or other climate-induced changes [24,26]. It has been estimated that about 38% of the world’s kelp forests have disappeared over the past five decades [23,27]. A recent example is the 80% reduction in sugar kelp (*S. latissima*) along the south coast of Norway, which has partially been replaced by filamentous turf algae [27]. Nevertheless, it has been shown that some fluctuations might have occurred without an obvious cause [28].

Phaeoviruses are found on all continents except Antarctica [8,12], with a prevalence of 50–100% in samples from Ectocarpales [29,30] and between 20 and 100% in samples of different wild kelp species [8]. The present study focuses on screening *S. latissima* and *L. hyperborea* for phaeoviruses in order to investigate if they are prevalent in kelps along the Norwegian coast. *S. latissima* and *L. hyperborea* are important raw materials for the Norwegian seaweed industry [31,32]. *L. hyperborea* is harvested from wild kelp forests to extract alginate, whereas *S. latissima* is cultivated due to its fast growth rate and high carbohydrate content and is used as feed as well as in bioremediation and biogas research [32]. Ocean warming may already have impacted the distribution of several algal species [33], and future predictions point towards higher pathogen prevalence and virulence [34]. Despite this, we know little about the distribution, determinants, and patterns of European algal pathogens, including viruses [35].

The objective of the present study is to determine the diversity, distribution, and prevalence of phaeoviruses in Norwegian kelps in the context of both coastal ecosystem management and food production in the aquaculture sector. The specific objectives include (1) screening for viral presence in natural and cultured relevant kelp species along the Norwegian coast, (2) describing their spatio-temporal patterns, (3) determining their prevalence and putative host range, and (4) identifying phylogenetic relationships among local and previously characterized kelp viruses to unveil their true diversity at both a regional and global scale.

## 2. Materials and Methods

### 2.1. Sampling

**Figure 1:**
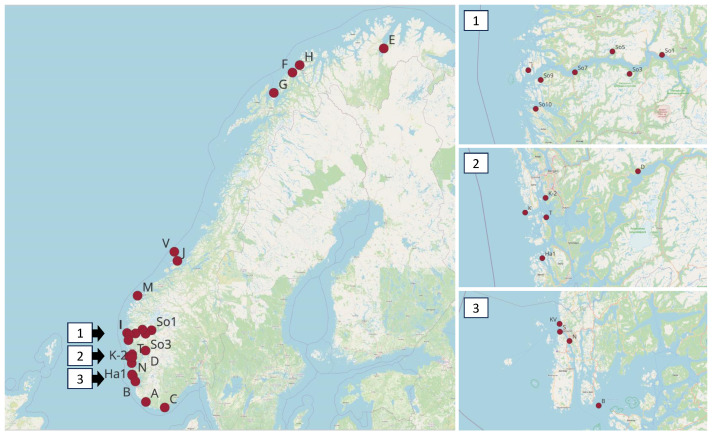
Sampling stations. Full map to the left, and detailed sampling areas to the right. 1—Sognefjorden area, 2—Korsfjorden and Hardangerfjorden areas, and 3—Rogaland area.

Cultured *Saccharina latissima* sporophyte samples were provided by our industry partner, Ocean Forest, and harvested during Spring 2021 in Austevoll (Rogaland, Norway) (Table 1, Figure 1). Field samples of sporophytes of *Laminaria hyperborea* were obtained from our industry partner, IFF N&H Norway AS, and harvested in different locations along the Norwegian coast throughout 2021 (Table 1, Figure 1). Field samples of *S. latissima* were collected by hand around the coastal and harbor areas around Haugesund and Karmøy (Rogaland, Norway). In order to capture possible seasonal changes related to viral presence, we sampled both kelp species from naturally growing populations once per season during 2021 in Korsfjorden (Bergen, Hordaland, Norway) (Table 1, Figure 1). S. *latissima* was sampled with a triangular dredge, and *L. hyperborea* was sampled with a grab dredge.

We also had access to frozen DNA samples extracted from *S. latissima* sporophytes from a previous microsatellite study from a total of 21 stations along the Norwegian coast (Table 1, Figure 1) between August 2016 and June 2018 [36]. The stations ranged from Vest-Agder in south–east Norway to Porsangerfjorden in Northern Norway. Several stations were sampled inside two long and narrow fjords in south–west Norway (Hardangerfjorden and Sognefjorden). The sampling procedure depended on the depth range of S. latissima at the site and local topography [36].

We collected between 10 and 40 samples per kelp species per sampling station and cut epiphyte-free, clean meristematic tissue from all the kelp sporophytes (1–2 cm^2^, approx.) before storing them in 50 mL falcon tubes containing approximately 30 g of silica gel until DNA extraction [7,8].

### 2.2. DNA Extraction

We homogenized 10–20 mg dry weight of sporophyte material using the gentleMACS™ Dissociator (Miltenyi Biotec, Bergisch Gladbach, Germany) before extracting DNA with the NucleoSpin^®^ Plant II kit (Machery-Nagel, Düren, Germany). We followed the standard protocol (using Lysis Buffer PL1) based on the established CTAB procedure [37] with a couple of changes: the lysis step was incubated at room temperature for one hour, and the DNA was eluted into RNase-free water.

DNA samples provided by UiB were homogenized using the Qiagen TissueLyser II (Qiagen, Hilden, Germany) and extracted with either the Qiagen DNeasy^®^ 96 Plant Kit (Qiagen, Hilden, Germany) or the NucleoMag^®^ Plant kit (Machery-Nagel, Düren, Germany) [38]. The extraction was carried out following the protocols, with the following few exceptions: for the Qiagen DNeasy Plant Kit procedure, the centrifugation steps were prolonged, and when applying the NucleoMag Plant Kit, the modifications suggested by Fort and colleagues [39] were followed, meaning that the lysis of samples was performed for 2 h at 56 °C with the addition of 20 μL of 1 mg/mL of proteinase K (Sigma-Aldrich P6556) and 3 μL of RNAse A (provided). After lysis, samples were centrifugated for 15 min at 4 °C (instead of room temperature), and the supernatant was used for the rest of the DNA extraction. 

### 2.3. DNA Amplification by PCR

Diluted (1:10) aliquots of all stock DNA solutions, 799 samples in total (Tables S1 and S2), were used as templates for PCR reactions. We used previously published primers for the phaeovirus major capsid protein (MCP-forward primer: 5′-CVGCGTACTGGGTGAACGC-3′ and MCP-reverse primer: 5′-AGTACTTGTTGAACCAGAACGG-3′) [8,40]. All PCRs were performed using Qiagen’s HotStarTaq Master Mix Kit (Qiagen, Hilden, Germany) according to the manufacturer’s instructions, with the addition of 4 µL of 0.8 mg/mL bovine serum albumin (BSA) per 50 µL reaction. The PCR conditions were as follows: an initial extension of 95 °C for 5 min, then 40 cycles of 95 °C for 1 min, 55 °C for 30 s, and 72 °C for 30 s, and a final extension of 72 °C for 10 min.

### 2.4. Oxford Nanopore Technologies (ONT) Sequencing and Raw Sequence Processing

We selected 187 PCR viral positive products for sequencing using Oxford Nanopore Technologies (ONT) (Table S3).

The library was prepared using the Rapid Barcoding Kit 96 (SQK-RBK 110.96) according to the manufacturer’s instructions (ONT, Oxford, UK). The sequencing was performed by Flow Cell (R9.4.1) (ONT, Oxford, UK) on a GridION Mk1 device (ONT, Oxford, UK) for 24 h, and we used the native ONT software MinKNOW (v21.02.05) (ONT, Oxford, UK). Basecalling was performed in real time by the GridION using the native Guppy basecaller (v4.3.4) (ONT, Oxford, UK).

After the sequencing was complete, the raw sequence reads were processed using a custom pipeline, detailed as follows. First, the ONT sequencing adaptors were trimmed off the read ends using Porechop (v0.2.1) (https://github.com/rrwick/Porechop, accessed on 11 October 2022), followed by a single round of sequence error polishing using Racon (v1.5.0) (https://github.com/isovic/racon, accessed on 11 October 2022). Contaminant sequences (anything not related to the *Phaeovirus* MCP PCR product) were removed by mapping the reads with Minimap2 (v2.2.24) [41] against reference sequences for *Phaeovirus* MCP sequences (NCBI accessions: HG003340.1, HG003341.1, HG003343.1, MG967366.1, MG967367.1, MG967370.1, HG003336.1, HG003333.1, HG003335.1, HG003334.1, HG003337.1, HG003339.1, NC_011183, HG003342.1, HG003338.1, KP296733.1, KP296731.1, MG967376.1, KP296734.1). In addition, low-quality read ends were trimmed using fgbio ClipBam (http://fulcrumgenomics.github.io/fgbio/, accessed on 11 October 2022). Finally, sequences below 180 bp (around 20 bp less than the expected MCP PCR product) were removed, and cd-hit was used to remove redundant sequences (within each kelp sample, any sequences that were 100% identical to another were removed, keeping whichever sequence was longer). The resulting amplicon sequences were used for all downstream analyses.

### 2.5. Downstream Analysis

Reads were aligned with the reference amplicons in Geneious Prime 2023.1.1 (https://www.geneious.com, accessed on 20 February 2023) using MAFFT [42], and the alignment was uploaded to the IQ-TREE server [43] using ModelFinder [44] and UFBoot2 [45] to generate a phylogenetic tree with support values gained from 1000 Ultrafast Bootstrap iterations. In order to generate the phylogenetic tree, translated protein sequences from our amplified kelp MCP gene fragments (Table S3) and known viral MCP genes (mainly *Phycodnaviridae* and *Mimiviridae*) were used (Table S4). MCP from the poxvirus *Fowlpox virus (Poxviridae)* was used to root the tree, and only bootstraps >70 were shown. The tree was posteriously edited with iTOL v6 [46].

Plots and a statistical analysis were made using the tidyverse (v2.0.0) [47], ape (v5.7-1) [48], vegan (v2.6-4) [49], and ggplot2 (v3.4.2) [50] R packages (v 4.2.2) [51] in R Studio (v2023.03.0+386) [52] and Microsoft Excel [53].

NCBI blastn and blastp searches were conducted through Geneious Prime 2023.1.1 (https://www.geneious.com, accessed on 20 February 2023).

## 3. Results

### 3.1. Viral Prevalence on Norwegian Kelp Species

The amplified MCP gene fragments varied between 200 and 300 bp (Figure S1). We detected the phaeoviral MCP gene fragment in 97.4% of our *Laminaria hyperborea* samples, in 94.1% of the *Saccharina latissima* samples from 2021 to 2022, and in 84.3% of *S. latissima* samples from 2016 to 2018 (Table S5). Overall, we found a viral prevalence of 88.7% in our kelp samples (Table S5).

We observed a significantly lower number of infections among the *S. latissima* samples from 2016 to 2018 (*t*-test: two-sample, assuming unequal variances, M = 0.631; SD = 0.234) than in those from 2021 to 2022 (*t*-test: two-sample, assuming unequal variances, M = 0.943, SD = 0.054); t(230) = −8.360, (*p* < 0.001).

There were significant differences in the number of infected and non-infected samples from different areas (South, North, and Fjords; see Table 1) within the *S. latissima* samples from 2016 to 2018 as well (ANOVA: single factor, F(2, 184) = [9.52381042], *p* < 0.001) (Table S6). A post hoc Tukey–Kramer test showed that there were significant differences between the groups South–North and Fjords–North at *p* < 0.05 but not within the South–Fjords samples. Since there appears to be a significant decrease in the infections in the northern areas (Table S6) and during 2021–2022, we did not obtain *S. latissima* samples from the north (or the fjords), and we compared again the number of infections in old vs. new *S. latissima* samples but only from the southern regions. Our results showed these to be still significantly different (*t*-test: two-sample, assuming equal variances, M = 0.722, SD = 0.204) (*t*-test: two-sample, assuming equal variances, M = 0.943, SD = 0.054), respectively; *t*(448) = 6.345, (*p* < 0.001).

### 3.2. Sequencing and Viral Variants

Sequencing our PCR products generated a total number of 1,724,818 reads, with an average number of 7113 reads per sample after processing (Table S3). Each sample was mapped against a set of reference sequences, and up to three different possible viral master variants (named master variant I, II, and III) were found within the same sample (Figure S2). The raw sequence reads and processed amplicon sequences were submitted to GenBank under BioSample accessions from SAMN37905911 to SAMN37906097. A blastn search was performed, and all three retrieved the highest homologies with the EsV-1 MCP gene (Figure S3).

**Figure 2:**
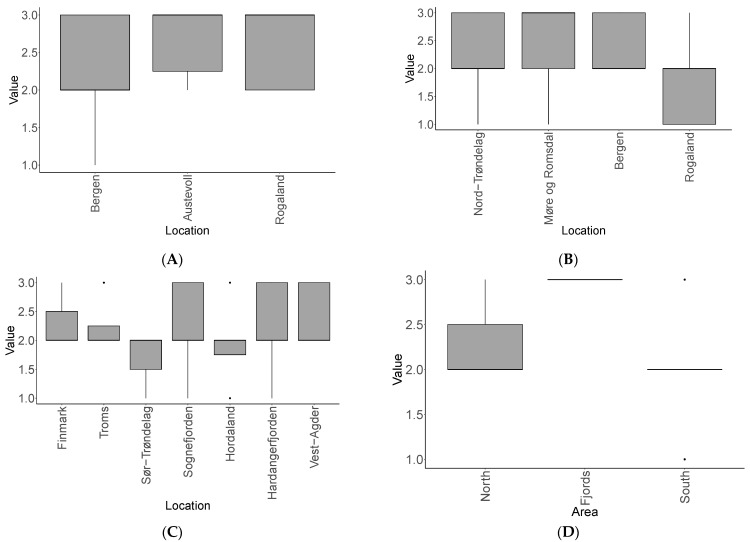
Number of possible viral master variants per kelp sample and per location (**A**–**C**) or per area (**D**) on average. (**A**) *L. hyperborea* 2021–2022; (**B**) *S. latissima* 2021–2022; (**C**,**D**) *S. latissima* 2016–2018 (see Table S3).

The number of master variants per sample was significantly different depending on the location for *L. hyperborea* (ANOVA: single factor, F(3, 63) = [2.86644665], *p* = 0.04), with two or three master variants per sample on average: K (Bergen) (2), V (Nord-Trøndelag) (2), B (Rogaland) (2), and M (Møre og Romsdal) (3) (Figure 2A). A post hoc Tukey–Kramer test showed that these differences were only between the groups Bergen–Rogaland and Møre og Romsdal–Rogaland at *p* < 0.05.

We did not find any significant differences between the number of master variants per sample and their locations for *S. latissima* for 2021–2022 (*p* = 0.437), nor for *S. latissima* for 2016–2018 (*p* = 0.549). An average number of three master variants per *S. latissima* sample from Austevoll and Rogaland in 2021–2022 and two in those from Bergen (Figure 2B) together with two master variants within the *S. latissima* samples in 2016–2018 (Figure 2C) show some variation, however. Moreover, when grouping these older *S. latissima* samples into three different areas (south, north, and fjords) (see Table 1), the differences were significant (ANOVA single-factor, F(2, 36) = [8.115932947], *p* = 0.001) (Figure 2D). A post hoc Tukey–Kramer test showed that there were significant differences between the groups South–Fjords and Fjords–North at *p* < 0.05, but not within the North–South samples.

**Figure 3:**
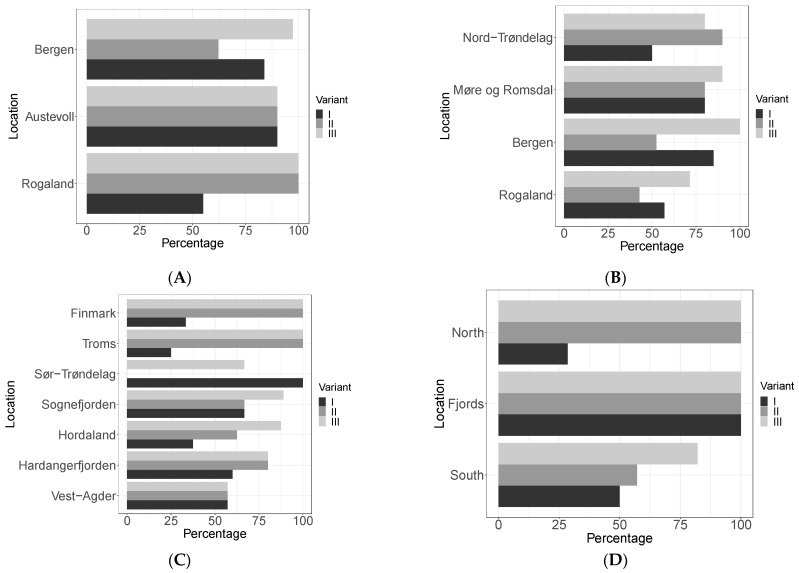
Percentage of each possible viral master variant for both kelp species, year, and location (**A**–**C**), or per area (**D**), on average. (**A**) *L. hyperborea* 2021–2022; (**B**) *S. latissima* 2021–2022; (**C**,**D**) *S. latissima* 2016–2018.

There were no significant differences between the percentage of each viral master variant and their location for *S. latissima* in 2021–2022 (*p* > 0.5) (Figure 3), *L. hyperborea* (*p* > 0.2) (Figure 3B), or for *S. latissima* in 2016–2018 (*p* > 0.2) (Figure 3C). It is worth mentioning that all three master variants were found in all the samples from Austevoll for *S. latissima* in 2021–2022 (Figure 3A) and that master variant III was represented in all samples (Figure 3A–C).

Even though no significant differences were found in the percentage of each viral master variant per area in the *S. latissima* samples from 2016 to 2018 (*p* > 0.2), there were significant differences within some groups (Figure 3D). This was the case for the northernmost stations (Finmark and Troms), where master variant I was significantly less represented than the other two (ANOVA, single-factor, F(2,18) = [15], *p* < 0.001) (Figure 3D). Also, within the southernmost sampling stations, master variant III was significantly more prevalent than the other two (ANOVA, single-factor, F(2,78) = [3.871], *p* = 0.02) (Figure 3D). In the case of the fjords, all three master variants were highly represented, with a prevalence of 100% for each of them in this area (Figure 3D). We have to remark that only a few *S. latissima* samples from 2016 to 2018 were sequenced; therefore, these results might not be representative. 

### 3.3. Phylogeny

**Figure 4:**
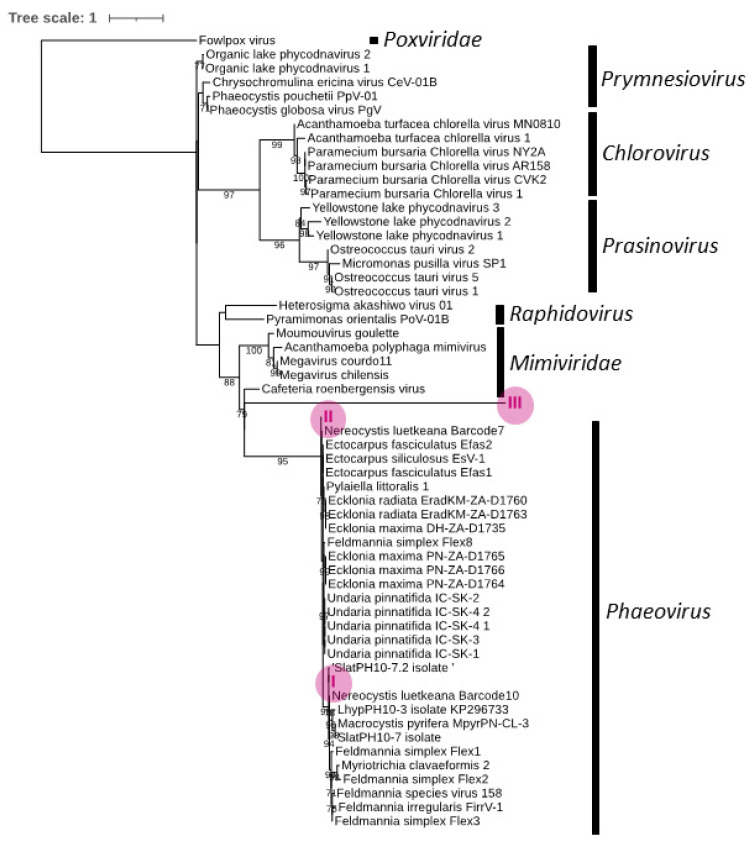
Maximum likelihood phylogenetic tree of the amplified phaeoviral MCP fragment from our three viral master variant protein sequences (I, II, and III, magenta), aligned with other known giant viruses (Nucleocytoviricota) (see Table S4 for accession numbers). Model of substitution: LG + G_4_. Nodes are bootstrap values (only bootstraps > 70 are shown), and branch lengths represent evolutionary distances. The tree is rooted with out-group Fowlpox virus.

The three master variants were translated and aligned with other known members of the Nucleocytoviricota [1] (Figure 4, Table S4). Master variants I and II fell within the phaeoviruses (bootstrap value = 95); more specifically, I clustered within phaeoviral sub-group C (boostrap value = 64), while II clustered within sub-group A (bootstrap value = 11) (Figure 4). However, III did not fall into the phaeovirus cluster, being placed outside the two strongly supported phaeovirus (bootstrap = 95) and mimivirus (bootstrap value = 100) lineages (Figure 4).

## 4. Discussion

### 4.1. A Novel Virus Sequence Found in Kelp

Our extensive surveillance along the Norwegian coast revealed that phaeoviruses are widespread and highly prevalent in both *Laminaria hyperborea* and *Saccharina latissima.* Although most sequences are phylogenetically placed within *Phaeovirus* as expected, a third master variant fell outside of *Phaeovirus*, but still within *Phycodnaviridae*. This third master variant thus represents a putative new member of an unclassified phycodnavirus genus. The comparable level of detection of this novel variant to the other *Phaeovirus* master variants indicates that the latent genome integration strategy of phaeoviruses is employed by other, yet unclassified, phycodnaviruses.

Master variant III has never previously been detected within brown algae and may be limited to Norwegian kelps. However, we cannot rule out that its detection was due to our choice of using the Qiagen’s HotStarTaq Master Mix Kit, which is known for its high sensitivity in PCR reactions. Master variant III may thus have passed undetected in earlier scans for viruses.

### 4.2. Host Range and Blurred Spatio-Temporal Frontiers

The host range and spatio-temporal distribution detected from our sequencing efforts are consistent with a recent microsatellite analysis of the host organism *S. latissima,* demonstrating the high genetic connectivity of *S. latissima* populations along the coast from the outer part of Oslofjorden to Mid-Norway and a relatively uniform genetic diversity between all stations [36]. High genetic connection is expected as the Norwegian coastal current mainly flows unidirectionally from Skagerrak and northwards along the coast (0.3 m s^−1^), causing rapid spore dispersal [36,54]. However, the archipelagos and fjords create a complex and dynamic environment [54], and the reduced connectivity between Southern and Northern Norway, possibly caused by a partial barrier for spore spreading created by the Lofoten archipelago [36], could also be retrieved from our viral samples, indicating that the Lofoten archipelago is not just a partial barrier for spore dispersal but also for northward viral dispersion. Alternatively, the lower prevalence of viruses in *S. latissima* in Northern Norway than in Southern Norway could also be due to a different environment here, with lower sea temperatures and a more pronounced seasonality.

The high genetic interconnection in Norwegian kelp not only promotes ubiquitous viruses but also implies that kelps are susceptible to closely related viral variants (Figure 2 and Figure 3). Despite limited knowledge about phaeoviral diversity and host ranges [12], we do know that members of the phaeovirus sub-group B (mainly viruses that infect the ectocapoid *Feldmannia*) evolved from sub-group A (containing the type virus EsV-1) through genome reduction and an accompanying loss of DNA proofreading capability [40]. Recent phylogenetic analyses of MCP fragments from different kelp species demonstrated a phaeoviral host range expansion [8,55]. Novel phaeoviral MCPs were found in *Ecklonia maxima*, *Ecklonia radiata*, and *Undaria pinnatifida* (all of them belonging to the Laminariales order), and the consequent phylogenetic analysis placed them within sub-group A [8]. The *Macrocystis pyrifera*, *Laminaria,* and *Saccharina* species (all of them from the Laminariales order) produced a cluster distinct from all known phaeoviruses, which was named sub-group C [7,8]. Our phylogenetic analysis placed master variant I within the phaeoviral sub-group C, while master variant II was placed within phaeoviral sub-group A (Figure 4). Similarly, two phaeoviral MCPs recovered from *Nereocystis luetkeana* (the kelp that dominates in Northern California, USA) clustered with phaeoviral sub-groups A and C [55]. These two viral sequences, like in our study, were found in single algal individuals [55]. Sub-group C appears to share a common ancestry with sub-groups A and B [7]; however, it is unknown if it diverged at the same time as sub-group B (during or after the speciation of the Ectocarpales) or during or after the divergence of Ectocarpales and Laminariales (90.5 Ma) [56,57]. MCPs from the genome of *Saccharina japonica* have also been found to be divergent from subgroups B and C and have therefore been defined as subgroup D [8]. These results continue to add pieces of information to the still-unknown phaeoviral phylogeny, encouraging future work into other brown algae orders.

### 4.3. Phaeoviral Prevalence on the Norwegian Coast and Climate Change

The significant increase in phaeoviral prevalence we show in the current study is in line with global warming predictions regarding host-pathogen interactions, which will likely become more frequent and intense in the future [34]. Sea surface temperatures (SST), which are more prominent at higher northern latitudes [58], and marine heatwaves (MHWs) have progressively increased over the last three decades [59] and are linked to kelp decline and community structure changes [60,61,62]. Filbee-Dexter and colleagues (2020) [63] experimentally demonstrated a relationship between MHWs and high kelp loss in southern Norway. Mortality was strongest linked to physiological changes, such as tissue damage, increased dislodgment, and reduced photosynthetic and reproductive performances when temperature exceeded lethal thresholds [63]. Early-life stages have also been found to be vulnerable to increased temperatures [64,65], even if both haplo- and diploid forms from different kelp species are presumably resilient [26,66]. In any case, a recent global analysis revealed that 38% of the world’s kelp forests have been in decline over the past five decades [24,26]. Between 2002 and 2009, more than 80% and 40% of the *S. latissima* populations disappeared from the Norwegian Skagerrak and the Norwegian West Coast, respectively, being replaced by turf algae [27]. Coastal eutrophication, rising sea temperatures, and/or predator–prey interactions were regarded as the most probable explanations [27]; however, some fluctuations are not easily explained by temperature or other environmental factors alone [28]. We propose the possibility that a changing climate with abrupt environmental changes may have brought about a higher induction of latent phaeoviruses, followed by, e.g., a high mortality of the recruitment stages.

**Figure 5:**
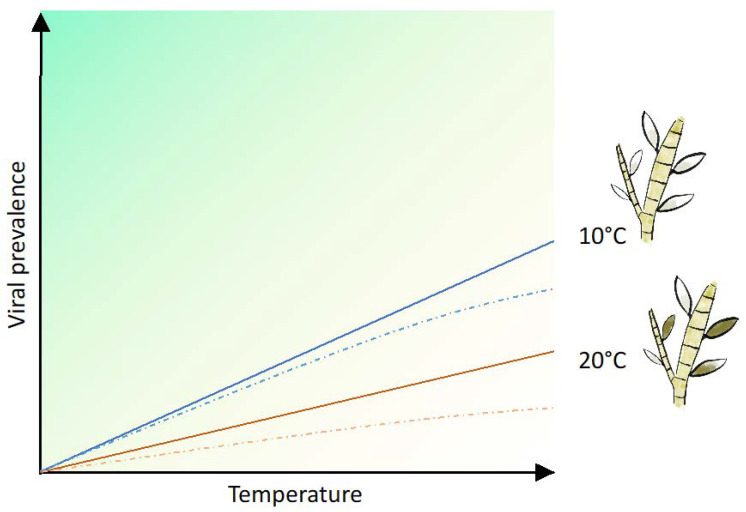
Theoretical model for virus prevalence under 10 °C (blue line) or 20 °C (orange line) over time. Dashed lines represent the actual viral prevalence reduction due to viral genome segregation after meiosis for each temperature.

Bacterial proviruses have been induced by UV radiation [67,68] and elevated salinity, but even though the effects of temperature on the transition from lysogenic to lytic lifestyles have also been studied [69,70], the potential effects are still unclear [71], especially for viruses infecting eukaryotic species. Whether the predicted higher temperatures and intense MHWs in Norwegian marine coastal waters [72,73] could stimulate the lysogenic-lytic switch to turn on or increase viral prevalence in kelp is not known. However, phaeoviral symptoms in members of Ectocarpales have been shown to be temperature-sensitive [13]. Müller and colleagues (1989) showed that cultures of *Ectocarpus siliculosus* presented abnormal gametangia (filled with viruses) at 10 °C, while between 15 °C and 20 °C, defective and normal gametangia were found [13]. The release of viruses could be stimulated by a rapid transfer of material from 12 °C to 20 °C, which would cause reproductive cells to burst [74]. Taking this model as an example, we can theoretically draw a multiple-scenario model to try to imagine how the viral prevalence will change over time in *E. siliculosus* (Figure 5). If we have a natural algal population and assume a 10% viral prevalence rate, we could predict a viral production and population increase (increased viral prevalence) at a temperature of 10 °C over time (Figure 5). However, if the new norm is 20 °C for the same population, the viral production, increase, and spread will not happen as fast (repression of prevalence) (Figure 5).

In view of the above, one could try to use this model to predict what would happen in kelp. However, even though there are many similarities between *E. siliculosus* and kelps regarding viral–host interactions and what is known about these, there are also some differences (Table 2). It is not known if phaeoviruses in kelps are triggered by temperature, or if they are, which temperature range may trigger them. Second, viral particles have not been found in vegetative cells in Ectocarpales [7] but are commonly occurring in vegetative cells of kelp gametophytes [7]. The gametophytic generation of *S. latissima* and *L. hyperborea* becomes established in autumn/winter and matures during spring, but under unfavorable conditions, gametophytes will continue to grow vegetatively and can thus probably persist in all seasons [75]. If the release of viruses is stimulated by a rapid temperature increase, like in *E. siliculosus*, vegetatively growing gametophytes of kelps could represent a large reservoir of viruses, given that the viruses have been activated by some factor.

## 5. Conclusions

Phaeoviruses are highly prevalent in *Saccharina latissima* and *Laminaria hyperborea* and widely distributed along the Norwegian coast. We found Norwegian kelp species to be coinfected by multiple viral variants, a subset of which represents a viral sequence previously not found that clustered in between *Mimivirus* and *Phaeovirus* MCPs. Phaeoviruses have co-evolved with their hosts on account of stable infections [16] that allow them to persist and evolve without killing their hosts. By integrating into the germinal line, phaeoviruses have almost certainly ensured their persistence through time, yet it is unclear if these multiple infections could somehow benefit or hinder their hosts. Even if these interactions may not be harmful for algae, they could still cause disease in cultivated algal crops, as seen in plants [76]. Lysogenic-to lytic switches are still poorly understood, and more research is needed to understand phaeoviral–kelp interactions, especially under future climate conditions. If these changes could actually trigger the induction of latent phaeoviruses, disrupting such a stable co-existence and causing more disease, the losses for the natural environment and aquaculture industry might be a serious problem in the future.

## Figures and Tables

**Table 1:** Sampling station locations and coordinates.

Location	Sampling Station	Latitude	Longitude	Area
Bud, Jøssingfjorden, Vest-Agder	A	58.308861	6.332444	South
Søgne, Vest-Agder	C	58.073583	7.837028	South
Kvam, Hardangerfjorden	D	60.392111	6.300194	Fjords
Porsanger, Finmark	E	70.297278	25.294500	North
Sommarøy, Troms	F	69.639222	18.017917	North
Krøttøy, Troms	G	69.069472	16.531444	North
Vengsøya, Troms	H	69.844972	18.588639	North
Solund, Sognefjorden	I	61.073931	4.831590	South
Frøya, Sør-Trøndelag	J	63.735389	8.845333	South
Korsfjorden, Hordaland	K	60.237907	5.238756	South
Leikanger, Sogn og Fjordane	So1	61.182194	6.784817	Fjords
Finnefjorden, Sogn og Fjordane	So3	61.048350	6.311100	Fjords
Høyanger, Sogn og Fjordane	So5	61.206100	6.060062	Fjords
Oppedalsvika, Sogn og Fjordane	So7	61.059367	5.512367	Fjords
Nyhamnarsundet, Sogn og Fjordane	So9	61.005300	5.012750	South
Kilstraumen, Hordaland	So10	60.800100	4.940267	South
Bårdholmen vest av Fitjar, Hordaland (Hardangerfjorden)	Ha1	59.896000	5.202667	South
Korsfjorden, Hordaland (*Laminaria hyperborea*)	K	60.157397	5.006340	South
Korsfjorden, Hordaland (*Saccharina latissima*)	K-2	60.240977	5.240332	South
Norheimsvågen, Karmøy, Rogaland.	N	59.378957	5.298491	South
Kvalsvikvegen, Haugesund, Rogaland	KV	59.437829	5.231808	South
Storøy, Karmøy, Rogaland	S	59.410729	5.234212	South
Vikna, Nord-Trondelag	V	64.054167	8.599167	South
Bona Sea Rogaland	B	59.156389	5.495556	South
Møre og Romsdal	M	62.481667	5.670833	South
Trollsøy, Austevoll	T	60.130350	5.248183	South

**Table 2:** Comparison between *Ectocarpus siliculosus* and kelp species regarding the current knowledge on viral–host interactions.

	*Ectocarpus siliculosus*	Kelp
Phaeoviruses present in reproductive structures (gametangia and sporangia).	yes	yes
Phaeoviruses present in vegetative cells.	no	yes
Phaeoviral symptoms are temperature-sensitive.	yes	**?**
Virus genome is duplicated during mitosis and segregates in a Mendelian fashion during meiosis.	yes	yes
Vertical and lateral (gametes or spores) viral transmission.	yes	yes

## Data Availability

All processed data used in the manuscript are presented in the manuscript figures, tables, and Supplemental Material.

## Supplementary Materials

The following supporting information can be downloaded at https://www.mdpi.com/article/10.3390/v15122331/s1: Figure S1: Example of an agarose gel electrophoresis analysis with six positive *Saccharina latissima* samples. *Nereocystis luetkeana* sample #18 was used as positive control [51]. Figure S2: The three final viral consensus sequences from our entire dataset (named master variant I, II and III) and their respective protein translations. Figure S3: Gen Bank’s Blastn search results for the three final viral nucleotide consensus sequences. Table S1: *S.latissima* samples (2021–2022). Table S2: *L. hyperborea* samples (2021–2022). Table S3: Sequencing sample information. Table S4: GenBank accession numbers. Table S5: Number of viral positive (+) and negative (−) samples for each kelp species and year, and in total. Table S6: Number of infected, non-infected and total number of samples of *S.latissima* from the different areas (defined in Table 1) from 2016–2018.
