# Supplementary material for: Phaeoviruses Present in Cultured and Natural Kelp Species, Saccharina latissima and Laminaria hyperborea (Phaeophyceae, Laminariales), in Norway
Source: Viruses. 2023 Nov 28;15(12):2331. doi: 10.3390/v15122331 (PMC10747701; doi:10.3390/v15122331)
Supplement: Supplementary file 1 [file viruses-15-02331-s001.zip › viruses-2709360-supplementary.pdf]

# Supplementary material

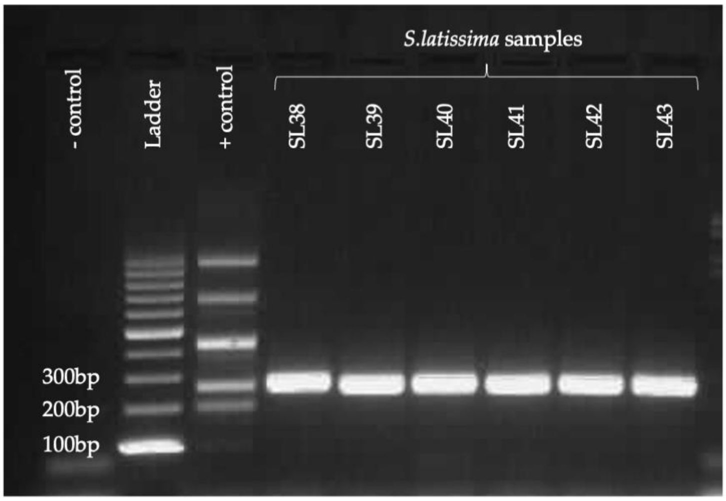


**Figure S1: Example of an agarose gel electrophoresis analysis with six positive *Saccharina latissima* samples. *Nereocystis luetkeana* sample #18 was used as positive control [51].**

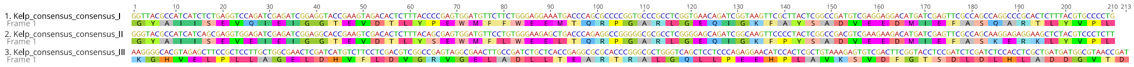


**Figure S2: The three final viral consensus sequences from our entire dataset (named master variant I, II and III) and their respective protein translations.**

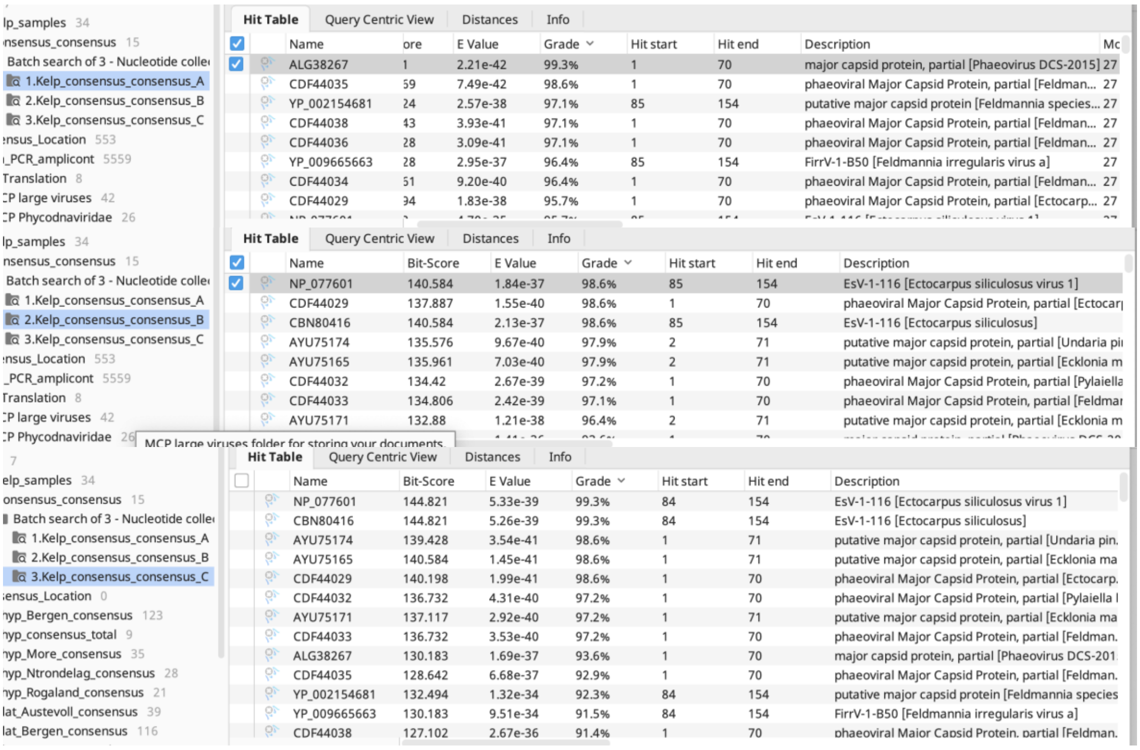

| Name | ore | E Value | Grade | Hit start | Hit end | Description | Mc |
|---|---|---|---|---|---|---|---|
| ALG38267 | 1 | 2.21e-42 | 99.3% | 1 | 70 | major capsid protein, partial [Phaeovirus DCS-2015] | 27 |
| CDF44035 | 59 | 7.49e-42 | 98.6% | 1 | 70 | phaeoviral Major Capsid Protein, partial [Feldman... | 27 |
| YP_002154681 | 24 | 2.57e-38 | 97.1% | 85 | 154 | putative major capsid protein [Feldmannia species... | 27 |
| CDF44038 | 43 | 3.93e-41 | 97.1% | 1 | 70 | phaeoviral Major Capsid Protein, partial [Feldman... | 27 |
| CDF44036 | 28 | 3.09e-41 | 97.1% | 1 | 70 | phaeoviral Major Capsid Protein, partial [Feldman... | 27 |
| YP_009665663 | 28 | 2.95e-37 | 96.4% | 85 | 154 | FirrV-1-B50 [Feldmannia irregularis virus a] | 27 |
| CDF44034 | 51 | 9.20e-40 | 96.4% | 1 | 70 | phaeoviral Major Capsid Protein, partial [Feldman... | 27 |
| CDF44029 | 34 | 1.83e-38 | 95.7% | 1 | 70 | phaeoviral Major Capsid Protein, partial [Ectocarp... | 27 |

| Name | Bit-Score | E Value | Grade | Hit start | Hit end | Description |
|---|---|---|---|---|---|---|
| NP_077601 | 140.584 | 1.84e-37 | 98.6% | 85 | 154 | EsV-1-116 [Ectocarpus siliculosus virus 1] |
| CDF44029 | 137.887 | 1.55e-40 | 98.6% | 1 | 70 | phaeoviral Major Capsid Protein, partial [Ectocar |
| CBN80416 | 140.584 | 2.13e-37 | 98.6% | 85 | 154 | EsV-1-116 [Ectocarpus siliculosus] |
| AYU75174 | 135.576 | 9.67e-40 | 97.9% | 2 | 71 | putative major capsid protein, partial [Undaria pi |
| AYU75165 | 135.961 | 7.03e-40 | 97.9% | 2 | 71 | putative major capsid protein, partial [Ecklonia m |
| CDF44032 | 134.42 | 2.67e-39 | 97.2% | 1 | 70 | phaeoviral Major Capsid Protein, partial [Pylaiella |
| CDF44033 | 134.806 | 2.42e-39 | 97.1% | 1 | 70 | phaeoviral Major Capsid Protein, partial [Feldmar |
| AYU75171 | 132.88 | 1.21e-38 | 96.4% | 2 | 71 | putative major capsid protein, partial [Ecklonia m |

| Name | Bit-Score | E Value | Grade | Hit start | Hit end | Description |
|---|---|---|---|---|---|---|
| NP_077601 | 144.821 | 5.33e-39 | 99.3% | 84 | 154 | EsV-1-116 [Ectocarpus siliculosus virus 1] |
| CBN80416 | 144.821 | 5.26e-39 | 99.3% | 84 | 154 | EsV-1-116 [Ectocarpus siliculosus] |
| AYU75174 | 139.428 | 3.54e-41 | 98.6% | 1 | 71 | putative major capsid protein, partial [Undaria pin. |
| AYU75165 | 140.584 | 1.45e-41 | 98.6% | 1 | 71 | putative major capsid protein, partial [Ecklonia ma |
| CDF44029 | 140.198 | 1.99e-41 | 98.6% | 1 | 70 | phaeoviral Major Capsid Protein, partial [Ectocarp. |
| CDF44032 | 136.732 | 4.31e-40 | 97.2% | 1 | 70 | phaeoviral Major Capsid Protein, partial [Pylaiella l |
| AYU75171 | 137.117 | 2.92e-40 | 97.2% | 1 | 71 | putative major capsid protein, partial [Ecklonia ma |
| CDF44033 | 136.732 | 3.53e-40 | 97.2% | 1 | 70 | phaeoviral Major Capsid Protein, partial [Feldman. |
| ALG38267 | 130.183 | 1.69e-37 | 93.6% | 1 | 70 | major capsid protein, partial [Phaeovirus DCS-201 |
| CDF44035 | 128.642 | 6.68e-37 | 92.9% | 1 | 70 | phaeoviral Major Capsid Protein, partial [Feldman. |
| YP_002154681 | 132.494 | 1.32e-34 | 92.3% | 84 | 154 | putative major capsid protein [Feldmannia species |
| YP_009665663 | 130.183 | 9.51e-34 | 91.5% | 84 | 154 | FirrV-1-B50 [Feldmannia irregularis virus a] |
| CDF44038 | 127.102 | 2.67e-36 | 91.4% | 1 | 70 | phaeoviral Major Capsid Protein, partial [Feldman. |

**Figure S3: Gen Bank's Blastn search results for the three final viral nucleotide consensus sequences.**

**Table S1:** ***S.latissima*** **samples (2021-2022).**

| Sample | Species | Date | Location | Lat | Long | Sampling station | Depth (m) | AV Ta (Celsius) | Salinity | Dry weight (mg) | DNA 1 (ng/ul) | Volume (ul) | DNA 2 (ng/ul) | Volume (ul) | Virus positive? |
|---|---|---|---|---|---|---|---|---|---|---|---|---|---|---|---|
| SL1 | *S. Latissima* | 04/05/2021 | Trollsøy, Austevoll | 60,13035 | 5,248183 | T | 0-5 | 9,4 | 33 | 21 | 4,48 | 100 | - | - | 0 |
| SL2 | *S. Latissima* | 04/05/2021 | Trollsøy, Austevoll | 60,13035 | 5,248183 | T | 0-5 | 9,4 | 33 | 20 | 3,64 | 100 | - | - | 1 |
| SL3 | *S. Latissima* | 04/05/2021 | Trollsøy, Austevoll | 60,13035 | 5,248183 | T | 0-5 | 9,4 | 33 | 17 | 2,84 | 50 | 3,82 | 50 | 1 |
| SL4 | *S. Latissima* | 04/05/2021 | Trollsøy, Austevoll | 60,13035 | 5,248183 | T | 0-5 | 9,4 | 33 | 17 | 4,98 | 50 | 4,18 | 50 | 1 |
| SL5 | *S. Latissima* | 04/05/2021 | Trollsøy, Austevoll | 60,13035 | 5,248183 | T | 0-5 | 9,4 | 33 | 19 | 8,56 | 50 | 5,92 | 50 | 0 |
| SL6 | *S. Latissima* | 04/05/2021 | Trollsøy, Austevoll | 60,13035 | 5,248183 | T | 0-5 | 9,4 | 33 | 15 | 2,26 | 50 | 5,36 | 50 | 1 |
| SL7 | *S. Latissima* | 04/05/2021 | Trollsøy, Austevoll | 60,13035 | 5,248183 | T | 0-5 | 9,4 | 33 | 17 | 10,8 | 50 | 4,92 | 50 | 1 |
| SL8 | *S. Latissima* | 04/05/2021 | Trollsøy, Austevoll | 60,13035 | 5,248183 | T | 0-5 | 9,4 | 33 | 20 | 8,6 | 50 | 3,66 | 50 | 0 |
| SL9 | *S. Latissima* | 04/05/2021 | Trollsøy, Austevoll | 60,13035 | 5,248183 | T | 0-5 | 9,4 | 33 | 14 | 3,86 | 50 | 3,58 | 50 | 0 |
| SL10 | *S. Latissima* | 04/05/2021 | Trollsøy, Austevoll | 60,13035 | 5,248183 | T | 0-5 | 9,4 | 33 | 19 | 4,32 | 50 | 4,28 | 50 | 0 |
| SL11 | *S. Latissima* | 04/05/2021 | Trollsøy, Austevoll | 60,13035 | 5,248183 | T | 0-5 | 9,4 | 33 | 20 | 11,3 | 50 | 3,42 | 50 | 1 |
| SL12 | *S. Latissima* | 04/05/2021 | Trollsøy, Austevoll | 60,13035 | 5,248183 | T | 0-5 | 9,4 | 33 | 13 | 5 | 50 | 5,3 | 50 | 0 |

| | | | | | | | | | | | | | | | |
|---|---|---|---|---|---|---|---|---|---|---|---|---|---|---|---|
| SL13 | *S. Latissima* | 04/05/2021 | Trollsøy, Austevoll | 60,13035 | 5,248183 | T | 0-5 | 9,4 | 33 | 17 | 2,5 | 50 | 3,36 | 50 | 1 |
| SL14 | *S. Latissima* | 04/05/2021 | Trollsøy, Austevoll | 60,13035 | 5,248183 | T | 0-5 | 9,4 | 33 | 24 | 9,74 | 50 | 4,7 | 50 | 1 |
| SL15 | *S. Latissima* | 04/05/2021 | Trollsøy, Austevoll | 60,13035 | 5,248183 | T | 0-5 | 9,4 | 33 | 20 | 11,4 | 50 | 2,28 | 50 | 1 |
| SL16 | *S. Latissima* | 04/05/2021 | Trollsøy, Austevoll | 60,13035 | 5,248183 | T | 0-5 | 9,4 | 33 | 19 | 4,94 | 50 | 1,87 | 50 | 1 |
| SL17 | *S. Latissima* | 04/05/2021 | Trollsøy, Austevoll | 60,13035 | 5,248183 | T | 0-5 | 9,4 | 33 | 16 | 20,6 | 50 | 6,7 | 50 | 1 |
| SL18 | *S. Latissima* | 04/05/2021 | Trollsøy, Austevoll | 60,13035 | 5,248183 | T | 0-5 | 9,4 | 33 | 19 | 5,58 | 50 | 5,54 | 50 | 1 |
| SL19 | *S. Latissima* | 04/05/2021 | Trollsøy, Austevoll | 60,13035 | 5,248183 | T | 0-5 | 9,4 | 33 | 12 | 6,62 | 50 | 2,9 | 50 | 0 |
| SL20 | *S. Latissima* | 04/05/2021 | Trollsøy, Austevoll | 60,13035 | 5,248183 | T | 0-5 | 9,4 | 33 | 14 | 6,56 | 50 | 3,7 | 50 | 1 |
| SL21 | *S. Latissima* | 04/05/2021 | Trollsøy, Austevoll | 60,13035 | 5,248183 | T | 0-5 | 9,4 | 33 | 15 | 4,98 | 50 | 4,06 | 50 | 1 |
| SL22 | *S. Latissima* | 04/05/2021 | Trollsøy, Austevoll | 60,13035 | 5,248183 | T | 0-5 | 9,4 | 33 | 18 | 6,66 | 50 | 5,16 | 50 | 1 |
| SL23 | *S. Latissima* | 04/05/2021 | Trollsøy, Austevoll | 60,13035 | 5,248183 | T | 0-5 | 9,4 | 33 | 14 | 7,32 | 50 | 4,58 | 50 | 1 |
| SL24 | *S. Latissima* | 04/05/2021 | Trollsøy, Austevoll | 60,13035 | 5,248183 | T | 0-5 | 9,4 | 33 | 23 | 3,66 | 50 | 1,66 | 50 | 1 |
| SL25 | *S. Latissima* | 04/05/2021 | Trollsøy, Austevoll | 60,13035 | 5,248183 | T | 0-5 | 9,4 | 33 | 16 | 3,62 | 50 | 4,3 | 50 | 1 |
| SL26 | *S. Latissima* | 04/05/2021 | Trollsøy, Austevoll | 60,13035 | 5,248183 | T | 0-5 | 9,4 | 33 | 25 | 13,8 | 50 | 9,82 | 50 | 1 |

| | | | | | | | | | | | | | | | |
|---|---|---|---|---|---|---|---|---|---|---|---|---|---|---|---|
| SL27 | *S. Latissima* | 04/05/2021 | Trollsøy, Austevoll | 60,13035 | 5,248183 | T | 0-5 | 9,4 | 33 | 23 | 9,68 | 50 | 4,78 | 50 | 1 |
| SL28 | *S. Latissima* | 04/05/2021 | Trollsøy, Austevoll | 60,13035 | 5,248183 | T | 0-5 | 9,4 | 33 | 22 | 12,3 | 50 | 7,02 | 50 | 1 |
| SL29 | *S. Latissima* | 04/05/2021 | Trollsøy, Austevoll | 60,13035 | 5,248183 | T | 0-5 | 9,4 | 33 | 9 | 48,6 | 50 | 33 | 50 | 1 |
| SL30 | *S. Latissima* | 04/05/2021 | Trollsøy, Austevoll | 60,13035 | 5,248183 | T | 0-5 | 9,4 | 33 | 23 | 14,3 | 50 | 7,78 | 50 | 1 |
| SL31 | *S. Latissima* | 04/05/2021 | Trollsøy, Austevoll | 60,13035 | 5,248183 | T | 0-5 | 9,4 | 33 | 22 | 11,8 | 50 | 6,44 | 50 | 1 |
| SL32 | *S. Latissima* | 04/05/2021 | Trollsøy, Austevoll | 60,13035 | 5,248183 | T | 0-5 | 9,4 | 33 | 21 | 45,2 | 50 | 21,6 | 50 | 1 |
| SL33 | *S. Latissima* | 04/05/2021 | Trollsøy, Austevoll | 60,13035 | 5,248183 | T | 0-5 | 9,4 | 33 | 23 | 26,8 | 50 | 5,28 | 50 | 1 |
| SL34 | *S. Latissima* | 04/05/2021 | Trollsøy, Austevoll | 60,13035 | 5,248183 | T | 0-5 | 9,4 | 33 | 22 | 14,2 | 50 | 8,54 | 50 | 1 |
| SL35 | *S. Latissima* | 04/05/2021 | Trollsøy, Austevoll | 60,13035 | 5,248183 | T | 0-5 | 9,4 | 33 | 23 | 31,4 | 50 | 9,16 | 50 | 1 |
| SL36 | *S. Latissima* | 04/05/2021 | Trollsøy, Austevoll | 60,13035 | 5,248183 | T | 0-5 | 9,4 | 33 | 22 | 8,22 | 50 | 3,66 | 50 | 1 |
| SL37 | *S. Latissima* | 04/05/2021 | Trollsøy, Austevoll | 60,13035 | 5,248183 | T | 0-5 | 9,4 | 33 | 22,5 | 10,2 | 50 | 5,24 | 50 | 1 |
| SL38 | *S. Latissima* | 04/05/2021 | Trollsøy, Austevoll | 60,13035 | 5,248183 | T | 0-5 | 9,4 | 33 | 17,1 | 7,38 | 50 | 5,78 | 50 | 1 |
| SL39 | *S. Latissima* | 04/05/2021 | Trollsøy, Austevoll | 60,13035 | 5,248183 | T | 0-5 | 9,4 | 33 | 15,4 | 12,5 | 50 | 2,36 | 50 | 1 |
| SL40 | *S. Latissima* | 04/05/2021 | Trollsøy, Austevoll | 60,13035 | 5,248183 | T | 0-5 | 9,4 | 33 | 23,9 | 9,98 | 50 | 2,58 | 50 | 1 |

| | | | | | | | | | | | | | | | |
|---|---|---|---|---|---|---|---|---|---|---|---|---|---|---|---|
| SL41 | *S. Latissima* | 04/05/2021 | Trollsøy, Austevoll | 60,13035 | 5,248183 | T | 0-5 | 9,4 | 33 | 24,9 | 5,84 | 50 | 6,02 | 50 | 1 |
| SL42 | *S. Latissima* | 04/05/2021 | Trollsøy, Austevoll | 60,13035 | 5,248183 | T | 0-5 | 9,4 | 33 | 20,8 | 2,06 | 50 | 2,52 | 50 | 1 |
| SL43 | *S. Latissima* | 04/05/2021 | Trollsøy, Austevoll | 60,13035 | 5,248183 | T | 0-5 | 9,4 | 33 | 22,8 | 12,6 | 50 | 10,5 | 50 | 1 |
| SL44 | *S. Latissima* | 04/05/2021 | Trollsøy, Austevoll | 60,13035 | 5,248183 | T | 0-5 | 9,4 | 33 | 23 | 1,85 | 50 | 6,52 | 50 | 1 |
| SL45 | *S. Latissima* | 04/05/2021 | Trollsøy, Austevoll | 60,13035 | 5,248183 | T | 0-5 | 9,4 | 33 | 21 | 12,1 | 50 | 4,32 | 50 | 1 |
| SL46 | *S. Latissima* | 04/05/2021 | Trollsøy, Austevoll | 60,13035 | 5,248183 | T | 0-5 | 9,4 | 33 | 21,4 | 6,94 | 50 | 1,03 | 50 | 1 |
| SL47 | *S. Latissima* | 04/05/2021 | Trollsøy, Austevoll | 60,13035 | 5,248183 | T | 0-5 | 9,4 | 33 | 12,8 | 4,08 | 50 | 2,3 | 50 | 1 |
| SL48 | *S. Latissima* | 04/05/2021 | Trollsøy, Austevoll | 60,13035 | 5,248183 | T | 0-5 | 9,4 | 33 | 20,7 | 4,88 | 50 | 7,14 | 50 | 1 |
| SL49 | *S. Latissima* | 04/05/2021 | Trollsøy, Austevoll | 60,13035 | 5,248183 | T | 0-5 | 9,4 | 33 | 22,1 | 18 | 50 | 13,3 | 50 | 1 |
| SL50 | *S. Latissima* | 04/05/2021 | Trollsøy, Austevoll | 60,13035 | 5,248183 | T | 0-5 | 9,4 | 33 | 21,4 | 4,46 | 50 | 5 | 50 | 1 |
| SL51 | *S. Latissima* | 04/05/2021 | Trollsøy, Austevoll | 60,13035 | 5,248183 | T | 0-5 | 9,4 | 33 | 17,7 | 8,52 | 50 | 2,26 | 50 | 1 |
| SL52 | *S. Latissima* | 04/05/2021 | Trollsøy, Austevoll | 60,13035 | 5,248183 | T | 0-5 | 9,4 | 33 | 15,6 | 5,38 | 50 | 4,84 | 50 | 1 |
| SL53 | *S. Latissima* | 04/05/2021 | Trollsøy, Austevoll | 60,13035 | 5,248183 | T | 0-5 | 9,4 | 33 | 20,1 | 10,7 | 50 | 6,52 | 50 | 1 |
| SL54 | *S. Latissima* | 04/05/2021 | Trollsøy, Austevoll | 60,13035 | 5,248183 | T | 0-5 | 9,4 | 33 | 17 | 12,9 | 50 | 5,12 | 50 | 1 |

| | | | | | | | | | | | | | | | |
|---|---|---|---|---|---|---|---|---|---|---|---|---|---|---|---|
| SL55 | *S. Latissima* | 04/05/2021 | Trollsøy, Austevoll | 60,13035 | 5,248183 | T | 0-5 | 9,4 | 33 | 17,5 | 5,28 | 50 | 3,68 | 50 | 1 |
| SL56 | *S. Latissima* | 04/05/2021 | Trollsøy, Austevoll | 60,13035 | 5,248183 | T | 0-5 | 9,4 | 33 | 21,1 | 10,6 | 50 | 8,18 | 50 | 1 |
| SL57 | *S. Latissima* | 04/05/2021 | Trollsøy, Austevoll | 60,13035 | 5,248183 | T | 0-5 | 9,4 | 33 | 16,8 | 1,79 | 50 | 2,42 | 50 | 1 |
| SL58 | *S. Latissima* | 04/05/2021 | Trollsøy, Austevoll | 60,13035 | 5,248183 | T | 0-5 | 9,4 | 33 | 11,5 | 5,06 | 50 | 3,46 | 50 | 1 |
| SL59 | *S. Latissima* | 04/05/2021 | Trollsøy, Austevoll | 60,13035 | 5,248183 | T | 0-5 | 9,4 | 33 | 19,4 | 2,2 | 50 | 2,86 | 50 | 1 |
| SL60 | *S. Latissima* | 04/05/2021 | Trollsøy, Austevoll | 60,13035 | 5,248183 | T | 0-5 | 9,4 | 33 | 19,3 | 7,04 | 50 | 4,1 | 50 | 1 |
| SL61 | *S. Latissima* | 04/05/2021 | Trollsøy, Austevoll | 60,13035 | 5,248183 | T | 0-5 | 9,4 | 33 | 19,1 | 4,42 | 50 | 4,2 | 50 | 1 |
| SL62 | *S. Latissima* | 23/01/2022 | Korsfjorden, Bergen, Hordaland | 60.240977 | 5.240332 | K-2 | 0-5 | 6,6 | 32 | 17,5 | 2,2 | 50 | 1,22 | 50 | 1 |
| SL63 | *S. Latissima* | 23/01/2022 | Korsfjorden, Bergen, Hordaland | 60.240977 | 5.240332 | K-2 | 0-5 | 6,6 | 32 | 23,6 | 15 | 50 | 2,4 | 50 | 1 |
| SL64 | *S. Latissima* | 23/01/2022 | Korsfjorden, Bergen, Hordaland | 60.240977 | 5.240332 | K-2 | 0-5 | 6,6 | 32 | 13 | 4,78 | 50 | 5,22 | 50 | 1 |
| SL65 | *S. Latissima* | 23/01/2022 | Korsfjorden, Bergen, Hordaland | 60.240977 | 5.240332 | K-2 | 0-5 | 6,6 | 32 | 17 | 17,9 | 50 | 4,2 | 50 | 1 |
| SL66 | *S. Latissima* | 23/01/2022 | Korsfjorden, Bergen, Hordaland | 60.240977 | 5.240332 | K-2 | 0-5 | 6,6 | 32 | 25,6 | 30 | 50 | 38,4 | 50 | 1 |
| SL67 | *S. Latissima* | 23/01/2022 | Korsfjorden, Bergen, Hordaland | 60.240977 | 5.240332 | K-2 | 0-5 | 6,6 | 32 | 27,3 | 19,3 | 50 | 12,1 | 50 | 0 |
| SL68 | *S. Latissima* | 23/01/2022 | Korsfjorden, Bergen, Hordaland | 60.240977 | 5.240332 | K-2 | 0-5 | 6,6 | 32 | 23,3 | 17,4 | 50 | 9,34 | 50 | 1 |

| | | | | | | | | | | | | | | | |
|---|---|---|---|---|---|---|---|---|---|---|---|---|---|---|---|
| SL69 | *S. Latissima* | 23/01/2022 | Korsfjorden, Bergen, Hordaland | 60.240977 | 5.240332 | K-2 | 0-5 | 6,6 | 32 | 21 | 23,6 | 50 | 29,2 | 50 | 1 |
| SL70 | *S. Latissima* | 23/01/2022 | Korsfjorden, Bergen, Hordaland | 60.240977 | 5.240332 | K-2 | 0-5 | 6,6 | 32 | 20,3 | 10,9 | 50 | 4,88 | 50 | 1 |
| SL71 | *S. Latissima* | 23/01/2022 | Korsfjorden, Bergen, Hordaland | 60.240977 | 5.240332 | K-2 | 0-5 | 6,6 | 32 | 19 | 11,2 | 50 | 4,24 | 50 | 1 |
| SL72 | *S. Latissima* | 23/01/2022 | Korsfjorden, Bergen, Hordaland | 60.240977 | 5.240332 | K-2 | 0-5 | 6,6 | 32 | 23,8 | 10,9 | 100 | | | 1 |
| SL73 | *S. Latissima* | 23/01/2022 | Korsfjorden, Bergen, Hordaland | 60.240977 | 5.240332 | K-2 | 0-5 | 6,6 | 32 | 17 | 7,6 | 100 | | | 1 |
| SL74 | *S. Latissima* | 23/01/2022 | Korsfjorden, Bergen, Hordaland | 60.240977 | 5.240332 | K-2 | 0-5 | 6,6 | 32 | 21 | 20,2 | 100 | | | 1 |
| SL75 | *S. Latissima* | 23/01/2022 | Korsfjorden, Bergen, Hordaland | 60.240977 | 5.240332 | K-2 | 0-5 | 6,6 | 32 | 16,6 | 11 | 100 | | | 1 |
| SL76 | *S. Latissima* | 23/01/2022 | Korsfjorden, Bergen, Hordaland | 60.240977 | 5.240332 | K-2 | 0-5 | 6,6 | 32 | 23,8 | 24,6 | 100 | | | 1 |
| SL77 | *S. Latissima* | 23/01/2022 | Korsfjorden, Bergen, Hordaland | 60.240977 | 5.240332 | K-2 | 0-5 | 6,6 | 32 | 17 | 7,5 | 100 | | | 1 |
| SL78 | *S. Latissima* | 23/01/2022 | Korsfjorden, Bergen, Hordaland | 60.240977 | 5.240332 | K-2 | 0-5 | 6,6 | 32 | 22 | 37,2 | 100 | | | 1 |
| SL79 | *S. Latissima* | 23/01/2022 | Korsfjorden, Bergen, Hordaland | 60.240977 | 5.240332 | K-2 | 0-5 | 6,6 | 32 | 19 | 11,9 | 100 | | | 1 |
| SL80 | *S. Latissima* | 23/01/2022 | Korsfjorden, Bergen, Hordaland | 60.240977 | 5.240332 | K-2 | 0-5 | 6,6 | 32 | 12 | 9,24 | 100 | | | 1 |
| SL81 | *S. Latissima* | 23/01/2022 | Korsfjorden, Bergen, Hordaland | 60.240977 | 5.240332 | K-2 | 0-5 | 6,6 | 32 | 21,7 | 26,6 | 100 | | | 1 |
| SL82 | *S. Latissima* | 23/01/2022 | Korsfjorden, Bergen, Hordaland | 60.240977 | 5.240332 | K-2 | 0-5 | 6,6 | 32 | 24,8 | 16 | 50 | 6,22 | 50 | 1 |

| | | | | | | | | | | | | | | | |
|---|---|---|---|---|---|---|---|---|---|---|---|---|---|---|---|
| SL83 | *S. Latissima* | 23/01/2022 | Korsfjorden, Bergen, Hordaland | 60.240977 | 5.240332 | K-2 | 0-5 | 6,6 | 32 | 15 | 5,16 | 50 | 1,95 | 50 | 1 |
| SL84 | *S. Latissima* | 23/01/2022 | Korsfjorden, Bergen, Hordaland | 60.240977 | 5.240332 | K-2 | 0-5 | 6,6 | 32 | 21,9 | 8 | 50 | 2,06 | 50 | 1 |
| SL85 | *S. Latissima* | 23/01/2022 | Korsfjorden, Bergen, Hordaland | 60.240977 | 5.240332 | K-2 | 0-5 | 6,6 | 32 | 24,5 | 3,16 | 50 | 3,18 | 50 | 1 |
| SL86 | *S. Latissima* | 23/01/2022 | Korsfjorden, Bergen, Hordaland | 60.240977 | 5.240332 | K-2 | 0-5 | 6,6 | 32 | 22,4 | 9,82 | 50 | 9,26 | 50 | 1 |
| SL87 | *S. Latissima* | 23/01/2022 | Korsfjorden, Bergen, Hordaland | 60.240977 | 5.240332 | K-2 | 0-5 | 6,6 | 32 | 18 | 6,18 | 50 | 1,15 | 50 | 1 |
| SL88 | *S. Latissima* | 23/01/2022 | Korsfjorden, Bergen, Hordaland | 60.240977 | 5.240332 | K-2 | 0-5 | 6,6 | 32 | 18,4 | 6,3 | 50 | 3,8 | 50 | 1 |
| SL89 | *S. Latissima* | 23/01/2022 | Korsfjorden, Bergen, Hordaland | 60.240977 | 5.240332 | K-2 | 0-5 | 6,6 | 32 | 19,4 | 5,64 | 50 | 1,54 | 50 | 1 |
| SL90 | *S. Latissima* | 23/01/2022 | Korsfjorden, Bergen, Hordaland | 60.240977 | 5.240332 | K-2 | 0-5 | 6,6 | 32 | 15 | 6,1 | 50 | 1,04 | 50 | 1 |
| SL91 | *S. Latissima* | 23/01/2022 | Korsfjorden, Bergen, Hordaland | 60.240977 | 5.240332 | K-2 | 0-5 | 6,6 | 32 | 30 | 23 | 50 | 12,8 | 50 | 1 |
| SL92 | *S. Latissima* | 23/01/2022 | Korsfjorden, Bergen, Hordaland | 60.240977 | 5.240332 | K-2 | 0-5 | 6,6 | 32 | 25 | 20 | 50 | 13,5 | 50 | 0 |
| SL93 | *S. Latissima* | 23/01/2022 | Korsfjorden, Bergen, Hordaland | 60.240977 | 5.240332 | K-2 | 0-5 | 6,6 | 32 | 19,4 | 15,3 | 50 | 3 | 50 | 1 |
| SL94 | *S. Latissima* | 23/01/2022 | Korsfjorden, Bergen, Hordaland | 60.240977 | 5.240332 | K-2 | 0-5 | 6,6 | 32 | 29 | 15,3 | 50 | 4,9 | 50 | 1 |
| SL95 | *S. Latissima* | 23/01/2022 | Korsfjorden, Bergen, Hordaland | 60.240977 | 5.240332 | K-2 | 0-5 | 6,6 | 32 | 28 | 10,3 | 50 | 7,02 | 50 | 1 |
| SL96 | *S. Latissima* | 23/01/2022 | Korsfjorden, Bergen, Hordaland | 60.240977 | 5.240332 | K-2 | 0-5 | 6,6 | 32 | 23 | 21,8 | 50 | 9,66 | 50 | 1 |

| | | | | | | | | | | | | | | | |
|---|---|---|---|---|---|---|---|---|---|---|---|---|---|---|---|
| SL97 | *S. Latissima* | 08/03/2022 | Korsfjorden, Bergen, Hordaland | 60.240977 | 5.240332 | K-2 | 0-5 | 6,0 | 32 | 25,3 | 51,2 | 50 | 11,3 | 50 | 1 |
| SL98 | *S. Latissima* | 08/03/2022 | Korsfjorden, Bergen, Hordaland | 60.240977 | 5.240332 | K-2 | 0-5 | 6,0 | 32 | 25,7 | 19 | 50 | 8,84 | 50 | 1 |
| SL99 | *S. Latissima* | 08/03/2022 | Korsfjorden, Bergen, Hordaland | 60.240977 | 5.240332 | K-2 | 0-5 | 6,0 | 32 | 17,2 | 5,14 | 50 | 1,8 | 50 | 1 |
| SL100 | *S. Latissima* | 08/03/2022 | Korsfjorden, Bergen, Hordaland | 60.240977 | 5.240332 | K-2 | 0-5 | 6,0 | 32 | 22,9 | 35 | 50 | 22,4 | 50 | 1 |
| SL101 | *S. Latissima* | 08/03/2022 | Korsfjorden, Bergen, Hordaland | 60.240977 | 5.240332 | K-2 | 0-5 | 6,0 | 32 | 22,4 | 21,2 | 50 | 10,6 | 50 | 1 |
| SL102 | *S. Latissima* | 08/03/2022 | Korsfjorden, Bergen, Hordaland | 60.240977 | 5.240332 | K-2 | 0-5 | 6,0 | 32 | 18,2 | 8,44 | 50 | 7,06 | 50 | 1 |
| SL103 | *S. Latissima* | 08/03/2022 | Korsfjorden, Bergen, Hordaland | 60.240977 | 5.240332 | K-2 | 0-5 | 6,0 | 32 | 21,2 | 8,8 | 50 | 5,76 | 50 | 1 |
| SL104 | *S. Latissima* | 08/03/2022 | Korsfjorden, Bergen, Hordaland | 60.240977 | 5.240332 | K-2 | 0-5 | 6,0 | 32 | 34,4 | 29,4 | 50 | 8,96 | 50 | 1 |
| SL105 | *S. Latissima* | 08/03/2022 | Korsfjorden, Bergen, Hordaland | 60.240977 | 5.240332 | K-2 | 0-5 | 6,0 | 32 | 27 | 21,8 | 50 | 19,7 | 50 | 1 |
| SL106 | *S. Latissima* | 08/03/2022 | Korsfjorden, Bergen, Hordaland | 60.240977 | 5.240332 | K-2 | 0-5 | 6,0 | 32 | 21,3 | 12,9 | 50 | 5,44 | 50 | 1 |
| SL107 | *S. Latissima* | 08/03/2022 | Korsfjorden, Bergen, Hordaland | 60.240977 | 5.240332 | K-2 | 0-5 | 6,0 | 32 | 29,3 | 10,6 | 50 | 9,12 | 50 | 1 |
| SL108 | *S. Latissima* | 08/03/2022 | Korsfjorden, Bergen, Hordaland | 60.240977 | 5.240332 | K-2 | 0-5 | 6,0 | 32 | 24,3 | 23,6 | 50 | 13 | 50 | 1 |
| SL109 | *S. Latissima* | 08/03/2022 | Korsfjorden, Bergen, Hordaland | 60.240977 | 5.240332 | K-2 | 0-5 | 6,0 | 32 | 19 | 9,76 | 50 | 3,06 | 50 | 1 |
| SL110 | *S. Latissima* | 08/03/2022 | Korsfjorden, Bergen, Hordaland | 60.240977 | 5.240332 | K-2 | 0-5 | 6,0 | 32 | 28 | 17,6 | 50 | 5,8 | 50 | 1 |

| | | | | | | | | | | | | | | | |
|---|---|---|---|---|---|---|---|---|---|---|---|---|---|---|---|
| SL111 | *S. Latissima* | 08/03/2022 | Korsfjorden, Bergen, Hordaland | 60.240977 | 5.240332 | K-2 | 0-5 | 6,0 | 32 | 36 | 32 | 50 | 19,3 | 50 | 1 |
| SL112 | *S. Latissima* | 08/03/2022 | Korsfjorden, Bergen, Hordaland | 60.240977 | 5.240332 | K-2 | 0-5 | 6,0 | 32 | 33,8 | 28 | 50 | 25,8 | 50 | 1 |
| SL113 | *S. Latissima* | 08/03/2022 | Korsfjorden, Bergen, Hordaland | 60.240977 | 5.240332 | K-2 | 0-5 | 6,0 | 32 | 22 | 6,74 | 50 | 4,38 | 50 | 1 |
| SL114 | *S. Latissima* | 08/03/2022 | Korsfjorden, Bergen, Hordaland | 60.240977 | 5.240332 | K-2 | 0-5 | 6,0 | 32 | 21,2 | 13,6 | 50 | 7,24 | 50 | 1 |
| SL115 | *S. Latissima* | 08/03/2022 | Korsfjorden, Bergen, Hordaland | 60.240977 | 5.240332 | K-2 | 0-5 | 6,0 | 32 | 28 | 11,8 | 50 | 4,56 | 50 | 1 |
| SL116 | *S. Latissima* | 08/03/2022 | Korsfjorden, Bergen, Hordaland | 60.240977 | 5.240332 | K-2 | 0-5 | 6,0 | 32 | 27 | 24 | 50 | 10,9 | 50 | 1 |
| SL117 | *S. Latissima* | 08/03/2022 | Korsfjorden, Bergen, Hordaland | 60.240977 | 5.240332 | K-2 | 0-5 | 6,0 | 32 | 22,4 | 4,24 | 50 | 2,9 | 50 | 1 |
| SL118 | *S. Latissima* | 08/03/2022 | Korsfjorden, Bergen, Hordaland | 60.240977 | 5.240332 | K-2 | 0-5 | 6,0 | 32 | 42,9 | 30,4 | 50 | 13,6 | 50 | 1 |
| SL119 | *S. Latissima* | 08/03/2022 | Korsfjorden, Bergen, Hordaland | 60.240977 | 5.240332 | K-2 | 0-5 | 6,0 | 32 | 26 | 16,2 | 50 | 16,3 | 50 | 0 |
| SL120 | *S. Latissima* | 08/03/2022 | Korsfjorden, Bergen, Hordaland | 60.240977 | 5.240332 | K-2 | 0-5 | 6,0 | 32 | 25,8 | 21,2 | 50 | 11,4 | 50 | 0 |
| SL121 | *S. Latissima* | 08/03/2022 | Korsfjorden, Bergen, Hordaland | 60.240977 | 5.240332 | K-2 | 0-5 | 6,0 | 32 | 27,6 | 19,1 | 50 | 18,8 | 50 | 1 |
| SL122 | *S. Latissima* | 08/03/2022 | Korsfjorden, Bergen, Hordaland | 60.240977 | 5.240332 | K-2 | 0-5 | 6,0 | 32 | 39,5 | 7,04 | 50 | 2,32 | 50 | 1 |
| SL123 | *S. Latissima* | 08/03/2022 | Korsfjorden, Bergen, Hordaland | 60.240977 | 5.240332 | K-2 | 0-5 | 6,0 | 32 | 21 | 6,5 | 50 | 3,5 | 50 | 1 |
| SL124 | *S. Latissima* | 08/03/2022 | Korsfjorden, Bergen, Hordaland | 60.240977 | 5.240332 | K-2 | 0-5 | 6,0 | 32 | 24,6 | 14,6 | 50 | 7,04 | 50 | 1 |

| | | | | | | | | | | | | | | | |
|---|---|---|---|---|---|---|---|---|---|---|---|---|---|---|---|
| SL125 | *S. Latissima* | 08/03/2022 | Korsfjorden, Bergen, Hordaland | 60.240977 | 5.240332 | K-2 | 0-5 | 6,0 | 32 | 29,7 | 18,9 | 50 | 7,94 | 50 | 1 |
| SL126 | *S. Latissima* | 08/03/2022 | Korsfjorden, Bergen, Hordaland | 60.240977 | 5.240332 | K-2 | 0-5 | 6,0 | 32 | 22 | 8,18 | 50 | 9,66 | 50 | 1 |
| SL127 | *S. Latissima* | 08/03/2022 | Korsfjorden, Bergen, Hordaland | 60.240977 | 5.240332 | K-2 | 0-5 | 6,0 | 32 | 41,2 | 19,9 | 50 | 4,34 | 50 | 1 |
| SL128 | *S. Latissima* | 08/03/2022 | Korsfjorden, Bergen, Hordaland | 60.240977 | 5.240332 | K-2 | 0-5 | 6,0 | 32 | 28 | 27,4 | 50 | 5,7 | 50 | 1 |
| SL129 | *S. Latissima* | 08/03/2022 | Korsfjorden, Bergen, Hordaland | 60.240977 | 5.240332 | K-2 | 0-5 | 6,0 | 32 | 24,4 | 19,9 | 50 | 3,2 | 50 | |
| SL130 | *S. Latissima* | 11/02/2022 | Kvalsvik, Haugesund, Rogaland | 59.437829 | 5.231808 | KV | 0-5 | 7,1 | 34 | 17,5 | 15,15 | 50 | 8,88 | 50 | 1 |
| SL131 | *S. Latissima* | 11/02/2022 | Kvalsvik, Haugesund, Rogaland | 59.437829 | 5.231808 | KV | 0-5 | 7,1 | 34 | 17,9 | 14,3 | 50 | 14,8 | 50 | 1 |
| SL132 | *S. Latissima* | 11/02/2022 | Kvalsvik, Haugesund, Rogaland | 59.437829 | 5.231808 | KV | 0-5 | 7,1 | 34 | 24,1 | 23 | 50 | 46,6 | 50 | 1 |
| SL133 | *S. Latissima* | 11/02/2022 | Kvalsvik, Haugesund, Rogaland | 59.437829 | 5.231808 | KV | 0-5 | 7,1 | 34 | 30,9 | 29,6 | 50 | 37,6 | 50 | 1 |
| SL134 | *S. Latissima* | 11/02/2022 | Kvalsvik, Haugesund, Rogaland | 59.437829 | 5.231808 | KV | 0-5 | 7,1 | 34 | 28,2 | 31,6 | 50 | 19,5 | 50 | 1 |
| SL135 | *S. Latissima* | 11/02/2022 | Kvalsvik, Haugesund, Rogaland | 59.437829 | 5.231808 | KV | 0-5 | 7,1 | 34 | 29,5 | 83,6 | 50 | 22,2 | 50 | 1 |
| SL136 | *S. Latissima* | 11/02/2022 | Kvalsvik, Haugesund, Rogaland | 59.437829 | 5.231808 | KV | 0-5 | 7,1 | 34 | 27,8 | 29,2 | 50 | 29,2 | 50 | 1 |
| SL137 | *S. Latissima* | 11/02/2022 | Kvalsvik, Haugesund, Rogaland | 59.437829 | 5.231808 | KV | 0-5 | 7,1 | 34 | 27,4 | 29,4 | 50 | 12 | 50 | 1 |
| SL138 | *S. Latissima* | 11/02/2022 | Kvalsvik, Haugesund, Rogaland | 59.437829 | 5.231808 | KV | 0-5 | 7,1 | 34 | 17,3 | 6,44 | 50 | 2,92 | 50 | 1 |

| | | | | | | | | | | | | | | | |
|---|---|---|---|---|---|---|---|---|---|---|---|---|---|---|---|
| SL139 | *S. Latissima* | 11/02/2022 | Kvalsvik, Haugesund, Rogaland | 59.437829 | 5.231808 | KV | 0-5 | 7,1 | 34 | 24,7 | 102 | 50 | 7,7 | 50 | 1 |
| SL140 | *S. Latissima* | 11/02/2022 | Kvalsvik, Haugesund, Rogaland | 59.437829 | 5.231808 | KV | 0-5 | 7,1 | 34 | 25,9 | 28 | 50 | 29,6 | 50 | 1 |
| SL141 | *S. Latissima* | 11/02/2022 | Kvalsvik, Haugesund, Rogaland | 59.437829 | 5.231808 | KV | 0-5 | 7,1 | 34 | 32,3 | 26,6 | 50 | 1603 | 50 | 1 |
| SL142 | *S. Latissima* | 11/02/2022 | Kvalsvik, Haugesund, Rogaland | 59.437829 | 5.231808 | KV | 0-5 | 7,1 | 34 | 21,2 | 17,9 | 50 | 11,8 | 50 | 1 |
| SL143 | *S. Latissima* | 11/02/2022 | Kvalsvik, Haugesund, Rogaland | 59.437829 | 5.231808 | KV | 0-5 | 7,1 | 34 | 24,6 | 37 | 50 | 8,62 | 50 | 1 |
| SL144 | *S. Latissima* | 11/02/2022 | Kvalsvik, Haugesund, Rogaland | 59.437829 | 5.231808 | KV | 0-5 | 7,1 | 34 | 26,7 | 28,2 | 50 | 10,7 | 50 | 1 |
| SL145 | *S. Latissima* | 11/02/2022 | Kvalsvik, Haugesund, Rogaland | 59.437829 | 5.231808 | KV | 0-5 | 7,1 | 34 | 29,1 | 20,4 | 50 | 12,4 | 50 | 1 |
| SL146 | *S. Latissima* | 11/02/2022 | Kvalsvik, Haugesund, Rogaland | 59.437829 | 5.231808 | KV | 0-5 | 7,1 | 34 | 19,6 | 8,14 | 50 | 1,93 | 50 | 1 |
| SL147 | *S. Latissima* | 11/02/2022 | Kvalsvik, Haugesund, Rogaland | 59.437829 | 5.231808 | KV | 0-5 | 7,1 | 34 | 28,7 | 7,22 | 50 | 1,91 | 50 | 1 |
| SL148 | *S. Latissima* | 11/02/2022 | Kvalsvik, Haugesund, Rogaland | 59.437829 | 5.231808 | KV | 0-5 | 7,1 | 34 | 32,2 | 50,8 | 50 | 9,16 | 50 | 1 |
| SL149 | *S. Latissima* | 11/02/2022 | Kvalsvik, Haugesund, Rogaland | 59.437829 | 5.231808 | KV | 0-5 | 7,1 | 34 | 30,1 | 108 | 50 | 16,9 | 50 | 1 |
| SL150 | *S. Latissima* | 11/02/2022 | Kvalsvik, Haugesund, Rogaland | 59.437829 | 5.231808 | KV | 0-5 | 7,1 | 34 | 30,7 | 13,4 | 50 | 2,84 | 50 | 1 |
| SL151 | *S. Latissima* | 11/02/2022 | Kvalsvik, Haugesund, Rogaland | 59.437829 | 5.231808 | KV | 0-5 | 7,1 | 34 | 24,6 | 100 | 50 | 31,8 | 50 | 1 |
| SL152 | *S. Latissima* | 11/02/2022 | Kvalsvik, Haugesund, Rogaland | 59.437829 | 5.231808 | KV | 0-5 | 7,1 | 34 | 25,5 | 31 | 50 | 15,9 | 50 | 1 |

| | | | | | | | | | | | | | | | |
|---|---|---|---|---|---|---|---|---|---|---|---|---|---|---|---|
| SL153 | *S. Latissima* | 11/02/2022 | Kvalsvik, Haugesund, Rogaland | 59.437829 | 5.231808 | KV | 0-5 | 7,1 | 34 | 33,3 | 76,8 | 50 | 52,6 | 50 | 1 |
| SL154 | *S. Latissima* | 11/02/2022 | Kvalsvik, Haugesund, Rogaland | 59.437829 | 5.231808 | KV | 0-5 | 7,1 | 34 | 33 | 71,8 | 50 | 53,8 | 50 | 1 |
| SL155 | *S. Latissima* | 11/02/2022 | Kvalsvik, Haugesund, Rogaland | 59.437829 | 5.231808 | KV | 0-5 | 7,1 | 34 | 33,8 | 11,9 | 50 | 9,4 | 50 | 1 |
| SL156 | *S. Latissima* | 11/02/2022 | Kvalsvik, Haugesund, Rogaland | 59.437829 | 5.231808 | KV | 0-5 | 7,1 | 34 | 25,3 | 14,4 | 50 | 10,6 | 50 | 1 |
| SL157 | *S. Latissima* | 11/02/2022 | Kvalsvik, Haugesund, Rogaland | 59.437829 | 5.231808 | KV | 0-5 | 7,1 | 34 | 26,6 | 10,9 | 50 | 6,44 | 50 | 1 |
| SL158 | *S. Latissima* | 11/02/2022 | Kvalsvik, Haugesund, Rogaland | 59.437829 | 5.231808 | KV | 0-5 | 7,1 | 34 | 16,7 | 4,74 | 50 | 1,96 | 50 | 1 |
| SL159 | *S. Latissima* | 11/02/2022 | Kvalsvik, Haugesund, Rogaland | 59.437829 | 5.231808 | KV | 0-5 | 7,1 | 34 | 22,5 | 9,54 | 50 | 2,62 | 50 | 1 |
| SL160 | *S. Latissima* | 11/02/2022 | Kvalsvik, Haugesund, Rogaland | 59.437829 | 5.231808 | KV | 0-5 | 7,1 | 34 | 18,7 | 54,4 | 50 | 13,4 | 50 | 1 |
| SL161 | *S. Latissima* | 11/02/2022 | Kvalsvik, Haugesund, Rogaland | 59.437829 | 5.231808 | KV | 0-5 | 7,1 | 34 | 30 | 41,2 | 50 | 29,4 | 50 | 1 |
| SL162 | *S. Latissima* | 11/02/2022 | Kvalsvik, Haugesund, Rogaland | 59.437829 | 5.231808 | KV | 0-5 | 7,1 | 34 | 22,5 | 65,6 | 50 | 11,8 | 50 | 1 |
| SL163 | *S. Latissima* | 11/02/2022 | Storøy, Karmøy, Rogaland | 59.410729 | 5.234212 | S | 0-5 | 7,1 | 34 | 17,9 | 6,04 | 50 | 8,52 | 50 | 1 |
| SL164 | *S. Latissima* | 11/02/2022 | Storøy, Karmøy, Rogaland | 59.410729 | 5.234212 | S | 0-5 | 7,1 | 34 | 26,4 | 19 | 50 | 16,8 | 50 | 1 |
| SL165 | *S. Latissima* | 11/02/2022 | Storøy, Karmøy, Rogaland | 59.410729 | 5.234212 | S | 0-5 | 7,1 | 34 | 33,4 | 30,4 | 50 | 27,2 | 50 | 1 |
| SL166 | *S. Latissima* | 11/02/2022 | Storøy, Karmøy, Rogaland | 59.410729 | 5.234212 | S | 0-5 | 7,1 | 34 | 20,4 | 20,6 | 50 | 14,2 | 50 | 1 |

| | | | | | | | | | | | | | | | |
|---|---|---|---|---|---|---|---|---|---|---|---|---|---|---|---|
| SL167 | *S. Latissima* | 11/02/2022 | Storøy, Karmøy, Rogaland | 59.410729 | 5.234212 | S | 0-5 | 7,1 | 34 | 28,5 | 24,8 | 50 | 4,84 | 50 | 1 |
| SL168 | *S. Latissima* | 11/02/2022 | Storøy, Karmøy, Rogaland | 59.410729 | 5.234212 | S | 0-5 | 7,1 | 34 | 24 | 5,44 | 50 | 4,66 | 50 | 1 |
| SL169 | *S. Latissima* | 11/02/2022 | Storøy, Karmøy, Rogaland | 59.410729 | 5.234212 | S | 0-5 | 7,1 | 34 | 34,6 | 7,68 | 50 | 8,68 | 50 | 1 |
| SL170 | *S. Latissima* | 11/02/2022 | Storøy, Karmøy, Rogaland | 59.410729 | 5.234212 | S | 0-5 | 7,1 | 34 | 21,8 | 5,28 | 50 | 7,2 | 50 | 1 |
| SL171 | *S. Latissima* | 11/02/2022 | Storøy, Karmøy, Rogaland | 59.410729 | 5.234212 | S | 0-5 | 7,1 | 34 | 30 | 11,4 | 50 | 4,26 | 50 | 1 |
| SL172 | *S. Latissima* | 11/02/2022 | Storøy, Karmøy, Rogaland | 59.410729 | 5.234212 | S | 0-5 | 7,1 | 34 | 25,4 | 6,16 | 50 | 2,84 | 50 | 1 |
| SL173 | *S. Latissima* | 11/02/2022 | Storøy, Karmøy, Rogaland | 59.410729 | 5.234212 | S | 0-5 | 7,1 | 34 | 19,1 | 3,9 | 50 | 6,4 | 50 | 1 |
| SL174 | *S. Latissima* | 11/02/2022 | Storøy, Karmøy, Rogaland | 59.410729 | 5.234212 | S | 0-5 | 7,1 | 34 | 18,2 | 5 | 50 | 2,18 | 50 | 1 |
| SL175 | *S. Latissima* | 11/02/2022 | Storøy, Karmøy, Rogaland | 59.410729 | 5.234212 | S | 0-5 | 7,1 | 34 | 33,5 | 27,6 | 50 | 20,6 | 50 | 1 |
| SL176 | *S. Latissima* | 11/02/2022 | Storøy, Karmøy, Rogaland | 59.410729 | 5.234212 | S | 0-5 | 7,1 | 34 | 31,5 | 52 | 50 | 19,3 | 50 | 1 |
| SL177 | *S. Latissima* | 11/02/2022 | Storøy, Karmøy, Rogaland | 59.410729 | 5.234212 | S | 0-5 | 7,1 | 34 | 32,2 | 24,6 | 50 | 2,2 | 50 | 1 |
| SL178 | *S. Latissima* | 11/02/2022 | Storøy, Karmøy, Rogaland | 59.410729 | 5.234212 | S | 0-5 | 7,1 | 34 | 18,6 | 3,56 | 50 | 2,42 | 50 | 1 |
| SL179 | *S. Latissima* | 11/02/2022 | Storøy, Karmøy, Rogaland | 59.410729 | 5.234212 | S | 0-5 | 7,1 | 34 | 33,8 | 10,9 | 50 | 13 | 50 | 1 |
| SL180 | *S. Latissima* | 11/02/2022 | Storøy, Karmøy, Rogaland | 59.410729 | 5.234212 | S | 0-5 | 7,1 | 34 | 24,9 | 17,3 | 50 | 1,62 | 50 | 1 |

| | | | | | | | | | | | | | | | |
|---|---|---|---|---|---|---|---|---|---|---|---|---|---|---|---|
| SL181 | *S. Latissima* | 11/02/2022 | Storøy, Karmøy, Rogaland | 59.410729 | 5.234212 | S | 0-5 | 7,1 | 34 | 18,3 | 4,76 | 50 | 6,82 | 50 | 1 |
| SL182 | *S. Latissima* | 11/02/2022 | Storøy, Karmøy, Rogaland | 59.410729 | 5.234212 | S | 0-5 | 7,1 | 34 | 36,4 | 72,6 | 50 | 13,9 | 50 | 1 |
| SL183 | *S. Latissima* | 11/02/2022 | Storøy, Karmøy, Rogaland | 59.410729 | 5.234212 | S | 0-5 | 7,1 | 34 | 22,9 | 39,8 | 50 | 38,8 | 50 | 1 |
| SL184 | *S. Latissima* | 11/02/2022 | Storøy, Karmøy, Rogaland | 59.410729 | 5.234212 | S | 0-5 | 7,1 | 34 | 32,9 | 27,8 | 50 | 13,3 | 50 | 1 |
| SL185 | *S. Latissima* | 11/02/2022 | Storøy, Karmøy, Rogaland | 59.410729 | 5.234212 | S | 0-5 | 7,1 | 34 | 28,9 | 106 | 50 | 25,8 | 50 | 1 |
| SL186 | *S. Latissima* | 11/02/2022 | Storøy, Karmøy, Rogaland | 59.410729 | 5.234212 | S | 0-5 | 7,1 | 34 | 18,6 | 12,6 | 50 | 2,02 | 50 | 1 |
| SL187 | *S. Latissima* | 11/02/2022 | Storøy, Karmøy, Rogaland | 59.410729 | 5.234212 | S | 0-5 | 7,1 | 34 | 29 | 15 | 50 | 4,36 | 50 | 1 |
| SL188 | *S. Latissima* | 11/02/2022 | Kvalsvik, Haugesund, Rogaland | 59.437829 | 5.231808 | KV | 0-5 | 7,1 | 34 | 33,6 | 18,9 | 50 | 11,8 | 50 | 1 |
| SL189 | *S. Latissima* | 11/02/2022 | Kvalsvik, Haugesund, Rogaland | 59.437829 | 5.231808 | KV | 0-5 | 7,1 | 34 | 26,6 | 40,6 | 50 | 6,98 | 50 | 1 |
| SL190 | *S. Latissima* | 11/02/2022 | Kvalsvik, Haugesund, Rogaland | 59.437829 | 5.231808 | KV | 0-5 | 7,1 | 34 | 36,8 | 76,2 | 50 | 17,6 | 50 | 1 |
| SL191 | *S. Latissima* | 11/02/2022 | Kvalsvik, Haugesund, Rogaland | 59.437829 | 5.231808 | KV | 0-5 | 7,1 | 34 | 27,5 | 44,2 | 50 | 39,2 | 50 | 1 |
| SL192 | *S. Latissima* | 11/02/2022 | Kvalsvik, Haugesund, Rogaland | 59.437829 | 5.231808 | KV | 0-5 | 7,1 | 34 | 26,4 | 9,58 | 50 | 4,36 | 50 | 1 |
| SL193 | *S. Latissima* | 11/02/2022 | Kvalsvik, Haugesund, Rogaland | 59.437829 | 5.231808 | KV | 0-5 | 7,1 | 34 | 21,9 | 24,2 | 50 | 15,9 | 50 | 1 |
| SL194 | *S. Latissima* | 11/02/2022 | Kvalsvik, Haugesund, Rogaland | 59.437829 | 5.231808 | KV | 0-5 | 7,1 | 34 | 29,8 | 46,8 | 50 | 10,1 | 50 | 1 |

| | | | | | | | | | | | | | |
|---|---|---|---|---|---|---|---|---|---|---|---|---|---|
| SL195 | *S. Latissima* | 11/02/2022 | Storøy, Karmøy, Rogaland | 59.410729 | 5.234212 | S | 0-5 | 7,1 | 34 | 22,6 | 51 | 50 | 15,6 | 50 | 1 |
| SL196 | *S. Latissima* | 11/02/2022 | Storøy, Karmøy, Rogaland | 59.410729 | 5.234212 | S | 0-5 | 7,1 | 34 | 24,1 | 73 | 50 | 27,2 | 50 | 1 |
| SL197 | *S. Latissima* | 11/02/2022 | Storøy, Karmøy, Rogaland | 59.410729 | 5.234212 | S | 0-5 | 7,1 | 34 | 27,2 | 61 | 50 | 60 | 50 | 1 |
| SL198 | *S. Latissima* | 11/02/2022 | Storøy, Karmøy, Rogaland | 59.410729 | 5.234212 | S | 0-5 | 7,1 | 34 | 32,4 | 27 | 50 | 9,76 | 50 | 1 |
| SL199 | *S. Latissima* | 11/02/2022 | Norheimsvågen, Karmøy, Rogaland. | 59.378957 | 5.298491 | N | 0-5 | 6,7 | 34 | 25,4 | 17,1 | 50 | 15,6 | 50 | 1 |
| SL200 | *S. Latissima* | 11/02/2022 | Norheimsvågen, Karmøy, Rogaland. | 59.378957 | 5.298491 | N | 0-5 | 6,7 | 34 | 27,8 | 25,8 | 50 | 6,74 | 50 | 1 |
| SL201 | *S. Latissima* | 11/02/2022 | Norheimsvågen, Karmøy, Rogaland. | 59.378957 | 5.298491 | N | 0-5 | 6,7 | 34 | 18,5 | 5,24 | 50 | 3,1 | 50 | 1 |
| SL202 | *S. Latissima* | 11/02/2022 | Norheimsvågen, Karmøy, Rogaland. | 59.378957 | 5.298491 | N | 0-5 | 6,7 | 34 | 14,4 | 3,74 | 50 | 1,73 | 50 | 1 |
| SL203 | *S. Latissima* | 11/02/2022 | Norheimsvågen, Karmøy, Rogaland. | 59.378957 | 5.298491 | N | 0-5 | 6,7 | 34 | 32,4 | 8,66 | 50 | 6,96 | 50 | 1 |
| SL204 | *S. Latissima* | 11/02/2022 | Norheimsvågen, Karmøy, Rogaland. | 59.378957 | 5.298491 | N | 0-5 | 6,7 | 34 | 23,2 | 8,54 | 50 | 6,68 | 50 | 0 |
| SL205 | *S. Latissima* | 11/02/2022 | Norheimsvågen, Karmøy, Rogaland. | 59.378957 | 5.298491 | N | 0-5 | 6,7 | 34 | 18,4 | 5,98 | 50 | 5,4 | 50 | 1 |
| SL206 | *S. Latissima* | 11/02/2022 | Norheimsvågen, Karmøy, Rogaland. | 59.378957 | 5.298491 | N | 0-5 | 6,7 | 34 | 32,6 | 59,4 | 50 | 16,6 | 50 | 1 |
| SL207 | *S. Latissima* | 11/02/2022 | Norheimsvågen, Karmøy, Rogaland. | 59.378957 | 5.298491 | N | 0-5 | 6,7 | 34 | 32,2 | 16,4 | 50 | 8,14 | 50 | 1 |
| SL208 | *S. Latissima* | 11/02/2022 | Norheimsvågen, Karmøy, Rogaland. | 59.378957 | 5.298491 | N | 0-5 | 6,7 | 34 | 22 | 17,6 | 50 | 4,36 | 50 | 1 |

| | | | | | | | | | | | | | | | |
|---|---|---|---|---|---|---|---|---|---|---|---|---|---|---|---|
| SL209 | *S. Latissima* | 11/02/2022 | Norheimsvågen, Karmøy, Rogaland. | 59.378957 | 5.298491 | N | 0-5 | 6,7 | 34 | 18,8 | 10,1 | 50 | 3,74 | 50 | 1 |
| SL210 | *S. Latissima* | 11/02/2022 | Norheimsvågen, Karmøy, Rogaland. | 59.378957 | 5.298491 | N | 0-5 | 6,7 | 34 | 20,8 | 13,3 | 50 | 6,06 | 50 | 1 |
| SL211 | *S. Latissima* | 11/02/2022 | Norheimsvågen, Karmøy, Rogaland. | 59.378957 | 5.298491 | N | 0-5 | 6,7 | 34 | 22,9 | 13,9 | 50 | 5,54 | 50 | 1 |
| SL212 | *S. Latissima* | 11/02/2022 | Norheimsvågen, Karmøy, Rogaland. | 59.378957 | 5.298491 | N | 0-5 | 6,7 | 34 | 22,9 | 13,9 | 50 | 7,38 | 50 | 1 |
| SL213 | *S. Latissima* | 11/02/2022 | Norheimsvågen, Karmøy, Rogaland. | 59.378957 | 5.298491 | N | 0-5 | 6,7 | 34 | 23,6 | 11,1 | 50 | 3,72 | 50 | 1 |
| SL214 | *S. Latissima* | 11/02/2022 | Norheimsvågen, Karmøy, Rogaland. | 59.378957 | 5.298491 | N | 0-5 | 6,7 | 34 | 22,4 | 15,1 | 50 | 8,5 | 50 | 1 |
| SL215 | *S. Latissima* | 11/02/2022 | Norheimsvågen, Karmøy, Rogaland. | 59.378957 | 5.298491 | N | 0-5 | 6,7 | 34 | 20,3 | 43,6 | 50 | 22,2 | 50 | 1 |
| SL216 | *S. Latissima* | 11/02/2022 | Norheimsvågen, Karmøy, Rogaland. | 59.378957 | 5.298491 | N | 0-5 | 6,7 | 34 | 17,4 | 4,52 | 50 | 4,7 | 50 | 1 |
| SL217 | *S. Latissima* | 11/02/2022 | Norheimsvågen, Karmøy, Rogaland. | 59.378957 | 5.298491 | N | 0-5 | 6,7 | 34 | 34,2 | 23,2 | 50 | 12 | 50 | 1 |
| SL218 | *S. Latissima* | 11/02/2022 | Norheimsvågen, Karmøy, Rogaland. | 59.378957 | 5.298491 | N | 0-5 | 6,7 | 34 | 31,1 | 25,2 | 50 | 5,66 | 50 | 1 |
| SL219 | *S. Latissima* | 11/02/2022 | Norheimsvågen, Karmøy, Rogaland. | 59.378957 | 5.298491 | N | 0-5 | 6,7 | 34 | 22,5 | 8,46 | 50 | 7,58 | 50 | 1 |
| SL220 | *S. Latissima* | 11/02/2022 | Norheimsvågen, Karmøy, Rogaland. | 59.378957 | 5.298491 | N | 0-5 | 6,7 | 34 | 25 | 11,7 | 50 | 5,94 | 50 | 1 |
| SL221 | *S. Latissima* | 11/02/2022 | Norheimsvågen, Karmøy, Rogaland. | 59.378957 | 5.298491 | N | 0-5 | 6,7 | 34 | 19,9 | 13,7 | 50 | 5,2 | 50 | 1 |
| SL222 | *S. Latissima* | 11/02/2022 | Norheimsvågen, Karmøy, Rogaland. | 59.378957 | 5.298491 | N | 0-5 | 6,7 | 34 | 24,4 | 7,66 | 50 | 13,1 | 50 | 1 |

| | | | | | | | | | | | | | | | |
|---|---|---|---|---|---|---|---|---|---|---|---|---|---|---|---|
| SL223 | *S. Latissima* | 11/02/2022 | Norheimsvågen, Karmøy, Rogaland. | 59.378957 | 5.298491 | N | 0-5 | 6,7 | 34 | 17 | 36,8 | 50 | 8,38 | 50 | 1 |
| SL224 | *S. Latissima* | 11/02/2022 | Norheimsvågen, Karmøy, Rogaland. | 59.378957 | 5.298491 | N | 0-5 | 6,7 | 34 | 30,7 | 34,4 | 50 | 7,5 | 50 | 1 |
| SL225 | *S. Latissima* | 11/02/2022 | Norheimsvågen, Karmøy, Rogaland. | 59.378957 | 5.298491 | N | 0-5 | 6,7 | 34 | 29,9 | 30,2 | 50 | 13,7 | 50 | 1 |
| SL226 | *S. Latissima* | 11/02/2022 | Norheimsvågen, Karmøy, Rogaland. | 59.378957 | 5.298491 | N | 0-5 | 6,7 | 34 | 23,8 | 12 | 50 | 4,4 | 50 | 1 |
| SL227 | *S. Latissima* | 11/02/2022 | Norheimsvågen, Karmøy, Rogaland. | 59.378957 | 5.298491 | N | 0-5 | 6,7 | 34 | 17,6 | 6,96 | 50 | 5,12 | 50 | 1 |
| SL228 | *S. Latissima* | 11/02/2022 | Norheimsvågen, Karmøy, Rogaland. | 59.378957 | 5.298491 | N | 0-5 | 6,7 | 34 | 27,2 | 11,4 | 50 | 3,06 | 50 | 1 |
| SL229 | *S. Latissima* | 11/02/2022 | Norheimsvågen, Karmøy, Rogaland. | 59.378957 | 5.298491 | N | 0-5 | 6,7 | 34 | 17,5 | 7,82 | 50 | 5,68 | 50 | 1 |
| SL230 | *S. Latissima* | 11/02/2022 | Norheimsvågen, Karmøy, Rogaland. | 59.378957 | 5.298491 | N | 0-5 | 6,7 | 34 | 20,7 | 10,7 | 50 | 8,78 | 50 | 1 |
| SL231 | *S. Latissima* | 11/02/2022 | Norheimsvågen, Karmøy, Rogaland. | 59.378957 | 5.298491 | N | 0-5 | 6,7 | 34 | 32,4 | 79 | 50 | 13 | 50 | 0 |
| SL232 | *S. Latissima* | 11/02/2022 | Norheimsvågen, Karmøy, Rogaland. | 59.378957 | 5.298491 | N | 0-5 | 6,7 | 34 | 23,3 | 15,6 | 50 | 14,9 | 50 | 1 |
| SL233 | *S. Latissima* | 11/02/2022 | Storøy, Karmøy, Rogaland | 59.410729 | 5.234212 | S | 0-5 | 7,1 | 34 | 24,9 | 31,8 | 50 | 7,78 | 50 | 1 |
| SL234 | *S. Latissima* | 11/02/2022 | Storøy, Karmøy, Rogaland | 59.410729 | 5.234212 | S | 0-5 | 7,1 | 34 | 25,1 | 66,4 | 50 | 16,3 | 50 | 1 |
| SL235 | *S. Latissima* | 11/02/2022 | Norheimsvågen, Karmøy, Rogaland. | 59.378957 | 5.298491 | N | 0-5 | 6,7 | 34 | 25,8 | 10,2 | 50 | 10 | 50 | 0 |
| SL236 | *S. Latissima* | 11/02/2022 | Norheimsvågen, Karmøy, Rogaland. | 59.378957 | 5.298491 | N | 0-5 | 6,7 | 34 | 22,1 | 34 | 50 | 8,28 | 50 | 1 |

| | | | | | | | | | | | | | | | |
|---|---|---|---|---|---|---|---|---|---|---|---|---|---|---|---|
| SL237 | *S. Latissima* | 11/02/2022 | Norheimsvågen, Karmøy, Rogaland. | 59.378957 | 5.298491 | N | 0-5 | 6,7 | 34 | 25,4 | 8,32 | 50 | 12 | 50 | 1 |
| SL238 | *S. Latissima* | 11/02/2022 | Norheimsvågen, Karmøy, Rogaland. | 59.378957 | 5.298491 | N | 0-5 | 6,7 | 34 | 31,1 | 24,4 | 50 | 12,2 | 50 | 1 |
| SL239 | *S. Latissima* | 11/02/2022 | Norheimsvågen, Karmøy, Rogaland. | 59.378957 | 5.298491 | N | 0-5 | 6,7 | 34 | 17,7 | 32 | 50 | 22,6 | 50 | 1 |
| SL240 | *S. Latissima* | 11/02/2022 | Norheimsvågen, Karmøy, Rogaland. | 59.378957 | 5.298491 | N | 0-5 | 6,7 | 34 | 25,5 | 17,5 | 50 | 11,3 | 50 | 1 |
| SL241 | *S. Latissima* | 11/02/2022 | Norheimsvågen, Karmøy, Rogaland. | 59.378957 | 5.298491 | N | 0-5 | 6,7 | 34 | 29,1 | 10,2 | 50 | 11,5 | 50 | 1 |
| SL242 | *S. Latissima* | 11/02/2022 | Norheimsvågen, Karmøy, Rogaland. | 59.378957 | 5.298491 | N | 0-5 | 6,7 | 34 | 31,2 | 12,8 | 50 | 14 | 50 | 1 |
| SL243 | *S. Latissima* | 11/02/2022 | Norheimsvågen, Karmøy, Rogaland. | 59.378957 | 5.298491 | N | 0-5 | 6,7 | 34 | 27,1 | 29,6 | 50 | 5,32 | 50 | 1 |
| SL244 | *S. Latissima* | 11/02/2022 | Norheimsvågen, Karmøy, Rogaland. | 59.378957 | 5.298491 | N | 0-5 | 6,7 | 34 | 34,1 | 13,4 | 50 | 8,28 | 50 | 1 |
| SL245 | *S. Latissima* | 11/02/2022 | Norheimsvågen, Karmøy, Rogaland. | 59.378957 | 5.298491 | N | 0-5 | 6,7 | 34 | 26,9 | 31 | 50 | 22,4 | 50 | 1 |
| SL246 | *S. Latissima* | 11/02/2022 | Norheimsvågen, Karmøy, Rogaland. | 59.378957 | 5.298491 | N | 0-5 | 6,7 | 34 | 18,9 | 15,2 | 50 | 15,6 | 50 | 1 |
| SL247 | *S. Latissima* | 11/02/2022 | Norheimsvågen, Karmøy, Rogaland. | 59.378957 | 5.298491 | N | 0-5 | 6,7 | 34 | 32,1 | 21,8 | 50 | 11,8 | 50 | 1 |
| SL248 | *S. Latissima* | 11/02/2022 | Norheimsvågen, Karmøy, Rogaland. | 59.378957 | 5.298491 | N | 0-5 | 6,7 | 34 | 40,9 | 118 | 50 | 66,8 | 50 | 1 |
| SL249 | *S. Latissima* | 11/02/2022 | Norheimsvågen, Karmøy, Rogaland. | 59.378957 | 5.298491 | N | 0-5 | 6,7 | 34 | 38,2 | 21,4 | 50 | 3,16 | 50 | 1 |
| SL250 | *S. Latissima* | 11/02/2022 | Norheimsvågen, Karmøy, Rogaland. | 59.378957 | 5.298491 | N | 0-5 | 6,7 | 34 | 17,7 | 10,4 | 50 | 2,6 | 50 | 1 |

| | | | | | | | | | | | | | | | |
|---|---|---|---|---|---|---|---|---|---|---|---|---|---|---|---|
| SL251 | *S. Latissima* | 11/02/2022 | Norheimsvågen, Karmøy, Rogaland. | 59.378957 | 5.298491 | N | 0-5 | 6,7 | 34 | 22,5 | 21,6 | 50 | 2,32 | 50 | 1 |
| SL252 | *S. Latissima* | 11/02/2022 | Norheimsvågen, Karmøy, Rogaland. | 59.378957 | 5.298491 | N | 0-5 | 6,7 | 34 | 29 | 23,4 | 50 | 12,9 | 50 | 1 |
| SL253 | *S. Latissima* | 11/02/2022 | Norheimsvågen, Karmøy, Rogaland. | 59.378957 | 5.298491 | N | 0-5 | 6,7 | 34 | 33,6 | 19,9 | 50 | 6,76 | 50 | 1 |
| SL254 | *S. Latissima* | 11/02/2022 | Norheimsvågen, Karmøy, Rogaland. | 59.378957 | 5.298491 | N | 0-5 | 6,7 | 34 | 30,8 | 6,18 | 50 | 6,08 | 50 | 1 |
| SL255 | *S. Latissima* | 25/03/2022 | Norheimsvågen, Karmøy, Rogaland. | 59.378957 | 5.298491 | N | 0-5 | 6,8 | 32 | 23,7 | 22,6 | 50 | 7,7 | 50 | 1 |
| SL256 | *S. Latissima* | 25/03/2022 | Norheimsvågen, Karmøy, Rogaland. | 59.378957 | 5.298491 | N | 0-5 | 6,8 | 32 | 38,7 | 14,2 | 50 | 2,76 | 50 | 1 |
| SL257 | *S. Latissima* | 25/03/2022 | Norheimsvågen, Karmøy, Rogaland. | 59.378957 | 5.298491 | N | 0-5 | 6,8 | 32 | 28,3 | 33,8 | 50 | 27,8 | 50 | 1 |
| SL258 | *S. Latissima* | 25/03/2022 | Norheimsvågen, Karmøy, Rogaland. | 59.378957 | 5.298491 | N | 0-5 | 6,8 | 32 | 32,1 | 23,2 | 50 | 14,8 | 50 | 1 |
| SL259 | *S. Latissima* | 25/03/2022 | Norheimsvågen, Karmøy, Rogaland. | 59.378957 | 5.298491 | N | 0-5 | 6,8 | 32 | 35 | 4 | 50 | 1,14 | 50 | 1 |
| SL260 | *S. Latissima* | 25/03/2022 | Norheimsvågen, Karmøy, Rogaland. | 59.378957 | 5.298491 | N | 0-5 | 6,8 | 32 | 30,8 | 22,4 | 50 | 8,04 | 50 | 1 |
| SL261 | *S. Latissima* | 25/03/2022 | Norheimsvågen, Karmøy, Rogaland. | 59.378957 | 5.298491 | N | 0-5 | 6,8 | 32 | 29,8 | 13,9 | 50 | 3,82 | 50 | 0 |
| SL262 | *S. Latissima* | 25/03/2022 | Norheimsvågen, Karmøy, Rogaland. | 59.378957 | 5.298491 | N | 0-5 | 6,8 | 32 | 21,9 | 10,9 | 50 | 1,29 | 50 | 1 |
| SL263 | *S. Latissima* | 25/03/2022 | Norheimsvågen, Karmøy, Rogaland. | 59.378957 | 5.298491 | N | 0-5 | 6,8 | 32 | 31,6 | 45 | 50 | 23,6 | 50 | 1 |
| SL264 | *S. Latissima* | 25/03/2022 | Norheimsvågen, Karmøy, Rogaland. | 59.378957 | 5.298491 | N | 0-5 | 6,8 | 32 | 22,9 | 18,2 | 50 | 2,72 | 50 | 0 |

| | | | | | | | | | | | | | | | |
|---|---|---|---|---|---|---|---|---|---|---|---|---|---|---|---|
| SL265 | *S. Latissima* | 25/03/2022 | Norheimsvågen, Karmøy, Rogaland. | 59.378957 | 5.298491 | N | 0-5 | 6,8 | 32 | 26,5 | 18,1 | 50 | 8,64 | 50 | 1 |
| SL266 | *S. Latissima* | 25/03/2022 | Norheimsvågen, Karmøy, Rogaland. | 59.378957 | 5.298491 | N | 0-5 | 6,8 | 32 | 34,9 | 25 | 50 | 7,54 | 50 | 1 |
| SL267 | *S. Latissima* | 25/03/2022 | Norheimsvågen, Karmøy, Rogaland. | 59.378957 | 5.298491 | N | 0-5 | 6,8 | 32 | 28,1 | 20 | 50 | 9,54 | 50 | 0 |
| SL268 | *S. Latissima* | 25/03/2022 | Norheimsvågen, Karmøy, Rogaland. | 59.378957 | 5.298491 | N | 0-5 | 6,8 | 32 | 17,9 | 9,6 | 50 | 6,36 | 50 | 1 |
| SL269 | *S. Latissima* | 25/03/2022 | Norheimsvågen, Karmøy, Rogaland. | 59.378957 | 5.298491 | N | 0-5 | 6,8 | 32 | 24,8 | 16,6 | 50 | 16,9 | 50 | 1 |
| SL270 | *S. Latissima* | 25/03/2022 | Norheimsvågen, Karmøy, Rogaland. | 59.378957 | 5.298491 | N | 0-5 | 6,8 | 32 | 28,8 | 19,5 | 50 | 4,22 | 50 | 1 |
| SL271 | *S. Latissima* | 25/03/2022 | Norheimsvågen, Karmøy, Rogaland. | 59.378957 | 5.298491 | N | 0-5 | 6,8 | 32 | 16,2 | 12,9 | 50 | too low | 50 | 1 |
| SL272 | *S. Latissima* | 25/03/2022 | Norheimsvågen, Karmøy, Rogaland. | 59.378957 | 5.298491 | N | 0-5 | 6,8 | 32 | 19,4 | 38,8 | 50 | 12,6 | 50 | 1 |
| SL273 | *S. Latissima* | 25/03/2022 | Norheimsvågen, Karmøy, Rogaland. | 59.378957 | 5.298491 | N | 0-5 | 6,8 | 32 | 15,1 | 6,46 | 50 | 0,822 | 50 | 1 |
| SL274 | *S. Latissima* | 25/03/2022 | Norheimsvågen, Karmøy, Rogaland. | 59.378957 | 5.298491 | N | 0-5 | 6,8 | 32 | 17,8 | 8,3 | 50 | 1,19 | 50 | 1 |
| SL275 | *S. Latissima* | 25/03/2022 | Norheimsvågen, Karmøy, Rogaland. | 59.378957 | 5.298491 | N | 0-5 | 6,8 | 32 | 32,7 | 25,2 | 50 | 7,16 | 50 | 1 |
| SL276 | *S. Latissima* | 25/03/2022 | Norheimsvågen, Karmøy, Rogaland. | 59.378957 | 5.298491 | N | 0-5 | 6,8 | 32 | 11,8 | 4,98 | 50 | 0,918 | 50 | 1 |
| SL277 | *S. Latissima* | 25/03/2022 | Norheimsvågen, Karmøy, Rogaland. | 59.378957 | 5.298491 | N | 0-5 | 6,8 | 32 | 14,8 | 7,06 | 50 | 3,44 | 50 | 1 |
| SL278 | *S. Latissima* | 25/03/2022 | Norheimsvågen, Karmøy, Rogaland. | 59.378957 | 5.298491 | N | 0-5 | 6,8 | 32 | 30,5 | 15,4 | 50 | 21 | 50 | 1 |

| | | | | | | | | | | | | | | | |
|---|---|---|---|---|---|---|---|---|---|---|---|---|---|---|---|
| SL279 | *S. Latissima* | 25/03/2022 | Norheimsvågen, Karmøy, Rogaland. | 59.378957 | 5.298491 | N | 0-5 | 6,8 | 32 | 15 | 7,34 | 50 | 8,88 | 50 | 0 |
| SL280 | *S. Latissima* | 25/03/2022 | Norheimsvågen, Karmøy, Rogaland. | 59.378957 | 5.298491 | N | 0-5 | 6,8 | 32 | 24,5 | 17,9 | 50 | 5,02 | 50 | 1 |
| SL281 | *S. Latissima* | 25/03/2022 | Norheimsvågen, Karmøy, Rogaland. | 59.378957 | 5.298491 | N | 0-5 | 6,8 | 32 | 19,2 | 8,42 | 50 | 1,79 | 50 | 1 |
| SL282 | *S. Latissima* | 25/03/2022 | Norheimsvågen, Karmøy, Rogaland. | 59.378957 | 5.298491 | N | 0-5 | 6,8 | 32 | 19 | 6,18 | 50 | 4,76 | 50 | 1 |
| SL283 | *S. Latissima* | 25/03/2022 | Norheimsvågen, Karmøy, Rogaland. | 59.378957 | 5.298491 | N | 0-5 | 6,8 | 32 | 34,7 | 33,2 | 50 | 16,9 | 50 | 1 |
| SL284 | *S. Latissima* | 25/03/2022 | Norheimsvågen, Karmøy, Rogaland. | 59.378957 | 5.298491 | N | 0-5 | 6,8 | 32 | 20,5 | 8,54 | 50 | 8,96 | 50 | 1 |
| SL285 | *S. Latissima* | 25/03/2022 | Norheimsvågen, Karmøy, Rogaland. | 59.378957 | 5.298491 | N | 0-5 | 6,8 | 32 | 33,4 | 12,9 | 50 | 11,1 | 50 | 1 |
| SL286 | *S. Latissima* | 25/03/2022 | Norheimsvågen, Karmøy, Rogaland. | 59.378957 | 5.298491 | N | 0-5 | 6,8 | 32 | 10,5 | 3,36 | 50 | 0,558 | 50 | 1 |
| SL287 | *S. Latissima* | 25/03/2022 | Norheimsvågen, Karmøy, Rogaland. | 59.378957 | 5.298491 | N | 0-5 | 6,8 | 32 | 34,1 | 15,2 | 50 | 2,78 | 50 | 1 |
| SL288 | *S. Latissima* | 25/03/2022 | Norheimsvågen, Karmøy, Rogaland. | 59.378957 | 5.298491 | N | 0-5 | 6,8 | 32 | 17,2 | 6,46 | 50 | 3,18 | 50 | 0 |
| SL289 | *S. Latissima* | 25/03/2022 | Norheimsvågen, Karmøy, Rogaland. | 59.378957 | 5.298491 | N | 0-5 | 6,8 | 32 | 27,2 | 12,7 | 50 | 8,24 | 50 | 1 |
| SL290 | *S. Latissima* | 25/03/2022 | Størøy, Haugesund, Rogaland | 59.410729 | 5.234212 | S | 0-5 | 6,2 | 33 | 28,5 | 23 | 50 | 3,96 | 50 | 1 |
| SL291 | *S. Latissima* | 25/03/2022 | Størøy, Haugesund, Rogaland | 59.410729 | 5.234212 | S | 0-5 | 6,2 | 33 | 31,6 | 36,4 | 50 | 25,8 | 50 | 1 |
| SL292 | *S. Latissima* | 25/03/2022 | Størøy, Haugesund, Rogaland | 59.410729 | 5.234212 | S | 0-5 | 6,2 | 33 | 37,3 | 22,4 | 50 | 2,22 | 50 | 1 |

| | | | | | | | | | | | | | | | |
|---|---|---|---|---|---|---|---|---|---|---|---|---|---|---|---|
| SL293 | *S. Latissima* | 25/03/2022 | Størøy, Haugesund, Rogaland | 59.410729 | 5.234212 | S | 0-5 | 6,2 | 33 | 20 | 20,8 | 50 | 5,86 | 50 | 1 |
| SL294 | *S. Latissima* | 25/03/2022 | Størøy, Haugesund, Rogaland | 59.410729 | 5.234212 | S | 0-5 | 6,2 | 33 | 33,4 | 9,9 | 50 | 9,08 | 50 | 1 |
| SL295 | *S. Latissima* | 25/03/2022 | Størøy, Haugesund, Rogaland | 59.410729 | 5.234212 | S | 0-5 | 6,2 | 33 | 37,5 | 88,2 | 50 | 62,6 | 50 | 1 |
| SL296 | *S. Latissima* | 25/03/2022 | Størøy, Haugesund, Rogaland | 59.410729 | 5.234212 | S | 0-5 | 6,2 | 33 | 28,2 | 15 | 50 | 2,1 | 50 | 1 |
| SL297 | *S. Latissima* | 25/03/2022 | Størøy, Haugesund, Rogaland | 59.410729 | 5.234212 | S | 0-5 | 6,2 | 33 | 32,5 | 16,4 | 50 | 4 | 50 | 1 |
| SL298 | *S. Latissima* | 25/03/2022 | Størøy, Haugesund, Rogaland | 59.410729 | 5.234212 | S | 0-5 | 6,2 | 33 | 31,7 | 5,54 | 50 | 0,984 | 50 | 1 |
| SL299 | *S. Latissima* | 25/03/2022 | Størøy, Haugesund, Rogaland | 59.410729 | 5.234212 | S | 0-5 | 6,2 | 33 | 19,9 | 7,16 | 50 | 1,65 | 50 | 1 |
| SL300 | *S. Latissima* | 25/03/2022 | Størøy, Haugesund, Rogaland | 59.410729 | 5.234212 | S | 0-5 | 6,2 | 33 | 22,4 | 20,8 | 50 | 23,6 | 50 | 1 |
| SL301 | *S. Latissima* | 25/03/2022 | Størøy, Haugesund, Rogaland | 59.410729 | 5.234212 | S | 0-5 | 6,2 | 33 | 32,8 | 9,02 | 50 | 9,96 | 50 | 1 |
| SL302 | *S. Latissima* | 25/03/2022 | Størøy, Haugesund, Rogaland | 59.410729 | 5.234212 | S | 0-5 | 6,2 | 33 | 24,1 | 28,6 | 50 | 19,3 | 50 | 1 |
| SL303 | *S. Latissima* | 25/03/2022 | Størøy, Haugesund, Rogaland | 59.410729 | 5.234212 | S | 0-5 | 6,2 | 33 | 24,2 | 27,4 | 50 | 15 | 50 | 1 |
| SL304 | *S. Latissima* | 25/03/2022 | Størøy, Haugesund, Rogaland | 59.410729 | 5.234212 | S | 0-5 | 6,2 | 33 | 28,3 | 23,8 | 50 | 8,2 | 50 | 1 |
| SL305 | *S. Latissima* | 25/03/2022 | Størøy, Haugesund, Rogaland | 59.410729 | 5.234212 | S | 0-5 | 6,2 | 33 | 36,4 | 9,36 | 50 | 13,8 | 50 | 1 |
| SL306 | *S. Latissima* | 25/03/2022 | Størøy, Haugesund, Rogaland | 59.410729 | 5.234212 | S | 0-5 | 6,2 | 33 | 24,8 | 43,6 | 50 | 22,2 | 50 | 1 |

| | | | | | | | | | | | | | | | |
|---|---|---|---|---|---|---|---|---|---|---|---|---|---|---|---|
| SL307 | *S. Latissima* | 25/03/2022 | Størøy, Haugesund, Rogaland | 59.410729 | 5.234212 | S | 0-5 | 6,2 | 33 | 26,3 | 32,2 | 50 | 15,1 | 50 | 1 |
| SL308 | *S. Latissima* | 25/03/2022 | Størøy, Haugesund, Rogaland | 59.410729 | 5.234212 | S | 0-5 | 6,2 | 33 | 35 | 13,9 | 50 | 5,74 | 50 | 1 |
| SL309 | *S. Latissima* | 25/03/2022 | Størøy, Haugesund, Rogaland | 59.410729 | 5.234212 | S | 0-5 | 6,2 | 33 | 26,7 | 15,7 | 50 | 11,4 | 50 | 1 |
| SL310 | *S. Latissima* | 25/03/2022 | Størøy, Haugesund, Rogaland | 59.410729 | 5.234212 | S | 0-5 | 6,2 | 33 | 23,2 | 51,6 | 50 | 26,8 | 50 | 1 |
| SL311 | *S. Latissima* | 25/03/2022 | Størøy, Haugesund, Rogaland | 59.410729 | 5.234212 | S | 0-5 | 6,2 | 33 | 33,2 | 23,6 | 50 | 42,2 | 50 | 1 |
| SL312 | *S. Latissima* | 25/03/2022 | Størøy, Haugesund, Rogaland | 59.410729 | 5.234212 | S | 0-5 | 6,2 | 33 | 24,1 | 8,88 | 50 | 8,28 | 50 | 1 |
| SL313 | *S. Latissima* | 25/03/2022 | Størøy, Haugesund, Rogaland | 59.410729 | 5.234212 | S | 0-5 | 6,2 | 33 | 21,9 | 9,1 | 50 | 5,16 | 50 | 1 |
| SL314 | *S. Latissima* | 25/03/2022 | Størøy, Haugesund, Rogaland | 59.410729 | 5.234212 | S | 0-5 | 6,2 | 33 | 16,1 | 11 | 50 | 9,72 | 50 | 1 |
| SL315 | *S. Latissima* | 25/03/2022 | Størøy, Haugesund, Rogaland | 59.410729 | 5.234212 | S | 0-5 | 6,2 | 33 | 22,2 | 8,82 | 50 | 2,14 | 50 | 1 |
| SL316 | *S. Latissima* | 25/03/2022 | Størøy, Haugesund, Rogaland | 59.410729 | 5.234212 | S | 0-5 | 6,2 | 33 | 26 | 8,12 | 50 | 9,34 | 50 | 1 |
| SL317 | *S. Latissima* | 25/03/2022 | Størøy, Haugesund, Rogaland | 59.410729 | 5.234212 | S | 0-5 | 6,2 | 33 | 22,1 | 27,8 | 50 | 23,2 | 50 | 1 |
| SL318 | *S. Latissima* | 25/03/2022 | Størøy, Haugesund, Rogaland | 59.410729 | 5.234212 | S | 0-5 | 6,2 | 33 | 23,4 | 11,8 | 50 | 7,1 | 50 | 1 |
| SL319 | *S. Latissima* | 25/03/2022 | Størøy, Haugesund, Rogaland | 59.410729 | 5.234212 | S | 0-5 | 6,2 | 33 | 14,7 | 8,14 | 50 | 4,58 | 50 | 1 |
| SL320 | *S. Latissima* | 25/03/2022 | Størøy, Haugesund, Rogaland | 59.410729 | 5.234212 | S | 0-5 | 6,2 | 33 | 22,6 | 10,3 | 50 | 10,7 | 50 | 1 |

| | | | | | | | | | | | | | | | |
|---|---|---|---|---|---|---|---|---|---|---|---|---|---|---|---|
| SL321 | *S. Latissima* | 25/03/2022 | Stør øy, Haugesund, Rogaland | 59.410729 | 5.234212 | S | 0-5 | 6,2 | 33 | 13,1 | 12,3 | 50 | 8,8 | 50 | 1 |
| SL322 | *S. Latissima* | 25/03/2022 | Størøy, Haugesund, Rogaland | 59.410729 | 5.234212 | S | 0-5 | 6,2 | 33 | 35,3 | 12,5 | 50 | 15,1 | 50 | 1 |
| SL323 | *S. Latissima* | 25/03/2022 | Størøy, Haugesund, Rogaland | 59.410729 | 5.234212 | S | 0-5 | 6,2 | 33 | 19,9 | 10,9 | 50 | 2,54 | 50 | 1 |
| SL324 | *S. Latissima* | 25/03/2022 | Størøy, Haugesund, Rogaland | 59.410729 | 5.234212 | S | 0-5 | 6,2 | 33 | 32 | 12,6 | 50 | 4,42 | 50 | 1 |
| SL325 | *S. Latissima* | 25/03/2022 | Størøy, Haugesund, Rogaland | 59.410729 | 5.234212 | S | 0-5 | 6,2 | 33 | 20 | 4,58 | 50 | 4,46 | 50 | 1 |
| SL326 | *S. Latissima* | 25/03/2022 | Størøy, Haugesund, Rogaland | 59.410729 | 5.234212 | S | 0-5 | 6,2 | 33 | 25,1 | 18,1 | 50 | 5,24 | 50 | 1 |
| SL327 | *S. Latissima* | 25/03/2022 | Størøy, Haugesund, Rogaland | 59.410729 | 5.234212 | S | 0-5 | 6,2 | 33 | 33,5 | 33,4 | 50 | 5,46 | 50 | 1 |
| SL328 | *S. Latissima* | 25/03/2022 | Størøy, Haugesund, Rogaland | 59.410729 | 5.234212 | S | 0-5 | 6,2 | 33 | 25,7 | 13,9 | 50 | 5,08 | 50 | 1 |
| SL329 | *S. Latissima* | 25/03/2022 | Størøy, Haugesund, Rogaland | 59.410729 | 5.234212 | S | 0-5 | 6,2 | 33 | 17,1 | 3,88 | 50 | 3,64 | 50 | 1 |
| SL330 | *S. Latissima* | 15/06/2022 | Korsfjorden, Bergen, Hordaland | 60.240977 | 5.240332 | K-2 | 0-5 | 13,6 | 31 | 27,4 | 12,8 | 50 | 8,68 | 50 | 1 |
| SL331 | *S. Latissima* | 15/06/2022 | Korsfjorden, Bergen, Hordaland | 60.240977 | 5.240332 | K-2 | 0-5 | 13,6 | 31 | 24,4 | 16,3 | 50 | 15,8 | 50 | 1 |
| SL332 | *S. Latissima* | 15/06/2022 | Korsfjorden, Bergen, Hordaland | 60.240977 | 5.240332 | K-2 | 0-5 | 13,6 | 31 | 15,9 | 3,4 | 50 | 3,38 | 50 | 1 |
| SL333 | *S. Latissima* | 15/06/2022 | Korsfjorden, Bergen, Hordaland | 60.240977 | 5.240332 | K-2 | 0-5 | 13,6 | 31 | 33,3 | 15,8 | 50 | 15,5 | 50 | 1 |
| SL334 | *S. Latissima* | 15/06/2022 | Korsfjorden, Bergen, Hordaland | 60.240977 | 5.240332 | K-2 | 0-5 | 13,6 | 31 | 30,4 | 24,4 | 50 | 5,14 | 50 | 1 |

| | | | | | | | | | | | | | | | |
|---|---|---|---|---|---|---|---|---|---|---|---|---|---|---|---|
| SL335 | *S. Latissima* | 15/06/2022 | Korsfjorden, Bergen, Hordaland | 60.240977 | 5.240332 | K-2 | 0-5 | 13,6 | 31 | 15,5 | 3,74 | 50 | 3,46 | 50 | 1 |
| SL336 | *S. Latissima* | 15/06/2022 | Korsfjorden, Bergen, Hordaland | 60.240977 | 5.240332 | K-2 | 0-5 | 13,6 | 31 | 23,1 | 22,2 | 50 | 10,7 | 50 | 1 |
| SL337 | *S. Latissima* | 15/06/2022 | Korsfjorden, Bergen, Hordaland | 60.240977 | 5.240332 | K-2 | 0-5 | 13,6 | 31 | 20,5 | 8,84 | 50 | 8,84 | 50 | 1 |
| SL338 | *S. Latissima* | 15/06/2022 | Korsfjorden, Bergen, Hordaland | 60.240977 | 5.240332 | K-2 | 0-5 | 13,6 | 31 | 20,6 | 7,74 | 50 | 1,94 | 50 | 1 |
| SL339 | *S. Latissima* | 15/06/2022 | Korsfjorden, Bergen, Hordaland | 60.240977 | 5.240332 | K-2 | 0-5 | 13,6 | 31 | 33,3 | 22,8 | 50 | 14,5 | 50 | 1 |
| SL340 | *S. Latissima* | 15/06/2022 | Korsfjorden, Bergen, Hordaland | 60.240977 | 5.240332 | K-2 | 0-5 | 13,6 | 31 | 24,5 | 23,4 | 50 | 0,146 | 50 | 1 |
| SL341 | *S. Latissima* | 15/06/2022 | Korsfjorden, Bergen, Hordaland | 60.240977 | 5.240332 | K-2 | 0-5 | 13,6 | 31 | 16,5 | 1,75 | 50 | 3,26 | 50 | 1 |
| SL342 | *S. Latissima* | 15/06/2022 | Korsfjorden, Bergen, Hordaland | 60.240977 | 5.240332 | K-2 | 0-5 | 13,6 | 31 | 33,8 | 7,92 | 50 | 10,3 | 50 | 1 |
| SL343 | *S. Latissima* | 15/06/2022 | Korsfjorden, Bergen, Hordaland | 60.240977 | 5.240332 | K-2 | 0-5 | 13,6 | 31 | 24,7 | 8,14 | 50 | 8,6 | 50 | 1 |
| SL344 | *S. Latissima* | 15/06/2022 | Korsfjorden, Bergen, Hordaland | 60.240977 | 5.240332 | K-2 | 0-5 | 13,6 | 31 | 27,4 | 15,3 | 50 | 11,8 | 50 | 1 |
| SL345 | *S. Latissima* | 15/06/2022 | Korsfjorden, Bergen, Hordaland | 60.240977 | 5.240332 | K-2 | 0-5 | 13,6 | 31 | 22,1 | 7,52 | 50 | 5,36 | 50 | 1 |
| SL346 | *S. Latissima* | 15/06/2022 | Korsfjorden, Bergen, Hordaland | 60.240977 | 5.240332 | K-2 | 0-5 | 13,6 | 31 | 18,9 | 3,22 | 50 | 2,84 | 50 | 1 |
| SL347 | *S. Latissima* | 15/06/2022 | Korsfjorden, Bergen, Hordaland | 60.240977 | 5.240332 | K-2 | 0-5 | 13,6 | 31 | 28,5 | 26 | 50 | 21 | 50 | 1 |
| SL348 | *S. Latissima* | 15/06/2022 | Korsfjorden, Bergen, Hordaland | 60.240977 | 5.240332 | K-2 | 0-5 | 13,6 | 31 | 23,9 | 6,84 | 50 | 6,7 | 50 | 1 |

| | | | | | | | | | | | | | | | |
|---|---|---|---|---|---|---|---|---|---|---|---|---|---|---|---|
| SL349 | *S. Latissima* | 15/06/2022 | Korsfjorden, Bergen, Hordaland | 60.240977 | 5.240332 | K-2 | 0-5 | 13,6 | 31 | 15,9 | 7,34 | 50 | 7,38 | 50 | 1 |
| SL350 | *S. Latissima* | 15/06/2022 | Korsfjorden, Bergen, Hordaland | 60.240977 | 5.240332 | K-2 | 0-5 | 13,6 | 31 | 23 | 8,88 | 50 | 7,66 | 50 | 1 |
| SL351 | *S. Latissima* | 15/06/2022 | Korsfjorden, Bergen, Hordaland | 60.240977 | 5.240332 | K-2 | 0-5 | 13,6 | 31 | 28,9 | 6,28 | 50 | 1,69 | 50 | 1 |
| SL352 | *S. Latissima* | 15/06/2022 | Korsfjorden, Bergen, Hordaland | 60.240977 | 5.240332 | K-2 | 0-5 | 13,6 | 31 | 27,7 | 5,7 | 50 | 5,18 | 50 | 1 |
| SL353 | *S. Latissima* | 26/10/2022 | Korsfjorden, Bergen, Hordaland | 60.240977 | 5.240332 | K-2 | 0-5 | 10,8 | 31 | 23,2 | 6,96 | 50 | 12,6 | 50 | 1 |
| SL354 | *S. Latissima* | 26/10/2022 | Korsfjorden, Bergen, Hordaland | 60.240977 | 5.240332 | K-2 | 0-5 | 10,8 | 31 | 19,2 | 8,3 | 50 | 4,44 | 50 | 1 |
| SL355 | *S. Latissima* | 26/10/2022 | Korsfjorden, Bergen, Hordaland | 60.240977 | 5.240332 | K-2 | 0-5 | 10,8 | 31 | 21,5 | 5,48 | 50 | 11 | 50 | 1 |
| SL356 | *S. Latissima* | 26/10/2022 | Korsfjorden, Bergen, Hordaland | 60.240977 | 5.240332 | K-2 | 0-5 | 10,8 | 31 | 26,2 | 14,4 | 50 | 18,7 | 50 | 1 |
| SL357 | *S. Latissima* | 26/10/2022 | Korsfjorden, Bergen, Hordaland | 60.240977 | 5.240332 | K-2 | 0-5 | 10,8 | 31 | 21,7 | 4,22 | 50 | 2,7 | 50 | 1 |
| SL358 | *S. Latissima* | 26/10/2022 | Korsfjorden, Bergen, Hordaland | 60.240977 | 5.240332 | K-2 | 0-5 | 10,8 | 31 | 12,5 | 2,8 | 50 | 2,36 | 50 | 1 |
| SL359 | *S. Latissima* | 26/10/2022 | Korsfjorden, Bergen, Hordaland | 60.240977 | 5.240332 | K-2 | 0-5 | 10,8 | 31 | 26 | 4,22 | 50 | 4 | 50 | 1 |
| SL360 | *S. Latissima* | 26/10/2022 | Korsfjorden, Bergen, Hordaland | 60.240977 | 5.240332 | K-2 | 0-5 | 10,8 | 31 | 17,4 | 7,7 | 50 | 3,26 | 50 | 1 |
| SL361 | *S. Latissima* | 26/10/2022 | Korsfjorden, Bergen, Hordaland | 60.240977 | 5.240332 | K-2 | 0-5 | 10,8 | 31 | 25,1 | 21,2 | 50 | 23,4 | 50 | 1 |
| SL362 | *S. Latissima* | 26/10/2022 | Korsfjorden, Bergen, Hordaland | 60.240977 | 5.240332 | K-2 | 0-5 | 10,8 | 31 | 21,3 | 3,06 | 50 | 2,68 | 50 | 0 |

| | | | | | | | | | | | | | | | |
|---|---|---|---|---|---|---|---|---|---|---|---|---|---|---|---|
| SL363 | *S. Latissima* | 26/10/2022 | Korsfjorden, Bergen, Hordaland | 60.240977 | 5.240332 | K-2 | 0-5 | 10,8 | 31 | 18,6 | 3,36 | 50 | 2,84 | 50 | 1 |
| SL364 | *S. Latissima* | 26/10/2022 | Korsfjorden, Bergen, Hordaland | 60.240977 | 5.240332 | K-2 | 0-5 | 10,8 | 31 | 16 | 9,68 | 50 | 2 | 50 | 1 |
| SL365 | *S. Latissima* | 26/10/2022 | Korsfjorden, Bergen, Hordaland | 60.240977 | 5.240332 | K-2 | 0-5 | 10,8 | 31 | 17,6 | 12,9 | 50 | 5,14 | 50 | 1 |
| SL366 | *S. Latissima* | 26/10/2022 | Korsfjorden, Bergen, Hordaland | 60.240977 | 5.240332 | K-2 | 0-5 | 10,8 | 31 | 18,7 | 3,92 | 50 | 5,16 | 50 | 1 |
| SL367 | *S. Latissima* | 26/10/2022 | Korsfjorden, Bergen, Hordaland | 60.240977 | 5.240332 | K-2 | 0-5 | 10,8 | 31 | 28,5 | 8,74 | 50 | 4,82 | 50 | 0 |
| SL368 | *S. Latissima* | 26/10/2022 | Korsfjorden, Bergen, Hordaland | 60.240977 | 5.240332 | K-2 | 0-5 | 10,8 | 31 | 24,5 | 9,76 | 50 | 5,3 | 50 | 1 |
| SL369 | *S. Latissima* | 26/10/2022 | Korsfjorden, Bergen, Hordaland | 60.240977 | 5.240332 | K-2 | 0-5 | 10,8 | 31 | 29,7 | 6,86 | 50 | 4,84 | 50 | 1 |
| SL370 | *S. Latissima* | 26/10/2022 | Korsfjorden, Bergen, Hordaland | 60.240977 | 5.240332 | K-2 | 0-5 | 10,8 | 31 | 29,7 | 13,4 | 50 | 12,9 | 50 | 1 |
| SL371 | *S. Latissima* | 26/10/2022 | Korsfjorden, Bergen, Hordaland | 60.240977 | 5.240332 | K-2 | 0-5 | 10,8 | 31 | 28,5 | 12,3 | 50 | 7,96 | 50 | 1 |
| SL372 | *S. Latissima* | 26/10/2022 | Korsfjorden, Bergen, Hordaland | 60.240977 | 5.240332 | K-2 | 0-5 | 10,8 | 31 | 18 | 5,22 | 50 | too low | 50 | 1 |

**Table S2:** ***L.hyperborea*** **samples (2021-2022).**

| Sample | Species | Date | Location | Lat | Long | Sampling station | Depth (m) | AV Ta (Celsius) | Salinity | Dry weight (mg) | DNA 1 (ng/ul) | Volume (ul) | DNA 2 (ng/ul) | Volume (ul) | Virus positive? |
|---|---|---|---|---|---|---|---|---|---|---|---|---|---|---|---|
| LH1 | *L.hyperborea* | 17/06/2021 | Vikna, Nord-Trondelag | 64.054167 | 8.599167 | V | 5-10 | 10,3 | 34 | 23 | 4,28 | 50 | 1,02 | 50 | 1 |
| LH2 | *L.hyperborea* | 17/06/2021 | Vikna, Nord-Trondelag | 64.054167 | 8.599167 | V | 5-10 | 10,3 | 34 | 17 | 5,36 | 50 | 1,77 | 50 | 0 |
| LH3 | *L.hyperborea* | 17/06/2021 | Vikna, Nord-Trondelag | 64.054167 | 8.599167 | V | 5-10 | 10,3 | 34 | 15 | 6,3 | 50 | 1,89 | 50 | 1 |
| LH4 | *L.hyperborea* | 17/06/2021 | Vikna, Nord-Trondelag | 64.054167 | 8.599167 | V | 5-10 | 10,3 | 34 | 23 | 8,72 | 50 | 1,91 | 50 | 1 |
| LH5 | *L.hyperborea* | 17/06/2021 | Vikna, Nord-Trondelag | 64.054167 | 8.599167 | V | 5-10 | 10,3 | 34 | 18 | 8,2 | 50 | 2,8 | 50 | 1 |
| LH6 | *L.hyperborea* | 17/06/2021 | Vikna, Nord-Trondelag | 64.054167 | 8.599167 | V | 5-10 | 10,3 | 34 | 43 | 2,66 | 50 | 0,472 | 50 | 1 |
| LH7 | *L.hyperborea* | 17/06/2021 | Vikna, Nord-Trondelag | 64.054167 | 8.599167 | V | 5-10 | 10,3 | 34 | 20 | 13,8 | 50 | 2,9 | 50 | 1 |
| LH8 | *L.hyperborea* | 17/06/2021 | Vikna, Nord-Trondelag | 64.054167 | 8.599167 | V | 5-10 | 10,3 | 34 | 23 | 4,02 | 50 | 0,354 | 50 | 1 |
| LH9 | *L.hyperborea* | 17/06/2021 | Vikna, Nord-Trondelag | 64.054167 | 8.599167 | V | 5-10 | 10,3 | 34 | 21 | 11,3 | 50 | 0,354 | 50 | 1 |
| LH10 | *L.hyperborea* | 17/06/2021 | Vikna, Nord-Trondelag | 64.054167 | 8.599167 | V | 5-10 | 10,3 | 34 | 21 | 4,48 | 50 | 1,4 | 50 | 1 |
| LH11 | *L.hyperborea* | 17/06/2021 | Vikna, Nord-Trondelag | 64.054167 | 8.599167 | V | 5-10 | 10,3 | 34 | 21 | 5,48 | 50 | 0,734 | 50 | 1 |
| LH12 | *L.hyperborea* | 17/06/2021 | Vikna, Nord-Trondelag | 64.054167 | 8.599167 | V | 5-10 | 10,3 | 34 | 18 | 11,2 | 50 | 2,42 | 50 | 1 |
| LH13 | *L.hyperborea* | 17/06/2021 | Vikna, Nord-Trondelag | 64.054167 | 8.599167 | V | 5-10 | 10,3 | 34 | 21 | 7,52 | 50 | 2,18 | 50 | 1 |
| LH14 | *L.hyperborea* | 16/06/2021 | Vikna, Nord-Trondelag | 64.054167 | 8.599167 | V | 5-10 | 10,3 | 34 | 19 | 14,5 | 50 | 3,2 | 50 | 1 |
| LH15 | *L.hyperborea* | 16/06/2021 | Vikna, Nord-Trondelag | 64.054167 | 8.599167 | V | 5-10 | 10,3 | 34 | 23 | 17,1 | 50 | 1,31 | 50 | 1 |
| LH16 | *L.hyperborea* | 16/06/2021 | Vikna, Nord-Trondelag | 64.054167 | 8.599167 | V | 5-10 | 10,3 | 34 | 18 | 8,1 | 50 | 2,68 | 50 | 1 |
| LH17 | *L.hyperborea* | 16/06/2021 | Vikna, Nord-Trondelag | 64.054167 | 8.599167 | V | 5-10 | 10,3 | 34 | 19 | 15,2 | 50 | 3,26 | 50 | 1 |
| LH18 | *L.hyperborea* | 16/06/2021 | Vikna, Nord-Trondelag | 64.054167 | 8.599167 | V | 5-10 | 10,3 | 34 | 20 | 6,64 | 50 | 1,85 | 50 | 1 |
| LH19 | *L.hyperborea* | 16/06/2021 | Vikna, Nord-Trondelag | 64.054167 | 8.599167 | V | 5-10 | 10,3 | 34 | 25 | 9,12 | 50 | 3,36 | 50 | 1 |

| | | | | | | | | | | | | | | | |
|---|---|---|---|---|---|---|---|---|---|---|---|---|---|---|---|
| LH20 | *L.hyperborea* | 16/06/2021 | Vikna, Nord-Trondelag | 64.054167 | 8.599167 | V | 5-10 | 10,3 | 34 | 25 | 16,8 | 50 | 3,7 | 50 | 1 |
| LH21 | *L.hyperborea* | 16/06/2021 | Vikna, Nord-Trondelag | 64.054167 | 8.599167 | V | 5-10 | 10,3 | 34 | 15 | 8,94 | 50 | 2,68 | 50 | 1 |
| LH22 | *L.hyperborea* | 16/06/2021 | Vikna, Nord-Trondelag | 64.054167 | 8.599167 | V | 5-10 | 10,3 | 34 | 24 | 16,3 | 50 | 3,96 | 50 | 1 |
| LH23 | *L.hyperborea* | 16/06/2021 | Vikna, Nord-Trondelag | 64.054167 | 8.599167 | V | 5-10 | 10,3 | 34 | 15 | 14,4 | 50 | 2,7 | 50 | 1 |
| LH24 | *L.hyperborea* | 16/06/2021 | Vikna, Nord-Trondelag | 64.054167 | 8.599167 | V | 5-10 | 10,3 | 34 | 18 | 5,48 | 50 | 2,42 | 50 | 1 |
| LH25 | *L.hyperborea* | 16/06/2021 | Vikna, Nord-Trondelag | 64.054167 | 8.599167 | V | 5-10 | 10,3 | 34 | 15 | 3,52 | 50 | 1,62 | 50 | 1 |
| LH26 | *L.hyperborea* | 16/06/2021 | Vikna, Nord-Trondelag | 64.054167 | 8.599167 | V | 5-10 | 10,3 | 34 | 25 | 9,32 | 50 | 2,14 | 50 | 1 |
| LH27 | *L.hyperborea* | 16/06/2021 | Vikna, Nord-Trondelag | 64.054167 | 8.599167 | V | 5-10 | 10,3 | 34 | 22 | 6,82 | 50 | 2,18 | 50 | 1 |
| LH28 | *L.hyperborea* | 16/06/2021 | Vikna, Nord-Trondelag | 64.054167 | 8.599167 | V | 5-10 | 10,3 | 34 | 28 | 14,4 | 50 | 4,24 | 50 | 1 |
| LH29 | *L.hyperborea* | 16/06/2021 | Vikna, Nord-Trondelag | 64.054167 | 8.599167 | V | 5-10 | 10,3 | 34 | 19 | 14,3 | 50 | 2,64 | 50 | 1 |
| LH30 | *L.hyperborea* | 16/06/2021 | Vikna, Nord-Trondelag | 64.054167 | 8.599167 | V | 5-10 | 10,3 | 34 | 18 | 9,58 | 50 | 2,38 | 50 | 1 |
| LH31 | *L.hyperborea* | 16/06/2021 | Vikna, Nord-Trondelag | 64.054167 | 8.599167 | V | 5-10 | 10,3 | 34 | 15 | 9,68 | 50 | 2,44 | 50 | 0 |
| LH32 | *L.hyperborea* | 16/06/2021 | Vikna, Nord-Trondelag | 64.054167 | 8.599167 | V | 5-10 | 10,3 | 34 | 27 | 9,56 | 50 | 1,09 | 50 | 1 |
| LH33 | *L.hyperborea* | 16/06/2021 | Vikna, Nord-Trondelag | 64.054167 | 8.599167 | V | 5-10 | 10,3 | 34 | 26 | 14,1 | 50 | 2,28 | 50 | 1 |
| LH34 | *L.hyperborea* | 16/06/2021 | Vikna, Nord-Trondelag | 64.054167 | 8.599167 | V | 5-10 | 10,3 | 34 | 21 | 9,22 | 50 | 1,95 | 50 | 1 |
| LH35 | *L.hyperborea* | 16/06/2021 | Vikna, Nord-Trondelag | 64.054167 | 8.599167 | V | 5-10 | 10,3 | 34 | 20 | 11,7 | 50 | 1,91 | 50 | 1 |
| LH36 | *L.hyperborea* | 16/06/2021 | Vikna, Nord-Trondelag | 64.054167 | 8.599167 | V | 5-10 | 10,3 | 34 | 22 | 15,6 | 50 | 2,04 | 50 | 1 |
| LH37 | *L.hyperborea* | 16/06/2021 | Vikna, Nord-Trondelag | 64.054167 | 8.599167 | V | 5-10 | 10,3 | 34 | 18 | 9,62 | 50 | 9,62 | 50 | 1 |
| LH38 | *L.hyperborea* | 16/06/2021 | Vikna, Nord-Trondelag | 64.054167 | 8.599167 | V | 5-10 | 10,3 | 34 | 17 | 13,3 | 50 | 13,3 | 50 | 1 |
| LH39 | *L.hyperborea* | 27/07/2021 | Bona Sea Rogaland | 59.156389 | 5.495556 | B | 5-10 | 15,2 | 32 | 62 | 7,14 | 50 | 1,99 | 50 | 1 |
| LH40 | *L.hyperborea* | 27/07/2021 | Bona Sea Rogaland | 59.156389 | 5.495556 | B | 5-10 | 15,2 | 32 | 34 | 6,54 | 50 | 2 | 50 | 1 |

| | | | | | | | | | | | | | | | |
|---|---|---|---|---|---|---|---|---|---|---|---|---|---|---|---|
| LH41 | *L.hyperbo rea* | 27/07/2 021 | Bona Sea Rogaland | 59.156 389 | 5.4955 56 | B | 5-10 | 15,2 | 32 | 31 | 8,1 | 50 | 1,67 | 50 | 1 |
| LH42 | *L.hyperbo rea* | 27/07/2 021 | Bona Sea Rogaland | 59.156 389 | 5.4955 56 | B | 5-10 | 15,2 | 32 | 40 | 8,44 | 50 | 2,58 | 50 | 1 |
| LH43 | *L.hyperbo rea* | 27/07/2 021 | Bona Sea Rogaland | 59.156 389 | 5.4955 56 | B | 5-10 | 15,2 | 32 | 38 | 1,63 | 50 | 1,91 | 50 | 1 |
| LH44 | *L.hyperbo rea* | 27/07/2 021 | Bona Sea Rogaland | 59.156 389 | 5.4955 56 | B | 5-10 | 15,2 | 32 | 24 | 4,12 | 50 | 1,7 | 50 | 1 |
| LH45 | *L.hyperbo rea* | 27/07/2 021 | Bona Sea Rogaland | 59.156 389 | 5.4955 56 | B | 5-10 | 15,2 | 32 | 70 | 7,42 | 50 | | 50 | 1 |
| LH46 | *L.hyperbo rea* | 27/07/2 021 | Bona Sea Rogaland | 59.156 389 | 5.4955 56 | B | 5-10 | 15,2 | 32 | 20 | 3,42 | 50 | 1,48 | 50 | 0 |
| LH47 | *L.hyperbo rea* | 27/07/2 021 | Bona Sea Rogaland | 59.156 389 | 5.4955 56 | B | 5-10 | 15,2 | 32 | 28 | 7,3 | 50 | 4,5 | 50 | 1 |
| LH48 | *L.hyperbo rea* | 27/07/2 021 | Bona Sea Rogaland | 59.156 389 | 5.4955 56 | B | 5-10 | 15,2 | 32 | 28 | 4,78 | 50 | 0,954 | 50 | 1 |
| LH49 | *L.hyperbo rea* | 27/07/2 021 | Bona Sea Rogaland | 59.156 389 | 5.4955 56 | B | 5-10 | 15,2 | 32 | 25 | 5,7 | 50 | 2 | 50 | 1 |
| LH50 | *L.hyperbo rea* | 27/07/2 021 | Bona Sea Rogaland | 59.156 389 | 5.4955 56 | B | 5-10 | 15,2 | 32 | 45 | 13 | 50 | 3,02 | 50 | 0 |
| LH51 | *L.hyperbo rea* | 27/07/2 021 | Bona Sea Rogaland | 59.156 389 | 5.4955 56 | B | 5-10 | 15,2 | 32 | 27 | 4,66 | 50 | 1,03 | 50 | 1 |
| LH52 | *L.hyperbo rea* | 27/07/2 021 | Bona Sea Rogaland | 59.156 389 | 5.4955 56 | B | 5-10 | 15,2 | 32 | 25 | 4,14 | 50 | 1,76 | 50 | 1 |
| LH53 | *L.hyperbo rea* | 27/07/2 021 | Bona Sea Rogaland | 59.156 389 | 5.4955 56 | B | 5-10 | 15,2 | 32 | 26 | 2,5 | 50 | 1,4 | 50 | 1 |
| LH54 | *L.hyperbo rea* | 27/07/2 021 | Bona Sea Rogaland | 59.156 389 | 5.4955 56 | B | 5-10 | 15,2 | 32 | 53 | 4,44 | 50 | 4,22 | 50 | 1 |
| LH55 | *L.hyperbo rea* | 27/07/2 021 | Bona Sea Rogaland | 59.156 389 | 5.4955 56 | B | 5-10 | 15,2 | 32 | 19,7 | 19,7 | 50 | 2,02 | 50 | 1 |
| LH56 | *L.hyperbo rea* | 27/07/2 021 | Bona Sea Rogaland | 59.156 389 | 5.4955 56 | B | 5-10 | 15,2 | 32 | 45,5 | 10,4 | 50 | 1,75 | 50 | 1 |
| LH57 | *L.hyperbo rea* | 27/07/2 021 | Bona Sea Rogaland | 59.156 389 | 5.4955 56 | B | 5-10 | 15,2 | 32 | 54 | 4,26 | 50 | 0,984 | 50 | 1 |
| LH58 | *L.hyperbo rea* | 27/07/2 021 | Bona Sea Rogaland | 59.156 389 | 5.4955 56 | B | 5-10 | 15,2 | 32 | 23,1 | 8,24 | 50 | 3,02 | 50 | 1 |
| LH59 | *L.hyperbo rea* | 27/07/2 021 | Bona Sea Rogaland | 59.156 389 | 5.4955 56 | B | 5-10 | 15,2 | 32 | 61 | 7,54 | 50 | 0,91 | 50 | 1 |
| LH60 | *L.hyperbo rea* | 27/07/2 021 | Bona Sea Rogaland | 59.156 389 | 5.4955 56 | B | 5-10 | 15,2 | 32 | 40 | 15,2 | 50 | 2,98 | 50 | 1 |
| LH61 | *L.hyperbo rea* | 27/07/2 021 | Bona Sea Rogaland | 59.156 389 | 5.4955 56 | B | 5-10 | 15,2 | 32 | 32 | 10 | 50 | 2,52 | 50 | 1 |

| | | | | | | | | | | | | | | | |
|---|---|---|---|---|---|---|---|---|---|---|---|---|---|---|---|
| LH62 | *L.hyperborea* | 27/07/2021 | Bona Sea Rogaland | 59.156389 | 5.495556 | B | 5-10 | 15,2 | 32 | 26 | 9,8 | 50 | 4,22 | 50 | 1 |
| LH63 | *L.hyperborea* | 27/07/2021 | Bona Sea Rogaland | 59.156389 | 5.495556 | B | 5-10 | 15,2 | 32 | 14 | 7,76 | 50 | 1,68 | 50 | 1 |
| LH64 | *L.hyperborea* | 27/07/2021 | Bona Sea Rogaland | 59.156389 | 5.495556 | B | 5-10 | 15,2 | 32 | 16,6 | 8,42 | 50 | 3,1 | 50 | 1 |
| LH65 | *L.hyperborea* | 27/07/2021 | Bona Sea Rogaland | 59.156389 | 5.495556 | B | 5-10 | 15,2 | 32 | 24 | 9,2 | 50 | | 50 | 1 |
| LH66 | *L.hyperborea* | 27/07/2021 | Bona Sea Rogaland | 59.156389 | 5.495556 | B | 5-10 | 15,2 | 32 | 24 | 10,4 | 50 | | 50 | 1 |
| LH67 | *L.hyperborea* | 27/07/2021 | Bona Sea Rogaland | 59.156389 | 5.495556 | B | 5-10 | 15,2 | 32 | 28 | 11,6 | 50 | | 50 | 1 |
| LH68 | *L.hyperborea* | 27/07/2021 | Bona Sea Rogaland | 59.156389 | 5.495556 | B | 5-10 | 15,2 | 32 | 29 | 7,84 | 50 | | 50 | 1 |
| LH69 | *L.hyperborea* | 27/07/2021 | Bona Sea Rogaland | 59.156389 | 5.495556 | B | 5-10 | 15,2 | 32 | 30 | 11,9 | 50 | | 50 | 1 |
| LH70 | *L.hyperborea* | 27/07/2021 | Bona Sea Rogaland | 59.156389 | 5.495556 | B | 5-10 | 15,2 | 32 | 23 | 10,2 | 50 | | 50 | 1 |
| LH71 | *L.hyperborea* | 27/07/2021 | Bona Sea Rogaland | 59.156389 | 5.495556 | B | 5-10 | 15,2 | 32 | 20 | 2,94 | 50 | | 50 | 1 |
| LH72 | *L.hyperborea* | 27/07/2021 | Bona Sea Rogaland | 59.156389 | 5.495556 | B | 5-10 | 15,2 | 32 | 28 | 14,5 | 50 | | 50 | 1 |
| LH73 | *L.hyperborea* | 27/07/2021 | Bona Sea Rogaland | 59.156389 | 5.495556 | B | 5-10 | 15,2 | 32 | 18 | 7,76 | 50 | | 50 | 1 |
| LH74 | *L.hyperborea* | 27/07/2021 | Bona Sea Rogaland | 59.156389 | 5.495556 | B | 5-10 | 15,2 | 32 | 22 | 10,1 | 50 | | 50 | 1 |
| LH75 | *L.hyperborea* | 27/07/2021 | Bona Sea Rogaland | 59.156389 | 5.495556 | B | 5-10 | 15,2 | 32 | 29,2 | 10,9 | 50 | | 50 | 1 |
| LH76 | *L.hyperborea* | 27/07/2021 | Bona Sea Rogaland | 59.156389 | 5.495556 | B | 5-10 | 15,2 | 32 | 28 | 5,44 | 50 | | 50 | 1 |
| LH77 | *L.hyperborea* | 27/07/2021 | Møre & Romsdal | 62.481667 | 5.670833 | M | 5-10 | 14,7 | 33 | 22 | 3,36 | 50 | | 50 | 1 |
| LH78 | *L.hyperborea* | 27/07/2021 | Møre & Romsdal | 62.481667 | 5.670833 | M | 5-10 | 14,7 | 33 | 19,3 | 11,3 | 50 | | 50 | 1 |
| LH79 | *L.hyperborea* | 27/07/2021 | Møre & Romsdal | 62.481667 | 5.670833 | M | 5-10 | 14,7 | 33 | 25 | 2,62 | 50 | | 50 | 1 |
| LH80 | *L.hyperborea* | 27/07/2021 | Møre & Romsdal | 62.481667 | 5.670833 | M | 5-10 | 14,7 | 33 | 22 | 3,74 | 50 | | 50 | 1 |
| LH81 | *L.hyperborea* | 27/07/2021 | Møre & Romsdal | 62.481667 | 5.670833 | M | 5-10 | 14,7 | 33 | 23 | 1,99 | 50 | | 50 | 1 |
| LH82 | *L.hyperborea* | 27/07/2021 | Møre & Romsdal | 62.481667 | 5.670833 | M | 5-10 | 14,7 | 33 | 25 | 2,14 | 50 | | 50 | 1 |

| | | | | | | | | | | | | | | | |
|---|---|---|---|---|---|---|---|---|---|---|---|---|---|---|---|
| LH83 | *L.hyperborea* | 27/07/2021 | Møre & Romsdal | 62.481667 | 5.670833 | M | 5-10 | 14,7 | 33 | 18 | 8,02 | 50 | | 50 | 1 |
| LH84 | *L.hyperborea* | 27/07/2021 | Møre & Romsdal | 62.481667 | 5.670833 | M | 5-10 | 14,7 | 33 | 25 | 5,98 | 50 | | 50 | 1 |
| LH85 | *L.hyperborea* | 27/07/2021 | Møre & Romsdal | 62.481667 | 5.670833 | M | 5-10 | 14,7 | 33 | 21 | 5,52 | 50 | | 50 | 1 |
| LH86 | *L.hyperborea* | 27/07/2021 | Møre & Romsdal | 62.481667 | 5.670833 | M | 5-10 | 14,7 | 33 | 19 | 3,58 | 50 | | 50 | 1 |
| LH87 | *L.hyperborea* | 27/07/2021 | Møre & Romsdal | 62.481667 | 5.670833 | M | 5-10 | 14,7 | 33 | 25 | 7,32 | 50 | | 50 | 1 |
| LH88 | *L.hyperborea* | 27/07/2021 | Møre & Romsdal | 62.481667 | 5.670833 | M | 5-10 | 14,7 | 33 | 19 | 9,02 | 50 | | 50 | 1 |
| LH89 | *L.hyperborea* | 27/07/2021 | Møre & Romsdal | 62.481667 | 5.670833 | M | 5-10 | 14,7 | 33 | 16 | 7,82 | 50 | | 50 | 1 |
| LH90 | *L.hyperborea* | 27/07/2021 | Møre & Romsdal | 62.481667 | 5.670833 | M | 5-10 | 14,7 | 33 | 19,4 | 9,04 | 50 | | 50 | 1 |
| LH91 | *L.hyperborea* | 27/07/2021 | Møre & Romsdal | 62.481667 | 5.670833 | M | 5-10 | 14,7 | 33 | 19,4 | 6,16 | 50 | | 50 | 1 |
| LH92 | *L.hyperborea* | 27/07/2021 | Møre & Romsdal | 62.481667 | 5.670833 | M | 5-10 | 14,7 | 33 | 14,3 | 4,44 | 50 | | 50 | 1 |
| LH93 | *L.hyperborea* | 27/07/2021 | Møre & Romsdal | 62.481667 | 5.670833 | M | 5-10 | 14,7 | 33 | 17,5 | 8,42 | 50 | | 50 | 1 |
| LH94 | *L.hyperborea* | 27/07/2021 | Møre & Romsdal | 62.481667 | 5.670833 | M | 5-10 | 14,7 | 33 | 16,6 | 5,38 | 50 | | 50 | 1 |
| LH95 | *L.hyperborea* | 27/07/2021 | Møre & Romsdal | 62.481667 | 5.670833 | M | 5-10 | 14,7 | 33 | 21 | 11 | 50 | | 50 | 1 |
| LH96 | *L.hyperborea* | 27/07/2021 | Møre & Romsdal | 62.481667 | 5.670833 | M | 5-10 | 14,7 | 33 | 19 | 5,54 | 50 | | 50 | 1 |
| LH97 | *L.hyperborea* | 27/07/2021 | Møre & Romsdal | 62.481667 | 5.670833 | M | 5-10 | 14,7 | 33 | 15,4 | 5,58 | 50 | | 50 | 1 |
| LH98 | *L.hyperborea* | 27/07/2021 | Møre & Romsdal | 62.481667 | 5.670833 | M | 5-10 | 14,7 | 33 | 24,4 | 2,74 | 50 | | 50 | 1 |
| LH99 | *L.hyperborea* | 27/07/2021 | Møre & Romsdal | 62.481667 | 5.670833 | M | 5-10 | 14,7 | 33 | 24 | 1,81 | 50 | | 50 | 1 |
| LH100 | *L.hyperborea* | 27/07/2021 | Møre & Romsdal | 62.481667 | 5.670833 | M | 5-10 | 14,7 | 33 | 17 | 13 | 50 | | 50 | 1 |
| LH101 | *L.hyperborea* | 27/07/2021 | Møre & Romsdal | 62.481667 | 5.670833 | M | 5-10 | 14,7 | 33 | 14 | 14,5 | 50 | | 50 | 1 |
| LH102 | *L.hyperborea* | 27/07/2021 | Møre & Romsdal | 62.481667 | 5.670833 | M | 5-10 | 14,7 | 33 | 22 | 6,8 | 50 | | 50 | 1 |
| LH103 | *L.hyperborea* | 27/07/2021 | Møre & Romsdal | 62.481667 | 5.670833 | M | 5-10 | 14,7 | 33 | 20 | 17 | 50 | | 50 | 1 |

| | | | | | | | | | | | | | | | |
|---|---|---|---|---|---|---|---|---|---|---|---|---|---|---|---|
| LH104 | *L.hyperborea* | 27/07/2021 | Møre & Romsdal | 62.481667 | 5.670833 | M | 5-10 | 14,7 | 33 | 20 | 15,5 | 50 | | 50 | 1 |
| LH105 | *L.hyperborea* | 27/07/2021 | Møre & Romsdal | 62.481667 | 5.670833 | M | 5-10 | 14,7 | 33 | 22 | 5,94 | 50 | | 50 | 1 |
| LH106 | *L.hyperborea* | 27/07/2021 | Møre & Romsdal | 62.481667 | 5.670833 | M | 5-10 | 14,7 | 33 | 25 | 10 | 50 | | 50 | 1 |
| LH107 | *L.hyperborea* | 27/07/2021 | Møre & Romsdal | 62.481667 | 5.670833 | M | 5-10 | 14,7 | 33 | 18 | 25 | 50 | | 50 | 1 |
| LH108 | *L.hyperborea* | 27/07/2021 | Møre & Romsdal | 62.481667 | 5.670833 | M | 5-10 | 14,7 | 33 | 20 | 12,7 | 50 | | 50 | 1 |
| LH109 | *L.hyperborea* | 27/07/2021 | Møre & Romsdal | 62.481667 | 5.670833 | M | 5-10 | 14,7 | 33 | 20 | 9,88 | 50 | | 50 | 1 |
| LH110 | *L.hyperborea* | 27/07/2021 | Møre & Romsdal | 62.481667 | 5.670833 | M | 5-10 | 14,7 | 33 | 15,2 | 9,58 | 50 | | 50 | 1 |
| LH111 | *L.hyperborea* | 27/07/2021 | Møre & Romsdal | 62.481667 | 5.670833 | M | 5-10 | 14,7 | 33 | 20 | 10,1 | 50 | | 50 | 1 |
| LH112 | *L.hyperborea* | 27/07/2021 | Møre & Romsdal | 62.481667 | 5.670833 | M | 5-10 | 14,7 | 33 | 20 | 15,2 | 50 | | 50 | 1 |
| LH113 | *L.hyperborea* | 27/07/2021 | Møre & Romsdal | 62.481667 | 5.670833 | M | 5-10 | 14,7 | 33 | 17,6 | 11 | 50 | | 50 | 1 |
| LH114 | *L.hyperborea* | 27/07/2021 | Møre & Romsdal | 62.481667 | 5.670833 | M | 5-10 | 14,7 | 33 | 14 | 8,36 | 50 | | 50 | 1 |
| LH115 | *L.hyperborea* | 27/07/2021 | Møre & Romsdal | 62.481667 | 5.670833 | M | 5-10 | 14,7 | 33 | 23 | 7,86 | 50 | | 50 | 1 |
| LH116 | *L.hyperborea* | 27/07/2021 | Møre & Romsdal | 62.481667 | 5.670833 | M | 5-10 | 14,7 | 33 | 21,7 | 12,2 | 50 | | 50 | 1 |
| LH117 | *L.hyperborea* | 08/03/2022 | Korsfjorden, Bergen, Hordaland | 60.157397 | 5.006340 | K | 0-5 | 6,3 | 33 | 23 | 7,28 | 50 | 4,36 | 50 | 1 |
| LH118 | *L.hyperborea* | 08/03/2022 | Korsfjorden, Bergen, Hordaland | 60.157397 | 5.006340 | K | 0-5 | 6,3 | 33 | 27,3 | 20,4 | 50 | 8,72 | 50 | 1 |
| LH119 | *L.hyperborea* | 08/03/2022 | Korsfjorden, Bergen, Hordaland | 60.157397 | 5.006340 | K | 0-5 | 6,3 | 33 | 27 | 52,2 | 50 | 9,06 | 50 | 1 |
| LH120 | *L.hyperborea* | 08/03/2022 | Korsfjorden, Bergen, Hordaland | 60.157397 | 5.006340 | K | 0-5 | 6,3 | 33 | 23,4 | 12,4 | 50 | 2,64 | 50 | 1 |
| LH121 | *L.hyperborea* | 08/03/2022 | Korsfjorden, Bergen, Hordaland | 60.157397 | 5.006340 | K | 0-5 | 6,3 | 33 | 29,9 | 31,6 | 50 | 3,68 | 50 | 1 |
| LH122 | *L.hyperborea* | 08/03/2022 | Korsfjorden, Bergen, Hordaland | 60.157397 | 5.006340 | K | 0-5 | 6,3 | 33 | 33,7 | 38,4 | 50 | 13 | 50 | 1 |
| LH123 | *L.hyperborea* | 08/03/2022 | Korsfjorden, Bergen, Hordaland | 60.157397 | 5.006340 | K | 0-5 | 6,3 | 33 | 21,9 | 32,2 | 50 | 5,74 | 50 | 1 |
| LH124 | *L.hyperborea* | 08/03/2022 | Korsfjorden, Bergen, Hordaland | 60.157397 | 5.006340 | K | 0-5 | 6,3 | 33 | 24,2 | 26,6 | 50 | 7,42 | 50 | 1 |

| | | | | | | | | | | | | | | | |
|---|---|---|---|---|---|---|---|---|---|---|---|---|---|---|---|
| LH125 | *L.hyperborea* | 08/03/2022 | Korsfjorden, Bergen, Hordaland | 60.157397 | 5.006340 | K | 0-5 | 6,3 | 33 | 18,5 | 17,6 | 50 | 9,34 | 50 | 1 |
| LH126 | *L.hyperborea* | 08/03/2022 | Korsfjorden, Bergen, Hordaland | 60.157397 | 5.006340 | K | 0-5 | 6,3 | 33 | 21 | 22,6 | 50 | 2,94 | 50 | 1 |
| LH127 | *L.hyperborea* | 08/03/2022 | Korsfjorden, Bergen, Hordaland | 60.157397 | 5.006340 | K | 0-5 | 6,3 | 33 | 31 | 36,2 | 50 | 14,3 | 50 | 1 |
| LH128 | *L.hyperborea* | 08/03/2022 | Korsfjorden, Bergen, Hordaland | 60.157397 | 5.006340 | K | 0-5 | 6,3 | 33 | 35,2 | 15,4 | 50 | 10,5 | 50 | 1 |
| LH129 | *L.hyperborea* | 08/03/2022 | Korsfjorden, Bergen, Hordaland | 60.157397 | 5.006340 | K | 0-5 | 6,3 | 33 | 29,3 | 15,7 | 50 | 5,24 | 50 | 0 |
| LH130 | *L.hyperborea* | 08/03/2022 | Korsfjorden, Bergen, Hordaland | 60.157397 | 5.006340 | K | 0-5 | 6,3 | 33 | 27 | 36,6 | 50 | 7,94 | 50 | 1 |
| LH131 | *L.hyperborea* | 08/03/2022 | Korsfjorden, Bergen, Hordaland | 60.157397 | 5.006340 | K | 0-5 | 6,3 | 33 | 28,3 | 45,4 | 50 | 7,22 | 50 | 0 |
| LH132 | *L.hyperborea* | 08/03/2022 | Korsfjorden, Bergen, Hordaland | 60.157397 | 5.006340 | K | 0-5 | 6,3 | 33 | 24,5 | 34,4 | 50 | 10,3 | 50 | 1 |
| LH133 | *L.hyperborea* | 08/03/2022 | Korsfjorden, Bergen, Hordaland | 60.157397 | 5.006340 | K | 0-5 | 6,3 | 33 | 20,3 | 9,28 | 50 | 2,98 | 50 | 1 |
| LH134 | *L.hyperborea* | 08/03/2022 | Korsfjorden, Bergen, Hordaland | 60.157397 | 5.006340 | K | 0-5 | 6,3 | 33 | 18,5 | 17,2 | 50 | 4,9 | 50 | 1 |
| LH135 | *L.hyperborea* | 08/03/2022 | Korsfjorden, Bergen, Hordaland | 60.157397 | 5.006340 | K | 0-5 | 6,3 | 33 | 46 | 15 | 50 | 13,2 | 50 | 1 |
| LH136 | *L.hyperborea* | 08/03/2022 | Korsfjorden, Bergen, Hordaland | 60.157397 | 5.006340 | K | 0-5 | 6,3 | 33 | 33,7 | 39,8 | 50 | 6,38 | 50 | 1 |
| LH137 | *L.hyperborea* | 08/03/2022 | Korsfjorden, Bergen, Hordaland | 60.157397 | 5.006340 | K | 0-5 | 6,3 | 33 | 22 | 16,1 | 50 | 2,42 | 50 | 1 |
| LH138 | *L.hyperborea* | 08/03/2022 | Korsfjorden, Bergen, Hordaland | 60.157397 | 5.006340 | K | 0-5 | 6,3 | 33 | 38,2 | 39,4 | 50 | 9,52 | 50 | 1 |
| LH139 | *L.hyperborea* | 08/03/2022 | Korsfjorden, Bergen, Hordaland | 60.157397 | 5.006340 | K | 0-5 | 6,3 | 33 | 30 | 66 | 50 | 10 | 50 | 1 |
| LH140 | *L.hyperborea* | 08/03/2022 | Korsfjorden, Bergen, Hordaland | 60.157397 | 5.006340 | K | 0-5 | 6,3 | 33 | 26 | 30,6 | 50 | 32,2 | 50 | 1 |
| LH141 | *L.hyperborea* | 08/03/2022 | Korsfjorden, Bergen, Hordaland | 60.157397 | 5.006340 | K | 0-5 | 6,3 | 33 | 23,4 | 12,9 | 50 | 3,12 | 50 | 1 |
| LH142 | *L.hyperborea* | 08/03/2022 | Korsfjorden, Bergen, Hordaland | 60.157397 | 5.006340 | K | 0-5 | 6,3 | 33 | 40 | 60,2 | 50 | 8,56 | 50 | 1 |
| LH143 | *L.hyperborea* | 08/03/2022 | Korsfjorden, Bergen, Hordaland | 60.157397 | 5.006340 | K | 0-5 | 6,3 | 33 | 37 | 48,4 | 50 | 8,32 | 50 | 1 |
| LH144 | *L.hyperborea* | 08/03/2022 | Korsfjorden, Bergen, Hordaland | 60.157397 | 5.006340 | K | 0-5 | 6,3 | 33 | 29,8 | 26,6 | 50 | 4,54 | 50 | 1 |
| LH145 | *L.hyperborea* | 08/03/2022 | Korsfjorden, Bergen, Hordaland | 60.157397 | 5.006340 | K | 0-5 | 6,3 | 33 | 30,4 | 14,2 | 50 | 1,49 | 50 | 1 |

| | | | | | | | | | | | | | | | |
|---|---|---|---|---|---|---|---|---|---|---|---|---|---|---|---|
| LH146 | *L.hyperborea* | 08/03/2022 | Korsfjorden, Bergen, Hordaland | 60.157397 | 5.006340 | K | 0-5 | 6,3 | 33 | 38 | 37,6 | 50 | 20,6 | 50 | 1 |
| LH147 | *L.hyperborea* | 08/03/2022 | Korsfjorden, Bergen, Hordaland | 60.157397 | 5.006340 | K | 0-5 | 6,3 | 33 | 32 | 36,4 | 50 | 15,8 | 50 | 1 |
| LH148 | *L.hyperborea* | 08/03/2022 | Korsfjorden, Bergen, Hordaland | 60.157397 | 5.006340 | K | 0-5 | 6,3 | 33 | 33,6 | 49 | 50 | 29 | 50 | 1 |
| LH149 | *L.hyperborea* | 08/03/2022 | Korsfjorden, Bergen, Hordaland | 60.157397 | 5.006340 | K | 0-5 | 6,3 | 33 | 43,8 | 32,6 | 50 | 13,9 | 50 | 1 |
| LH150 | *L.hyperborea* | 08/03/2022 | Korsfjorden, Bergen, Hordaland | 60.157397 | 5.006340 | K | 0-5 | 6,3 | 33 | 38,5 | 28,8 | 50 | 5,5 | 50 | 1 |
| LH151 | *L.hyperborea* | 08/03/2022 | Korsfjorden, Bergen, Hordaland | 60.157397 | 5.006340 | K | 0-5 | 6,3 | 33 | 22 | 14,7 | 50 | 6,62 | 50 | 1 |
| LH152 | *L.hyperborea* | 08/03/2022 | Korsfjorden, Bergen, Hordaland | 60.157397 | 5.006340 | K | 0-5 | 6,3 | 33 | 46,8 | 26,4 | 50 | 8,1 | 50 | 1 |
| LH153 | *L.hyperborea* | 08/03/2022 | Korsfjorden, Bergen, Hordaland | 60.157397 | 5.006340 | K | 0-5 | 6,3 | 33 | 35,7 | 12,9 | 50 | 6,96 | 50 | 1 |
| LH154 | *L.hyperborea* | 08/03/2022 | Korsfjorden, Bergen, Hordaland | 60.157397 | 5.006340 | K | 0-5 | 6,3 | 33 | 23,6 | 24,4 | 50 | 3,56 | 50 | 1 |
| LH155 | *L.hyperborea* | 08/03/2022 | Korsfjorden, Bergen, Hordaland | 60.157397 | 5.006340 | K | 0-5 | 6,3 | 33 | 23,7 | 36,2 | 50 | 12,3 | 50 | 1 |
| LH156 | *L.hyperborea* | 08/03/2022 | Korsfjorden, Bergen, Hordaland | 60.157397 | 5.006340 | K | 0-5 | 6,3 | 33 | 15 | 8,24 | 50 | 1,23 | 50 | 1 |
| LH157 | *L.hyperborea* | 08/03/2022 | Korsfjorden, Bergen, Hordaland | 60.157397 | 5.006340 | K | 0-5 | 6,3 | 33 | 36,2 | 48,2 | 50 | 10,1 | 50 | 1 |
| LH158 | *L.hyperborea* | 08/03/2022 | Korsfjorden, Bergen, Hordaland | 60.157397 | 5.006340 | K | 0-5 | 6,3 | 33 | 33,7 | 18,8 | 50 | 9,42 | 50 | 1 |
| LH159 | *L.hyperborea* | 08/03/2022 | Korsfjorden, Bergen, Hordaland | 60.157397 | 5.006340 | K | 0-5 | 6,3 | 33 | 24,5 | 16,9 | 50 | 2,56 | 50 | 1 |
| LH160 | *L.hyperborea* | 08/03/2022 | Korsfjorden, Bergen, Hordaland | 60.157397 | 5.006340 | K | 0-5 | 6,3 | 33 | 24 | 19,8 | 50 | 5,68 | 50 | 1 |
| LH161 | *L.hyperborea* | 08/03/2022 | Korsfjorden, Bergen, Hordaland | 60.157397 | 5.006340 | K | 0-5 | 6,3 | 33 | 27 | 23 | 50 | 9,64 | 50 | 1 |
| LH162 | *L.hyperborea* | 08/03/2022 | Korsfjorden, Bergen, Hordaland | 60.157397 | 5.006340 | K | 0-5 | 6,3 | 33 | 20,3 | 12,3 | 50 | 2,74 | 50 | 1 |
| LH163 | *L.hyperborea* | 08/03/2022 | Korsfjorden, Bergen, Hordaland | 60.157397 | 5.006340 | K | 0-5 | 6,3 | 33 | 40,8 | 45,8 | 50 | 2,74 | 50 | 1 |
| LH164 | *L.hyperborea* | 08/03/2022 | Korsfjorden, Bergen, Hordaland | 60.157397 | 5.006340 | K | 0-5 | 6,3 | 33 | 34,4 | 68,6 | 50 | 8,1 | 50 | 1 |
| LH165 | *L.hyperborea* | 08/03/2022 | Korsfjorden, Bergen, Hordaland | 60.157397 | 5.006340 | K | 0-5 | 6,3 | 33 | 37,5 | 33,4 | 50 | 2,62 | 50 | 1 |
| LH166 | *L.hyperborea* | 08/03/2022 | Korsfjorden, Bergen, Hordaland | 60.157397 | 5.006340 | K | 0-5 | 6,3 | 33 | 13,8 | 6,84 | 50 | 1,34 | 50 | 1 |

| | | | | | | | | | | | | | | | |
|---|---|---|---|---|---|---|---|---|---|---|---|---|---|---|---|
| LH167 | *L.hyperborea* | 08/03/2022 | Korsfjorden, Bergen, Hordaland | 60.157397 | 5.006340 | K | 0-5 | 6,3 | 33 | 46,3 | 23,4 | 50 | 1,71 | 50 | 1 |
| LH168 | *L.hyperborea* | 08/03/2022 | Korsfjorden, Bergen, Hordaland | 60.157397 | 5.006340 | K | 0-5 | 6,3 | 33 | 23 | 18,3 | 50 | 1,77 | 50 | 1 |
| LH169 | *L.hyperborea* | 08/03/2022 | Korsfjorden, Bergen, Hordaland | 60.157397 | 5.006340 | K | 0-5 | 6,3 | 33 | 22 | 12,1 | 50 | 1,88 | 50 | 1 |
| LH170 | *L.hyperborea* | 08/03/2022 | Korsfjorden, Bergen, Hordaland | 60.157397 | 5.006340 | K | 0-5 | 6,3 | 33 | 30 | 57,4 | 50 | 15,3 | 50 | 1 |
| LH171 | *L.hyperborea* | 08/03/2022 | Korsfjorden, Bergen, Hordaland | 60.157397 | 5.006340 | K | 0-5 | 6,3 | 33 | 21,5 | 11,5 | 50 | 2,6 | 50 | 1 |
| LH172 | *L.hyperborea* | 08/03/2022 | Korsfjorden, Bergen, Hordaland | 60.157397 | 5.006340 | K | 0-5 | 6,3 | 33 | 23,9 | 13,3 | 50 | 19,9 | 50 | 1 |
| LH173 | *L.hyperborea* | 08/03/2022 | Korsfjorden, Bergen, Hordaland | 60.157397 | 5.006340 | K | 0-5 | 6,3 | 33 | 31,8 | 73,6 | 50 | 17,2 | 50 | 1 |
| LH174 | *L.hyperborea* | 08/03/2022 | Korsfjorden, Bergen, Hordaland | 60.157397 | 5.006340 | K | 0-5 | 6,3 | 33 | 27,5 | 30,6 | 50 | 19,8 | 50 | 1 |
| LH175 | *L.hyperborea* | 23/01/2022 | Korsfjorden, Bergen, Hordaland | 60.157397 | 5.006340 | K | 0-5 | 7,2 | 33 | 27 | 34,2 | 50 | 4,76 | 50 | 1 |
| LH176 | *L.hyperborea* | 23/01/2022 | Korsfjorden, Bergen, Hordaland | 60.157397 | 5.006340 | K | 0-5 | 7,2 | 33 | 31 | 24 | 50 | 8,32 | 50 | 1 |
| LH177 | *L.hyperborea* | 23/01/2022 | Korsfjorden, Bergen, Hordaland | 60.157397 | 5.006340 | K | 0-5 | 7,2 | 33 | 31 | too high | 50 | 52,4 | 50 | 1 |
| LH178 | *L.hyperborea* | 23/01/2022 | Korsfjorden, Bergen, Hordaland | 60.157397 | 5.006340 | K | 0-5 | 7,2 | 33 | 20,9 | 36,8 | 50 | 30,2 | 50 | 1 |
| LH179 | *L.hyperborea* | 23/01/2022 | Korsfjorden, Bergen, Hordaland | 60.157397 | 5.006340 | K | 0-5 | 7,2 | 33 | 28 | 49,4 | 50 | 28,4 | 50 | 1 |
| LH180 | *L.hyperborea* | 08/03/2022 | Korsfjorden, Bergen, Hordaland | 60.157397 | 5.006340 | K | 0-5 | 6,3 | 33 | 22,5 | 23,8 | 50 | 7,24 | 50 | 1 |
| LH181 | *L.hyperborea* | 08/03/2022 | Korsfjorden, Bergen, Hordaland | 60.157397 | 5.006340 | K | 0-5 | 6,3 | 33 | 25,7 | 54 | 50 | 11,6 | 50 | 1 |
| LH182 | *L.hyperborea* | 08/03/2022 | Korsfjorden, Bergen, Hordaland | 60.157397 | 5.006340 | K | 0-5 | 6,3 | 33 | 22,2 | 73,2 | 50 | 6,6 | 50 | 1 |
| LH183 | *L.hyperborea* | 08/03/2022 | Korsfjorden, Bergen, Hordaland | 60.157397 | 5.006340 | K | 0-5 | 6,3 | 33 | 28,7 | 27,2 | 50 | 13,1 | 50 | 1 |
| LH184 | *L.hyperborea* | 08/03/2022 | Korsfjorden, Bergen, Hordaland | 60.157397 | 5.006340 | K | 0-5 | 6,3 | 33 | 38,4 | 21,8 | 50 | 21,6 | 50 | 1 |
| LH185 | *L.hyperborea* | 08/03/2022 | Korsfjorden, Bergen, Hordaland | 60.157397 | 5.006340 | K | 0-5 | 6,3 | 33 | 34,8 | 74,8 | 50 | 14,6 | 50 | 1 |
| LH186 | *L.hyperborea* | 08/03/2022 | Korsfjorden, Bergen, Hordaland | 60.157397 | 5.006340 | K | 0-5 | 6,3 | 33 | 26,7 | 17,4 | 50 | 7,04 | 50 | 1 |
| LH187 | *L.hyperborea* | 08/03/2022 | Korsfjorden, Bergen, Hordaland | 60.157397 | 5.006340 | K | 0-5 | 6,3 | 33 | 16,8 | 17,5 | 50 | 8,62 | 50 | 1 |

| | | | | | | | | | | | | | | | |
|---|---|---|---|---|---|---|---|---|---|---|---|---|---|---|---|
| LH188 | *L.hyperborea* | 08/03/2022 | Korsfjorden, Bergen, Hordaland | 60.157397 | 5.006340 | K | 0-5 | 6,3 | 33 | 62,3 | 27,8 | 50 | 6,1 | 50 | 1 |
| LH189 | *L.hyperborea* | 08/03/2022 | Korsfjorden, Bergen, Hordaland | 60.157397 | 5.006340 | K | 0-5 | 6,3 | 33 | 28,2 | 49 | 50 | 28,4 | 50 | 1 |
| LH190 | *L.hyperborea* | 08/03/2022 | Korsfjorden, Bergen, Hordaland | 60.157397 | 5.006340 | K | 0-5 | 6,3 | 33 | 26,4 | 30,4 | 50 | 8,6 | 50 | 1 |
| LH191 | *L.hyperborea* | 08/03/2022 | Korsfjorden, Bergen, Hordaland | 60.157397 | 5.006340 | K | 0-5 | 6,3 | 33 | 18,7 | 15,8 | 50 | 11,1 | 50 | 1 |
| LH192 | *L.hyperborea* | 08/03/2022 | Korsfjorden, Bergen, Hordaland | 60.157397 | 5.006340 | K | 0-5 | 6,3 | 33 | 22,5 | 33,8 | 50 | 16,4 | 50 | 1 |
| LH193 | *L.hyperborea* | 08/03/2022 | Korsfjorden, Bergen, Hordaland | 60.157397 | 5.006340 | K | 0-5 | 6,3 | 33 | 25,4 | 49,6 | 50 | 24 | 50 | 1 |
| LH194 | *L.hyperborea* | 08/03/2022 | Korsfjorden, Bergen, Hordaland | 60.157397 | 5.006340 | K | 0-5 | 6,3 | 33 | 23,4 | 21,4 | 50 | 7,84 | 50 | 1 |
| LH195 | *L.hyperborea* | 08/03/2022 | Korsfjorden, Bergen, Hordaland | 60.157397 | 5.006340 | K | 0-5 | 6,3 | 33 | 23,6 | 93,6 | 50 | 54,2 | 50 | 1 |
| LH196 | *L.hyperborea* | 08/03/2022 | Korsfjorden, Bergen, Hordaland | 60.157397 | 5.006340 | K | 0-5 | 6,3 | 33 | 30,3 | 29 | 50 | 2,4 | 50 | 1 |
| LH197 | *L.hyperborea* | 08/03/2022 | Korsfjorden, Bergen, Hordaland | 60.157397 | 5.006340 | K | 0-5 | 6,3 | 33 | 26,6 | 29,6 | 50 | 6,94 | 50 | 1 |
| LH198 | *L.hyperborea* | 08/03/2022 | Korsfjorden, Bergen, Hordaland | 60.157397 | 5.006340 | K | 0-5 | 6,3 | 33 | 38,8 | 33,8 | 50 | 16,2 | 50 | 1 |
| LH199 | *L.hyperborea* | 08/03/2022 | Korsfjorden, Bergen, Hordaland | 60.157397 | 5.006340 | K | 0-5 | 6,3 | 33 | 32,9 | 64,2 | 50 | 16,8 | 50 | 1 |
| LH200 | *L.hyperborea* | 08/03/2022 | Korsfjorden, Bergen, Hordaland | 60.157397 | 5.006340 | K | 0-5 | 6,3 | 33 | 40,5 | 22,2 | 50 | 20 | 50 | 1 |
| LH201 | *L.hyperborea* | 23/01/2022 | Korsfjorden, Bergen, Hordaland | 60.157397 | 5.006340 | K | 0-5 | 7,2 | 33 | 26,7 | 23,8 | 50 | 14,8 | 50 | 1 |
| LH202 | *L.hyperborea* | 23/01/2022 | Korsfjorden, Bergen, Hordaland | 60.157397 | 5.006340 | K | 0-5 | 7,2 | 33 | 25,6 | 27 | 50 | 4,38 | 50 | 1 |
| LH203 | *L.hyperborea* | 23/01/2022 | Korsfjorden, Bergen, Hordaland | 60.157397 | 5.006340 | K | 0-5 | 7,2 | 33 | 25 | 13,5 | 50 | 2,46 | 50 | 1 |
| LH204 | *L.hyperborea* | 23/01/2022 | Korsfjorden, Bergen, Hordaland | 60.157397 | 5.006340 | K | 0-5 | 7,2 | 33 | 24,3 | 58,6 | 50 | 26,6 | 50 | 1 |
| LH205 | *L.hyperborea* | 23/01/2022 | Korsfjorden, Bergen, Hordaland | 60.157397 | 5.006340 | K | 0-5 | 7,2 | 33 | 24 | 17 | 50 | 3,9 | 50 | 1 |
| LH206 | *L.hyperborea* | 23/01/2022 | Korsfjorden, Bergen, Hordaland | 60.157397 | 5.006340 | K | 0-5 | 7,2 | 33 | 21,7 | 10,6 | 50 | 3,62 | 50 | 1 |
| LH207 | *L.hyperborea* | 23/01/2022 | Korsfjorden, Bergen, Hordaland | 60.157397 | 5.006340 | K | 0-5 | 7,2 | 33 | 20,7 | 24,8 | 50 | 9,26 | 50 | 1 |
| LH208 | *L.hyperborea* | 15/06/2022 | Korsfjorden, Bergen, Hordaland | 60.157397 | 5.006340 | K | 0-5 | 11,9 | 33 | 21,3 | 11,7 | 50 | 3,34 | 50 | 1 |

| | | | | | | | | | | | | | | | |
|---|---|---|---|---|---|---|---|---|---|---|---|---|---|---|---|
| LH209 | *L.hyperborea* | 15/06/2022 | Korsfjorden, Bergen, Hordaland | 60.157397 | 5.006340 | K | 0-5 | 11,9 | 33 | 18,7 | 8,18 | 50 | 6,38 | 50 | 1 |
| LH210 | *L.hyperborea* | 15/06/2022 | Korsfjorden, Bergen, Hordaland | 60.157397 | 5.006340 | K | 0-5 | 11,9 | 33 | 23,1 | 9 | 50 | 1,56 | 50 | 1 |
| LH211 | *L.hyperborea* | 15/06/2022 | Korsfjorden, Bergen, Hordaland | 60.157397 | 5.006340 | K | 0-5 | 11,9 | 33 | 25,5 | 10,1 | 50 | 2,34 | 50 | 1 |
| LH212 | *L.hyperborea* | 15/06/2022 | Korsfjorden, Bergen, Hordaland | 60.157397 | 5.006340 | K | 0-5 | 11,9 | 33 | 24,9 | 9,38 | 50 | 1,82 | 50 | 1 |
| LH213 | *L.hyperborea* | 15/06/2022 | Korsfjorden, Bergen, Hordaland | 60.157397 | 5.006340 | K | 0-5 | 11,9 | 33 | 25,7 | 14,3 | 50 | 1,03 | 50 | 0 |
| LH214 | *L.hyperborea* | 15/06/2022 | Korsfjorden, Bergen, Hordaland | 60.157397 | 5.006340 | K | 0-5 | 11,9 | 33 | 28 | 7,08 | 50 | 6,96 | 50 | 1 |
| LH215 | *L.hyperborea* | 15/06/2022 | Korsfjorden, Bergen, Hordaland | 60.157397 | 5.006340 | K | 0-5 | 11,9 | 33 | 23,4 | 6,24 | 50 | 5,48 | 50 | 1 |
| LH216 | *L.hyperborea* | 15/06/2022 | Korsfjorden, Bergen, Hordaland | 60.157397 | 5.006340 | K | 0-5 | 11,9 | 33 | 28,2 | 10,1 | 50 | 1,51 | 50 | 1 |
| LH217 | *L.hyperborea* | 15/06/2022 | Korsfjorden, Bergen, Hordaland | 60.157397 | 5.006340 | K | 0-5 | 11,9 | 33 | 17,2 | 5,4 | 50 | 1,79 | 50 | 1 |
| LH218 | *L.hyperborea* | 15/06/2022 | Korsfjorden, Bergen, Hordaland | 60.157397 | 5.006340 | K | 0-5 | 11,9 | 33 | 18,3 | 6,72 | 50 | 3,48 | 50 | 1 |
| LH219 | *L.hyperborea* | 15/06/2022 | Korsfjorden, Bergen, Hordaland | 60.157397 | 5.006340 | K | 0-5 | 11,9 | 33 | 16,9 | 5,7 | 50 | 3,46 | 50 | 1 |
| LH220 | *L.hyperborea* | 15/06/2022 | Korsfjorden, Bergen, Hordaland | 60.157397 | 5.006340 | K | 0-5 | 11,9 | 33 | 17 | 8,74 | 50 | 2,42 | 50 | 1 |
| LH221 | *L.hyperborea* | 15/06/2022 | Korsfjorden, Bergen, Hordaland | 60.157397 | 5.006340 | K | 0-5 | 11,9 | 33 | 21,5 | 9,3 | 50 | 4,9 | 50 | 1 |
| LH222 | *L.hyperborea* | 15/06/2022 | Korsfjorden, Bergen, Hordaland | 60.157397 | 5.006340 | K | 0-5 | 11,9 | 33 | 18,3 | 11,6 | 50 | 2,26 | 50 | 1 |
| LH223 | *L.hyperborea* | 15/06/2022 | Korsfjorden, Bergen, Hordaland | 60.157397 | 5.006340 | K | 0-5 | 11,9 | 33 | 29,1 | 8,38 | 50 | 1,25 | 50 | 1 |
| LH224 | *L.hyperborea* | 15/06/2022 | Korsfjorden, Bergen, Hordaland | 60.157397 | 5.006340 | K | 0-5 | 11,9 | 33 | 28,1 | 16,3 | 50 | 2,74 | 50 | 1 |
| LH225 | *L.hyperborea* | 15/06/2022 | Korsfjorden, Bergen, Hordaland | 60.157397 | 5.006340 | K | 0-5 | 11,9 | 33 | 27,6 | 18 | 50 | 12 | 50 | 1 |
| LH226 | *L.hyperborea* | 15/06/2022 | Korsfjorden, Bergen, Hordaland | 60.157397 | 5.006340 | K | 0-5 | 11,9 | 33 | 24,5 | 10,6 | 50 | 7,32 | 50 | 1 |
| LH227 | *L.hyperborea* | 15/06/2022 | Korsfjorden, Bergen, Hordaland | 60.157397 | 5.006340 | K | 0-5 | 11,9 | 33 | 17,7 | 5,34 | 50 | 3,16 | 50 | 1 |
| LH228 | *L.hyperborea* | 15/06/2022 | Korsfjorden, Bergen, Hordaland | 60.157397 | 5.006340 | K | 0-5 | 11,9 | 33 | 12,2 | 3,52 | 50 | 3,08 | 50 | 1 |
| LH229 | *L.hyperborea* | 15/06/2022 | Korsfjorden, Bergen, Hordaland | 60.157397 | 5.006340 | K | 0-5 | 11,9 | 33 | 30,1 | 2,68 | 50 | 0,8 | 50 | 1 |

| | | | | | | | | | | | | | | | |
|---|---|---|---|---|---|---|---|---|---|---|---|---|---|---|---|
| LH230 | *L.hyperborea* | 15/06/2022 | Korsfjorden, Bergen, Hordaland | 60.157397 | 5.006340 | K | 0-5 | 11,9 | 33 | 31,9 | 9,54 | 50 | 3,56 | 50 | 1 |
| LH231 | *L.hyperborea* | 15/06/2022 | Korsfjorden, Bergen, Hordaland | 60.157397 | 5.006340 | K | 0-5 | 11,9 | 33 | 30 | 13,3 | 50 | 2,28 | 50 | 1 |
| LH232 | *L.hyperborea* | 15/06/2022 | Korsfjorden, Bergen, Hordaland | 60.157397 | 5.006340 | K | 0-5 | 11,9 | 33 | 29,8 | 10,4 | 50 | 4,18 | 50 | 1 |
| LH233 | *L.hyperborea* | 15/06/2022 | Korsfjorden, Bergen, Hordaland | 60.157397 | 5.006340 | K | 0-5 | 11,9 | 33 | 23,4 | 12 | 50 | 5,8 | 50 | 1 |
| LH234 | *L.hyperborea* | 15/06/2022 | Korsfjorden, Bergen, Hordaland | 60.157397 | 5.006340 | K | 0-5 | 11,9 | 33 | 27,3 | 4,2 | 50 | 2,7 | 50 | 1 |
| LH235 | *L.hyperborea* | 15/06/2022 | Korsfjorden, Bergen, Hordaland | 60.157397 | 5.006340 | K | 0-5 | 11,9 | 33 | 34 | 8,48 | 50 | 7,9 | 50 | 1 |
| LH236 | *L.hyperborea* | 15/06/2022 | Korsfjorden, Bergen, Hordaland | 60.157397 | 5.006340 | K | 0-5 | 11,9 | 33 | 28,3 | 9,04 | 50 | 4,36 | 50 | 1 |
| LH237 | *L.hyperborea* | 15/06/2022 | Korsfjorden, Bergen, Hordaland | 60.157397 | 5.006340 | K | 0-5 | 11,9 | 33 | 30,4 | 13,8 | 50 | 10,7 | 50 | 1 |
| LH238 | *L.hyperborea* | 15/06/2022 | Korsfjorden, Bergen, Hordaland | 60.157397 | 5.006340 | K | 0-5 | 11,9 | 33 | 36,3 | 13,3 | 50 | 8,18 | 50 | 1 |
| LH239 | *L.hyperborea* | 15/06/2022 | Korsfjorden, Bergen, Hordaland | 60.157397 | 5.006340 | K | 0-5 | 11,9 | 33 | 22,6 | 16 | 50 | 10,5 | 50 | 1 |
| LH240 | *L.hyperborea* | 15/06/2022 | Korsfjorden, Bergen, Hordaland | 60.157397 | 5.006340 | K | 0-5 | 11,9 | 33 | 30,8 | 17,1 | 50 | 2,28 | 50 | 1 |
| LH241 | *L.hyperborea* | 15/06/2022 | Korsfjorden, Bergen, Hordaland | 60.157397 | 5.006340 | K | 0-5 | 11,9 | 33 | 27,5 | 6,22 | 50 | 3 | 50 | 1 |
| LH242 | *L.hyperborea* | 15/06/2022 | Korsfjorden, Bergen, Hordaland | 60.157397 | 5.006340 | K | 0-5 | 11,9 | 33 | 26,9 | 8,64 | 50 | 8,76 | 50 | 1 |
| LH243 | *L.hyperborea* | 15/06/2022 | Korsfjorden, Bergen, Hordaland | 60.157397 | 5.006340 | K | 0-5 | 11,9 | 33 | 30,3 | 7,92 | 50 | 3,72 | 50 | 1 |
| LH244 | *L.hyperborea* | 15/06/2022 | Korsfjorden, Bergen, Hordaland | 60.157397 | 5.006340 | K | 0-5 | 11,9 | 33 | 28,1 | 4,94 | 50 | 4,54 | 50 | 1 |
| LH245 | *L.hyperborea* | 15/06/2022 | Korsfjorden, Bergen, Hordaland | 60.157397 | 5.006340 | K | 0-5 | 11,9 | 33 | 30,9 | 3,94 | 50 | 1,91 | 50 | 1 |
| LH246 | *L.hyperborea* | 15/06/2022 | Korsfjorden, Bergen, Hordaland | 60.157397 | 5.006340 | K | 0-5 | 11,9 | 33 | 30,9 | 10,9 | 50 | 1,9 | 50 | 1 |
| LH247 | *L.hyperborea* | 15/06/2022 | Korsfjorden, Bergen, Hordaland | 60.157397 | 5.006340 | K | 0-5 | 11,9 | 33 | 31 | 11,3 | 50 | 5,12 | 50 | 1 |
| LH248 | *L.hyperborea* | 26/10/2022 | Korsfjorden, Bergen, Hordaland | 60.157397 | 5.006340 | K | 0-5 | 11,3 | 32 | 19,9 | 2,08 | 50 | 1,19 | 50 | 1 |
| LH249 | *L.hyperborea* | 26/10/2022 | Korsfjorden, Bergen, Hordaland | 60.157397 | 5.006340 | K | 0-5 | 11,3 | 32 | 36,8 | 6,82 | 50 | 1,41 | 50 | 1 |
| LH250 | *L.hyperborea* | 26/10/2022 | Korsfjorden, Bergen, Hordaland | 60.157397 | 5.006340 | K | 0-5 | 11,3 | 32 | 15,8 | 3,08 | 50 | 1,28 | 50 | 1 |

| | | | | | | | | | | | | | | | |
|---|---|---|---|---|---|---|---|---|---|---|---|---|---|---|---|
| LH251 | *L.hyperborea* | 26/10/2022 | Korsfjorden, Bergen, Hordaland | 60.157397 | 5.006340 | K | 0-5 | 11,3 | 32 | 25 | 6,06 | 50 | 3,94 | 50 | 1 |
| LH252 | *L.hyperborea* | 26/10/2022 | Korsfjorden, Bergen, Hordaland | 60.157397 | 5.006340 | K | 0-5 | 11,3 | 32 | 27,5 | 5,66 | 50 | 5,8 | 50 | 1 |
| LH253 | *L.hyperborea* | 26/10/2022 | Korsfjorden, Bergen, Hordaland | 60.157397 | 5.006340 | K | 0-5 | 11,3 | 32 | 17,6 | 3,3 | 50 | 1,8 | 50 | 1 |
| LH254 | *L.hyperborea* | 26/10/2022 | Korsfjorden, Bergen, Hordaland | 60.157397 | 5.006340 | K | 0-5 | 11,3 | 32 | 21,6 | 2,28 | 50 | 1 | 50 | 1 |
| LH255 | *L.hyperborea* | 26/10/2022 | Korsfjorden, Bergen, Hordaland | 60.157397 | 5.006340 | K | 0-5 | 11,3 | 32 | 18,5 | 3,9 | 50 | 1,7 | 50 | 1 |
| LH256 | *L.hyperborea* | 26/10/2022 | Korsfjorden, Bergen, Hordaland | 60.157397 | 5.006340 | K | 0-5 | 11,3 | 32 | 40,2 | 11,6 | 50 | 4 | 50 | 1 |
| LH257 | *L.hyperborea* | 26/10/2022 | Korsfjorden, Bergen, Hordaland | 60.157397 | 5.006340 | K | 0-5 | 11,3 | 32 | 27,2 | 3,94 | 50 | 1,68 | 50 | 1 |
| LH258 | *L.hyperborea* | 26/10/2022 | Korsfjorden, Bergen, Hordaland | 60.157397 | 5.006340 | K | 0-5 | 11,3 | 32 | 28,6 | 6,72 | 50 | 2,44 | 50 | 1 |
| LH259 | *L.hyperborea* | 26/10/2022 | Korsfjorden, Bergen, Hordaland | 60.157397 | 5.006340 | K | 0-5 | 11,3 | 32 | 26,8 | 5,4 | 50 | 1,15 | 50 | 1 |
| LH260 | *L.hyperborea* | 26/10/2022 | Korsfjorden, Bergen, Hordaland | 60.157397 | 5.006340 | K | 0-5 | 11,3 | 32 | 22,1 | 2,92 | 50 | 0,606 | 50 | 1 |
| LH261 | *L.hyperborea* | 26/10/2022 | Korsfjorden, Bergen, Hordaland | 60.157397 | 5.006340 | K | 0-5 | 11,3 | 32 | 23 | 2,28 | 50 | 0,688 | 50 | 1 |
| LH262 | *L.hyperborea* | 26/10/2022 | Korsfjorden, Bergen, Hordaland | 60.157397 | 5.006340 | K | 0-5 | 11,3 | 32 | 24,1 | 2,34 | 50 | 0,396 | 50 | 1 |
| LH263 | *L.hyperborea* | 26/10/2022 | Korsfjorden, Bergen, Hordaland | 60.157397 | 5.006340 | K | 0-5 | 11,3 | 32 | 40,4 | 3,54 | 50 | 1,27 | 50 | 1 |
| LH264 | *L.hyperborea* | 26/10/2022 | Korsfjorden, Bergen, Hordaland | 60.157397 | 5.006340 | K | 0-5 | 11,3 | 32 | 53,5 | 6,46 | 50 | 3,44 | 50 | 1 |
| LH265 | *L.hyperborea* | 26/10/2022 | Korsfjorden, Bergen, Hordaland | 60.157397 | 5.006340 | K | 0-5 | 11,3 | 32 | 42,8 | 3,8 | 50 | 3,44 | 50 | 1 |
| LH266 | *L.hyperborea* | 26/10/2022 | Korsfjorden, Bergen, Hordaland | 60.157397 | 5.006340 | K | 0-5 | 11,3 | 32 | 30,7 | 17,3 | 50 | 5,54 | 50 | 1 |
| LH267 | *L.hyperborea* | 26/10/2022 | Korsfjorden, Bergen, Hordaland | 60.157397 | 5.006340 | K | 0-5 | 11,3 | 32 | 36,2 | 2,74 | 50 | 4,24 | 50 | 1 |
| LH268 | *L.hyperborea* | 26/10/2022 | Korsfjorden, Bergen, Hordaland | 60.157397 | 5.006340 | K | 0-5 | 11,3 | 32 | 27,8 | 3,06 | 50 | 3,4 | 50 | 1 |
| LH269 | *L.hyperborea* | 26/10/2022 | Korsfjorden, Bergen, Hordaland | 60.157397 | 5.006340 | K | 0-5 | 11,3 | 32 | 41,2 | 2 | 50 | 0,332 | 50 | 1 |
| LH270 | *L.hyperborea* | 26/10/2022 | Korsfjorden, Bergen, Hordaland | 60.157397 | 5.006340 | K | 0-5 | 11,3 | 32 | 36,2 | 2,26 | 50 | 0,794 | 50 | 1 |

**Table S3: Sequencing samples information.**

| Sample ID | Species | Location | Date | Concentration ng/ul (50 ul) | ONT sequencing run ID | ONT sequencing barcode | # reads | # reads mapped vs NC_002687.1 (EsV-1) | # reads mapped vs NC_011183.1 (FsV-158) | # master Variants | A | B | C |
|---|---|---|---|---|---|---|---|---|---|---|---|---|---|
| SL65 | *S. Latissima* | Bergen | 23.01.2022 | 17,9 | 11OCT22-1 | 81 | 2681 | 2 | 2 | 2 | 1 | 0 | 1 |
| SL66 | *S. Latissima* | Bergen | 23.01.2022 | 30 | 11OCT22-1 | 82 | 29963 | 916 | 53 | 3 | 1 | 1 | 1 |
| SL68 | *S. Latissima* | Bergen | 23.01.2022 | 17,4 | 11OCT22-1 | 83 | 21497 | 20 | 22 | 2 | 1 | 0 | 1 |
| SL69 | *S. Latissima* | Bergen | 23.01.2022 | 23,6 | 11OCT22-1 | 84 | 26069 | 0 | 164 | 2 | 1 | 0 | 1 |
| SL74 | *S. Latissima* | Bergen | 23.01.2022 | 20,2 | 11OCT22-1 | 85 | 7891 | 572 | 3 | 3 | 1 | 1 | 1 |
| SL76 | *S. Latissima* | Bergen | 23.01.2022 | 24,6 | 11OCT22-1 | 86 | 15833 | 144 | 6 | 3 | 1 | 1 | 1 |
| SL78 | *S. Latissima* | Bergen | 23.01.2022 | 37,2 | 11OCT22-1 | 87 | 17130 | 89 | 5 | 2 | 1 | 0 | 1 |
| SL81 | *S. Latissima* | Bergen | 23.01.2022 | 26,6 | 11OCT22-1 | 88 | 19349 | 709 | 0 | 3 | 1 | 1 | 1 |
| SL91 | *S. Latissima* | Bergen | 23.01.2022 | 23 | 18JAN23DM1 | 1 | 22859 | 44 | 9 | 3 | 1 | 1 | 1 |
| SL96 | *S. Latissima* | Bergen | 23.01.2022 | 21,8 | 18JAN23DM1 | 2 | 34060 | 77 | 99 | 1 | 1 | 0 | 0 |
| SL97 | *S. Latissima* | Bergen | 03.08.2022 | 51,2 | 18JAN23DM1 | 3 | 6839 | 1079 | 3 | 3 | 1 | 1 | 1 |
| SL100 | *S. Latissima* | Bergen | 03.08.2022 | 35 | 18JAN23DM1 | 4 | 9642 | 2808 | 35 | 3 | 1 | 1 | 1 |
| SL104 | *S. Latissima* | Bergen | 03.08.2022 | 29,4 | 18JAN23DM1 | 5 | 23853 | 378 | 3 | 3 | 1 | 1 | 1 |
| SL108 | *S. Latissima* | Bergen | 03.08.2022 | 23,6 | 18JAN23DM1 | 6 | 4331 | 0 | 177 | 2 | 1 | 0 | 1 |
| SL111 | *S. Latissima* | Bergen | 03.08.2022 | 32 | 18JAN23DM1 | 7 | 13712 | 167 | 3 | 3 | 1 | 1 | 1 |
| SL112 | *S. Latissima* | Bergen | 03.08.2022 | 28 | 18JAN23DM1 | 8 | 13175 | 2 | 0 | 2 | 0 | 0 | 1 |
| SL116 | *S. Latissima* | Bergen | 03.08.2022 | 24 | 18JAN23DM1 | 9 | 14813 | 1328 | 0 | 3 | 1 | 1 | 1 |
| SL118 | *S. Latissima* | Bergen | 03.08.2022 | 30,4 | 18JAN23DM1 | 10 | 273 | 0 | 0 | | | | |
| SL128 | *S. Latissima* | Bergen | 03.08.2022 | 27,4 | 18JAN23DM1 | 11 | 33680 | 196 | 12 | 3 | 1 | 1 | 1 |
| LH139 | *L.hyperborea* | Bergen, Hordaland | 03.08.2022 | 66 | 13JAN23DM1 | 17 | 15486 | 4157 | 1287 | 2 | 1 | 0 | 1 |
| LH119 | *L.hyperborea* | Bergen, Hordaland | 03.08.2022 | 52,2 | 13JAN23DM1 | 18 | 14153 | 501 | 2 | 3 | 1 | 1 | 1 |
| LH178 | *L.hyperborea* | Bergen, Hordaland | 23.01.2022 | 36,8 | 13JAN23DM1 | 25 | 12257 | 3 | 0 | 2 | 1 | 0 | 1 |
| LH177 | *L.hyperborea* | Bergen, Hordaland | 23.01.2022 | too high | 13JAN23DM1 | 26 | 10691 | 2 | 0 | 2 | 1 | 0 | 1 |
| LH173 | *L.hyperborea* | Bergen, Hordaland | 03.08.2022 | 73,6 | 13JAN23DM1 | 27 | 12000 | 253 | 7 | 2 | 1 | 0 | 1 |
| LH170 | *L.hyperborea* | Bergen, Hordaland | 03.08.2022 | 57,4 | 13JAN23DM1 | 28 | 12536 | 2 | 3 | 2 | 1 | 0 | 1 |
| LH157 | *L.hyperborea* | Bergen, Hordaland | 03.08.2022 | 48,2 | 13JAN23DM1 | 29 | 2952 | 3 | 2 | 2 | 1 | 0 | 1 |

| | | | | | | | | | | | | | |
|---|---|---|---|---|---|---|---|---|---|---|---|---|---|
| LH148 | *L.hyperborea* | Bergen, Hordaland | 03.08.2022 | 49 | 13JAN23DM1 | 30 | 10081 | 3 | 2 | 2 | 1 | 0 | 1 |
| LH143 | *L.hyperborea* | Bergen, Hordaland | 03.08.2022 | 48,4 | 13JAN23DM1 | 31 | 5596 | 2 | 0 | 2 | 1 | 0 | 1 |
| LH142 | *L.hyperborea* | Bergen, Hordaland | 03.08.2022 | 60,2 | 13JAN23DM1 | 32 | 10598 | 0 | 7 | 2 | 1 | 0 | 1 |
| LH185 | *L.hyperborea* | Bergen, Hordaland | 03.08.2022 | 74,8 | 13JAN23DM1 | 33 | 1305 | 248 | 35 | 2 | 0 | 1 | 1 |
| LH182 | *L.hyperborea* | Bergen, Hordaland | 03.08.2022 | 73,2 | 13JAN23DM1 | 34 | 4202 | 714 | 291 | 2 | 0 | 1 | 1 |
| LH181 | *L.hyperborea* | Bergen, Hordaland | 03.08.2022 | 54 | 13JAN23DM1 | 35 | 9914 | 851 | 2 | 3 | 1 | 1 | 1 |
| LH179 | *L.hyperborea* | Bergen, Hordaland | 23.01.2022 | 49,4 | 13JAN23DM1 | 36 | 9399 | 4 | 0 | 2 | 1 | 0 | 1 |
| LH193 | *L.hyperborea* | Bergen, Hordaland | 03.08.2022 | 49,6 | 13JAN23DM1 | 38 | 15586 | 326 | 0 | 2 | 1 | 0 | 1 |
| LH189 | *L.hyperborea* | Bergen, Hordaland | 03.08.2022 | 49 | 13JAN23DM1 | 40 | 9720 | 3 | 0 | 2 | 1 | 0 | 1 |
| LH224 | *L.hyperborea* | Bergen, Hordaland | 15.06.2022 | 16,3 | 13JAN23DM1 | 41 | 3918 | 59 | 0 | 2 | 1 | 0 | 1 |
| LH207 | *L.hyperborea* | Bergen, Hordaland | 23.01.2022 | 24,8 | 13JAN23DM1 | 42 | 5841 | 0 | 0 | 2 | 1 | 0 | 1 |
| LH205 | *L.hyperborea* | Bergen, Hordaland | 23.01.2022 | 17 | 13JAN23DM1 | 43 | 13568 | 6 | 0 | 2 | 1 | 0 | 1 |
| LH204 | *L.hyperborea* | Bergen, Hordaland | 23.01.2022 | 58,6 | 13JAN23DM1 | 44 | 10792 | 3 | 0 | 3 | 1 | 1 | 1 |
| LH202 | *L.hyperborea* | Bergen, Hordaland | 23.01.2022 | 27 | 13JAN23DM1 | 45 | 13882 | 3 | 0 | 2 | 1 | 0 | 1 |
| LH201 | *L.hyperborea* | Bergen, Hordaland | 23.01.2022 | 23,8 | 13JAN23DM1 | 46 | 16305 | 5 | 11 | 3 | 1 | 1 | 1 |
| LH199 | *L.hyperborea* | Bergen, Hordaland | 03.08.2022 | 64,2 | 13JAN23DM1 | 47 | 9199 | 3 | 29 | 2 | 1 | 0 | 1 |
| LH195 | *L.hyperborea* | Bergen, Hordaland | 03.08.2022 | 93,6 | 13JAN23DM1 | 48 | 10809 | 317 | 180 | 2 | 1 | 0 | 1 |
| LH255 | *L.hyperborea* | Bergen, Hordaland | 26.10.2022 | 3,9 | 13JAN23DM1 | 49 | 9691 | 1507 | 2 | 3 | 1 | 1 | 1 |
| LH252 | *L.hyperborea* | Bergen, Hordaland | 26.10.2022 | 5,66 | 13JAN23DM1 | 50 | 13567 | 1574 | 58 | 3 | 1 | 1 | 1 |
| LH251 | *L.hyperborea* | Bergen, Hordaland | 26.10.2022 | 6,06 | 13JAN23DM1 | 51 | 13169 | 832 | 3 | 3 | 1 | 1 | 1 |
| LH249 | *L.hyperborea* | Bergen, Hordaland | 26.10.2022 | 6,82 | 13JAN23DM1 | 52 | 15446 | 2752 | 0 | 3 | 1 | 1 | 1 |
| LH240 | *L.hyperborea* | Bergen, Hordaland | 15.06.2022 | 17,1 | 13JAN23DM1 | 53 | 6284 | 1754 | 0 | 2 | 0 | 1 | 1 |
| LH239 | *L.hyperborea* | Bergen, Hordaland | 15.06.2022 | 16 | 13JAN23DM1 | 54 | 11612 | 2578 | 0 | 2 | 0 | 1 | 1 |
| LH237 | *L.hyperborea* | Bergen, Hordaland | 15.06.2022 | 13,8 | 13JAN23DM1 | 55 | 9563 | 1886 | 0 | 2 | 0 | 1 | 1 |
| LH225 | *L.hyperborea* | Bergen, Hordaland | 15.06.2022 | 18 | 13JAN23DM1 | 56 | 17469 | 4256 | 46 | 2 | 0 | 1 | 1 |
| LH259 | *L.hyperborea* | Bergen, Hordaland | 26.10.2022 | 5,4 | 13JAN23DM1 | 57 | 4452 | 243 | 4 | 3 | 1 | 1 | 1 |
| LH258 | *L.hyperborea* | Bergen, Hordaland | 26.10.2022 | 6,72 | 13JAN23DM1 | 58 | 6481 | 504 | 6 | 3 | 1 | 1 | 1 |
| LH257 | *L.hyperborea* | Bergen, Hordaland | 26.10.2022 | 3,94 | 13JAN23DM1 | 59 | 7501 | 635 | 0 | 3 | 1 | 1 | 1 |
| LH256 | *L.hyperborea* | Bergen, Hordaland | 26.10.2022 | 11,6 | 13JAN23DM1 | 60 | 9705 | 983 | 2 | 3 | 1 | 1 | 1 |
| LH266 | *L.hyperborea* | Bergen, Hordaland | 26.10.2022 | 17,3 | 13JAN23DM1 | 63 | 17678 | 2503 | 22 | 3 | 1 | 1 | 1 |
| LH264 | *L.hyperborea* | Bergen, Hordaland | 26.10.2022 | 6,46 | 13JAN23DM1 | 64 | 9824 | 2732 | 14 | 3 | 1 | 1 | 1 |
| LH163 | *L.hyperborea* | Bergen, Hordaland | 03.08.2022 | 45,8 | 18JAN23DM1 | 17 | 8904 | 67 | 0 | 3 | 1 | 1 | 1 |
| LH164 | *L.hyperborea* | Bergen, Hordaland | 03.08.2022 | 68,6 | 18JAN23DM1 | 18 | 4245 | 2 | 0 | 2 | 1 | 0 | 1 |

| | | | | | | | | | | | | | |
|---|---|---|---|---|---|---|---|---|---|---|---|---|---|
| SL330 | *S. Latissima* | Bergen, Hordaland | 15.06.2022 | 12,8 | 18JAN23DM1 | 34 | 15957 | 275 | 4 | 2 | 1 | 0 | 1 |
| SL331 | *S. Latissima* | Bergen, Hordaland | 15.06.2022 | 16,3 | 18JAN23DM1 | 35 | 22350 | 7283 | 73 | 3 | 1 | 1 | 1 |
| SL333 | *S. Latissima* | Bergen, Hordaland | 15.06.2022 | 15,8 | 18JAN23DM1 | 36 | 13218 | 3697 | 3 | 3 | 1 | 1 | 1 |
| SL334 | *S. Latissima* | Bergen, Hordaland | 15.06.2022 | 24,4 | 18JAN23DM1 | 37 | 14575 | 189 | 4 | 3 | 1 | 1 | 1 |
| SL336 | *S. Latissima* | Bergen, Hordaland | 15.06.2022 | 22,2 | 18JAN23DM1 | 38 | 12466 | 7305 | 0 | 3 | 1 | 1 | 1 |
| SL339 | *S. Latissima* | Bergen, Hordaland | 15.06.2022 | 22,8 | 18JAN23DM1 | 39 | 4423 | 2941 | 36 | 2 | 0 | 1 | 1 |
| SL340 | *S. Latissima* | Bergen, Hordaland | 15.06.2022 | 23,4 | 18JAN23DM1 | 40 | 8842 | 148 | 0 | 2 | 0 | 1 | 1 |
| SL344 | *S. Latissima* | Bergen, Hordaland | 15.06.2022 | 15,3 | 18JAN23DM1 | 41 | 9598 | 774 | 4 | 3 | 1 | 1 | 1 |
| SL347 | *S. Latissima* | Bergen, Hordaland | 15.06.2022 | 26 | 18JAN23DM1 | 42 | 5535 | 122 | 0 | 2 | 0 | 1 | 1 |
| SL350 | *S. Latissima* | Bergen, Hordaland | 15.06.2022 | 8,88 | 18JAN23DM1 | 43 | 11840 | 5 | 0 | 2 | 1 | 0 | 1 |
| SL353 | *S. Latissima* | Bergen, Hordaland | 26.10.2022 | 6,96 | 18JAN23DM1 | 44 | 7476 | 764 | 12 | 2 | 0 | 1 | 1 |
| SL354 | *S. Latissima* | Bergen, Hordaland | 26.10.2022 | 8,3 | 18JAN23DM1 | 45 | 5990 | 2 | 2 | 2 | 1 | 0 | 1 |
| SL356 | *S. Latissima* | Bergen, Hordaland | 26.10.2022 | 14,4 | 18JAN23DM1 | 46 | 12992 | 540 | 18 | 3 | 1 | 1 | 1 |
| SL360 | *S. Latissima* | Bergen, Hordaland | 26.10.2022 | 7,7 | 18JAN23DM1 | 47 | 10954 | 2 | 62 | 2 | 1 | 0 | 1 |
| SL361 | *S. Latissima* | Bergen, Hordaland | 26.10.2022 | 21,2 | 18JAN23DM1 | 48 | 1201 | 0 | 0 | | | | |
| SL364 | *S. Latissima* | Bergen, Hordaland | 26.10.2022 | 9,68 | 18JAN23DM1 | 49 | 307 | 32 | 5 | 2 | 1 | 0 | 1 |
| SL365 | *S. Latissima* | Bergen, Hordaland | 26.10.2022 | 12,9 | 18JAN23DM1 | 50 | 7001 | 4 | 0 | 2 | 1 | 0 | 1 |
| SL368 | *S. Latissima* | Bergen, Hordaland | 26.10.2022 | 9,76 | 18JAN23DM1 | 51 | 10693 | 6 | 5 | 2 | 1 | 0 | 1 |
| SL370 | *S. Latissima* | Bergen, Hordaland | 26.10.2022 | 13,4 | 18JAN23DM1 | 52 | 1253 | 391 | 0 | 2 | 0 | 1 | 1 |
| SL371 | *S. Latissima* | Bergen, Hordaland | 26.10.2022 | 12,3 | 18JAN23DM1 | 53 | 5326 | 172 | 4 | 3 | 1 | 1 | 1 |
| LH66 | *L.hyperborea* | Bona Sea Rogaland | 27.07.2021 | 10,4 | 11OCT22-1 | 65 | 27661 | 5321 | 3 | 2 | 0 | 1 | 1 |
| LH69 | *L.hyperborea* | Bona Sea Rogaland | 27.07.2021 | 11,9 | 13JAN23DM1 | 1 | 10947 | 2 | 86 | 1 | 0 | 0 | 1 |
| LH67 | *L.hyperborea* | Bona Sea Rogaland | 27.07.2021 | 11,6 | 13JAN23DM1 | 2 | 6685 | 4 | 0 | | | | |
| LH60 | *L.hyperborea* | Bona Sea Rogaland | 27.07.2021 | 15,2 | 13JAN23DM1 | 3 | 6573 | 88 | 2 | 3 | 1 | 1 | 1 |
| LH56 | *L.hyperborea* | Bona Sea Rogaland | 27.07.2021 | 10,4 | 13JAN23DM1 | 4 | 5075 | 3 | 0 | | | | |
| LH55 | *L.hyperborea* | Bona Sea Rogaland | 27.07.2021 | 19,7 | 13JAN23DM1 | 5 | 8096 | 2 | 0 | 1 | 1 | 0 | 0 |
| LH75 | *L.hyperborea* | Bona Sea Rogaland | 27.07.2021 | 10,9 | 13JAN23DM1 | 9 | 4498 | 2 | 0 | 2 | 1 | 0 | 1 |
| LH74 | *L.hyperborea* | Bona Sea Rogaland | 27.07.2021 | 10,1 | 13JAN23DM1 | 10 | 3346 | 3 | 0 | 1 | 0 | 1 | 0 |
| LH72 | *L.hyperborea* | Bona Sea Rogaland | 27.07.2021 | 14,5 | 13JAN23DM1 | 11 | 6764 | 2 | 2 | | | | |
| LH70 | *L.hyperborea* | Bona Sea Rogaland | 27.07.2021 | 10,2 | 13JAN23DM1 | 12 | 10461 | 3 | 0 | 2 | 1 | 0 | 1 |
| K2 | *S.latissima* | Bud, Jøssingfjor, Vest-Agder | 25.07.2016 | 7,8 | 02NOV22_1 | 1 | 63 | 0 | 0 | | | | |
| K3 | *S.latissima* | Bud, Jøssingfjor, Vest-Agder | 25.07.2016 | 12,5 | 02NOV22_1 | 2 | 5809 | 1500 | 22 | 3 | 1 | 1 | 1 |
| K4 | *S.latissima* | Bud, Jøssingfjor, Vest-Agder | 25.07.2016 | 7,3 | 02NOV22_1 | 3 | 1492 | 543 | 0 | 2 | 0 | 1 | 1 |

| | | | | | | | | | | | | | |
|---|---|---|---|---|---|---|---|---|---|---|---|---|---|
| SL257 | *S. Latissima* | Båthavn v/Karmsund Bro | 25.03.2022 | 33,8 | 18JAN23DM1 | 24 | 30764 | 2102 | 3 | 2 | 0 | 1 | 1 |
| SL263 | *S. Latissima* | Båthavn v/Karmsund Bro | 25.03.2022 | 45 | 18JAN23DM1 | 25 | 9351 | 436 | 5 | 2 | 0 | 1 | 1 |
| SL272 | *S. Latissima* | Båthavn v/Karmsund Bro | 25.03.2022 | 38,8 | 18JAN23DM1 | 26 | 16572 | 1786 | 13 | 3 | 1 | 1 | 1 |
| SL283 | *S. Latissima* | Båthavn v/Karmsund Bro | 25.03.2022 | 33,2 | 18JAN23DM1 | 27 | 5098 | 1121 | 0 | 2 | 0 | 1 | 1 |
| K75 | *S.latissima* | Frøya, Sør-Trøndelag | 26.01.2017 | 8,6 | 02NOV22_1 | 17 | 1541 | 0 | 0 | 1 | 1 | 0 | 0 |
| K77 | *S.latissima* | Frøya, Sør-Trøndelag | 26.01.2017 | 16 | 02NOV22_1 | 18 | 472 | 0 | 0 | 2 | 1 | 0 | 1 |
| K79 | *S.latissima* | Frøya, Sør-Trøndelag | 26.01.2017 | 17,1 | 02NOV22_1 | 19 | 490 | 0 | 0 | 2 | 1 | 0 | 1 |
| K183 | *S.latissima* | Hardangerfjord | 08.08.2017 | 9,96 | 02NOV22_1 | 33 | 50 | 0 | 0 | | | | |
| K184 | *S.latissima* | Hardangerfjord | 08.08.2017 | 17,5 | 02NOV22_1 | 34 | 451 | 0 | 0 | | | | |
| K186 | *S.latissima* | Hardangerfjord | 08.08.2017 | 9,48 | 02NOV22_1 | 35 | 851 | 2 | 0 | 1 | 1 | 0 | 0 |
| K190 | *S.latissima* | Hardangerfjord | 08.08.2017 | 7,76 | 02NOV22_1 | 38 | 3711 | 243 | 0 | 2 | 0 | 1 | 1 |
| K191 | *S.latissima* | Hardangerfjord | 08.08.2017 | 5,84 | 02NOV22_1 | 39 | 1006 | 91 | 0 | 2 | 0 | 1 | 1 |
| K188 | *S.latissima* | Hardangerfjord | 08.08.2017 | 17,7 | 13JAN23DM1 | 65 | 226 | 0 | 0 | 2 | 1 | 0 | 1 |
| SL135 | *S. Latissima* | Haugesund, Rogaland | 02.11.2022 | 83,6 | 18JAN23DM1 | 12 | 10589 | 361 | 0 | 2 | 0 | 1 | 1 |
| SL139 | *S. Latissima* | Haugesund, Rogaland | 02.11.2022 | 102 | 18JAN23DM1 | 13 | 8790 | 2895 | 0 | 2 | 0 | 1 | 1 |
| SL149 | *S. Latissima* | Haugesund, Rogaland | 02.11.2022 | 108 | 18JAN23DM1 | 14 | 4001 | 885 | 0 | 3 | 1 | 1 | 1 |
| SL151 | *S. Latissima* | Haugesund, Rogaland | 02.11.2022 | 100 | 18JAN23DM1 | 15 | 14333 | 271 | 0 | 2 | 0 | 1 | 1 |
| SL153 | *S. Latissima* | Haugesund, Rogaland | 02.11.2022 | 76,8 | 18JAN23DM1 | 16 | 4903 | 641 | 7 | 3 | 1 | 1 | 1 |
| SL248 | *S. Latissima* | Haugesund, Rogaland | 02.11.2022 | 118 | 18JAN23DM1 | 23 | 10804 | 2304 | 0 | 3 | 1 | 1 | 1 |
| K7 | *S.latissima* | Kalak, Lebesby, Finnmark | 18.12.2016 | 5 | 02NOV22_1 | 4 | 3501 | 1464 | 44 | 2 | 0 | 1 | 1 |
| SL182 | *S. Latissima* | Karmøy, Rogaland | 02.11.2022 | 72,6 | 18JAN23DM1 | 19 | 1801 | 51 | 2 | 3 | 1 | 1 | 1 |
| SL185 | *S. Latissima* | Karmøy, Rogaland | 02.11.2022 | 106 | 18JAN23DM1 | 20 | 9720 | 360 | 0 | 2 | 0 | 1 | 1 |
| SL196 | *S. Latissima* | Karmøy, Rogaland | 02.11.2022 | 73 | 18JAN23DM1 | 22 | 5022 | 913 | 0 | 3 | 1 | 1 | 1 |
| K82 | *S.latissima* | Korsfjorden, Hordaland | 30.03.2017 | 16,1 | 02NOV22_1 | 20 | 1430 | 178 | 0 | 3 | 1 | 1 | 1 |
| K85 | *S.latissima* | Korsfjorden, Hordaland | 30.03.2017 | 21,2 | 02NOV22_1 | 22 | 178 | 3 | 0 | 1 | 0 | 1 | 0 |
| K87 | *S.latissima* | Korsfjorden, Hordaland | 30.03.2017 | 17,5 | 02NOV22_1 | 23 | 745 | 2 | 0 | 2 | 1 | 0 | 1 |
| K88 | *S.latissima* | Korsfjorden, Hordaland | 30.03.2017 | 11,7 | 02NOV22_1 | 24 | 1401 | 0 | 0 | 2 | 1 | 0 | 1 |
| K90 | *S.latissima* | Korsfjorden, Hordaland | 30.03.2017 | 14 | 02NOV22_1 | 25 | 349 | 19 | 0 | 2 | 0 | 1 | 1 |
| K91 | *S.latissima* | Korsfjorden, Hordaland | 30.03.2017 | 11 | 02NOV22_1 | 26 | 161 | 2 | 0 | 1 | 0 | 0 | 1 |
| K92 | *S.latissima* | Korsfjorden, Hordaland | 30.03.2017 | 12,4 | 02NOV22_1 | 27 | 1780 | 448 | 0 | 2 | 0 | 1 | 1 |
| K95 | *S.latissima* | Korsfjorden, Hordaland | 30.03.2017 | 4,4 | 02NOV22_1 | 28 | 1578 | 305 | 0 | 2 | 0 | 1 | 1 |
| K78 | *S.latissima* | Korsfjorden, Hordaland | 30.03.2017 | 8,6 | 02NOV22_1 | 29 | 256 | 0 | 0 | | | | |
| K50 | *S.latissima* | Krøttøy, Troms | 27.07.2016 | 24 | 02NOV22_1 | 14 | 1862 | 130 | 2 | 3 | 1 | 1 | 1 |

| | | | | | | | | | | | | | |
|---|---|---|---|---|---|---|---|---|---|---|---|---|---|
| SL190 | *S. Latissima* | Kvalsvik, Haugesund, Rogaland | 02.11.2022 | 76,2 | 18JAN23DM1 | 21 | 638 | 41 | 0 | 2 | 0 | 1 | 1 |
| K19 | *S.latissima* | Kvam, Hardangerfjord | 15.02.2016 | 7 | 02NOV22_1 | 21 | 2004 | 167 | 14 | 3 | 1 | 1 | 1 |
| K21 | *S.latissima* | Kvam, Hardangerfjord | 15.02.2016 | 5,6 | 13JAN23DM1 | 61 | 358 | 3 | 0 | | | | |
| K20 | *S.latissima* | Kvam, Hardangerfjord | 15.02.2016 | 9 | 13JAN23DM1 | 62 | 225 | 4 | 0 | | | | |
| K22 | *S.latissima* | Kvam, Hardangerfjord | 15.02.2016 | 6,7 | 13JAN23DM1 | 68 | 5543 | 945 | 2 | 2 | 1 | 0 | 1 |
| LH101 | *L.hyperborea* | Møre & Romsdal | 27.07.2021 | 14,5 | 13JAN23DM1 | 13 | 11914 | 400 | 0 | 3 | 1 | 1 | 1 |
| LH100 | *L.hyperborea* | Møre & Romsdal | 27.07.2021 | 13 | 13JAN23DM1 | 14 | 5195 | 23 | 0 | 2 | 1 | 0 | 1 |
| LH95 | *L.hyperborea* | Møre & Romsdal | 27.07.2021 | 11 | 13JAN23DM1 | 15 | 8485 | 2169 | 0 | 3 | 1 | 1 | 1 |
| LH78 | *L.hyperborea* | Møre & Romsdal | 27.07.2021 | 11,3 | 13JAN23DM1 | 16 | 20624 | 7994 | 4 | 3 | 1 | 1 | 1 |
| LH116 | *L.hyperborea* | Møre & Romsdal | 27.07.2021 | 12,2 | 13JAN23DM1 | 19 | 21770 | 8025 | 41 | 2 | 0 | 1 | 1 |
| LH112 | *L.hyperborea* | Møre & Romsdal | 27.07.2021 | 15,2 | 13JAN23DM1 | 20 | 11473 | 837 | 3 | 3 | 1 | 1 | 1 |
| LH108 | *L.hyperborea* | Møre & Romsdal | 27.07.2021 | 12,7 | 13JAN23DM1 | 21 | 9879 | 1988 | 11 | 3 | 1 | 1 | 1 |
| LH107 | *L.hyperborea* | Møre & Romsdal | 27.07.2021 | 25 | 13JAN23DM1 | 22 | 14369 | 3946 | 16 | 2 | 0 | 1 | 1 |
| LH104 | *L.hyperborea* | Møre & Romsdal | 27.07.2021 | 15,5 | 13JAN23DM1 | 23 | 9457 | 3 | 0 | 1 | 1 | 0 | 0 |
| LH103 | *L.hyperborea* | Møre & Romsdal | 27.07.2021 | 17 | 13JAN23DM1 | 24 | 6568 | 58 | 87 | 3 | 1 | 1 | 1 |
| K28 | *S.latissima* | Porsanger, Finmark | 08.03.2016 | 4,5 | 02NOV22_1 | 10 | 1876 | 551 | 16 | 2 | 0 | 1 | 1 |
| K30 | *S.latissima* | Porsanger, Finmark | 08.03.2016 | 5,6 | 02NOV22_1 | 11 | 2069 | 40 | 2 | 3 | 1 | 1 | 1 |
| K145 | *S.latissima* | Sognefjord | 27.04.2017 | 15,3 | 02NOV22_1 | 30 | 2207 | 497 | 0 | 3 | 1 | 1 | 1 |
| K108 | *S.latissima* | Sognefjord | 25.04.2017 | 20 | 02NOV22_1 | 31 | 2345 | 32 | 2 | 3 | 1 | 1 | 1 |
| K170 | *S.latissima* | Sognefjord | 28.04.2017 | 20 | 02NOV22_1 | 32 | 2485 | 2 | 2 | 1 | 1 | 0 | 0 |
| K178 | *S.latissima* | Sognefjord | 28.04.2017 | 15,5 | 02NOV22_1 | 36 | 1478 | 0 | 0 | 2 | 1 | 0 | 1 |
| K179 | *S.latissima* | Sognefjord | 28.04.2017 | 20,8 | 02NOV22_1 | 37 | 58 | 0 | 0 | | | | |
| K172 | *S.latissima* | Sognefjord | 28.04.2017 | 23,2 | 02NOV22_1 | 40 | 2165 | 48 | 0 | 2 | 0 | 1 | 1 |
| K173 | *S.latissima* | Sognefjord | 28.04.2017 | 18,6 | 02NOV22_1 | 41 | 2282 | 90 | 0 | 2 | 0 | 1 | 1 |
| K175 | *S.latissima* | Sognefjord | 28.04.2017 | 18,6 | 02NOV22_1 | 42 | 677 | 130 | 0 | 2 | 0 | 1 | 1 |
| K180 | *S.latissima* | Sognefjord | 28.04.2017 | 19,3 | 13JAN23DM1 | 66 | 3334 | 0 | 2 | 3 | 1 | 1 | 1 |
| K166 | *S.latissima* | Sognefjord | ? | 20 | 13JAN23DM1 | 67 | 6916 | 54 | 0 | 3 | 1 | 1 | 1 |
| K41 | *S.latissima* | Sommarøy, Troms | 08.12.2016 | 6,1 | 02NOV22_1 | 12 | 51 | 0 | 0 | | | | |
| K44 | *S.latissima* | Sommarøy, Troms | 08.12.2016 | 13,2 | 02NOV22_1 | 13 | 204 | 20 | 0 | 2 | 0 | 1 | 1 |
| SL291 | *S. Latissima* | Størøy, Haugesund | 25.03.2022 | 36,4 | 18JAN23DM1 | 28 | 18601 | 4644 | 0 | 3 | 1 | 1 | 1 |
| SL295 | *S. Latissima* | Størøy, Haugesund | 25.03.2022 | 88,2 | 18JAN23DM1 | 29 | 2375 | 127 | 0 | 2 | 0 | 1 | 1 |
| SL306 | *S. Latissima* | Størøy, Haugesund | 25.03.2022 | 43,6 | 18JAN23DM1 | 30 | 21457 | 1185 | 0 | 3 | 1 | 1 | 1 |
| SL307 | *S. Latissima* | Størøy, Haugesund | 25.03.2022 | 32,2 | 18JAN23DM1 | 31 | 17985 | 4600 | 11 | 3 | 1 | 1 | 1 |

| | | | | | | | | | | | | | | |
|---|---|---|---|---|---|---|---|---|---|---|---|---|---|---|
| SL310 | *S. Latissima* | Størøy, Haugesund | 25.03.2022 | 51,6 | 18JAN23DM1 | 32 | 18128 | 3507 | 19 | 3 | 1 | 1 | 1 |
| SL327 | *S. Latissima* | Størøy, Haugesund | 25.03.2022 | 33,4 | 18JAN23DM1 | 33 | 15070 | 1027 | 5 | 3 | 1 | 1 | 1 |
| K10 | *S.latissima* | Søgne, Vest-Agder | 13.12.2016 | 9,7 | 02NOV22_1 | 5 | 3534 | 1077 | 50 | 2 | 0 | 1 | 1 |
| K11 | *S.latissima* | Søgne, Vest-Agder | 13.12.2016 | 6,7 | 02NOV22_1 | 6 | 5617 | 1383 | 70 | 3 | 1 | 1 | 1 |
| K12 | *S.latissima* | Søgne, Vest-Agder | 13.12.2016 | 6,3 | 02NOV22_1 | 7 | 3088 | 916 | 35 | 3 | 1 | 1 | 1 |
| K15 | *S.latissima* | Søgne, Vest-Agder | 13.12.2016 | 13 | 02NOV22_1 | 8 | 1632 | 813 | 0 | 2 | 0 | 1 | 1 |
| K16 | *S.latissima* | Søgne, Vest-Agder | 13.12.2016 | 10,5 | 02NOV22_1 | 9 | 3247 | 125 | 111 | 2 | 1 | 0 | 1 |
| SL17 | *S.latissima* | Trollsøy, Austevoll | 05.04.2021 | 20,6 | 02NOV22_1 | 43 | 1276 | 117 | 0 | 2 | 0 | 1 | 1 |
| SL26 | *S.latissima* | Trollsøy, Austevoll | 05.04.2021 | 5,58 | 02NOV22_1 | 44 | 2854 | 53 | 0 | 3 | 1 | 1 | 1 |
| SL29 | *S.latissima* | Trollsøy, Austevoll | 05.04.2021 | 6,62 | 02NOV22_1 | 45 | 2700 | 30 | 0 | 3 | 1 | 1 | 1 |
| SL30 | *S.latissima* | Trollsøy, Austevoll | 05.04.2021 | 6,56 | 02NOV22_1 | 46 | 3310 | 50 | 0 | 2 | 1 | 1 | 0 |
| SL32 | *S.latissima* | Trollsøy, Austevoll | 05.04.2021 | 4,98 | 11OCT22-1 | 73 | 41632 | 365 | 3 | 3 | 1 | 1 | 1 |
| SL33 | *S.latissima* | Trollsøy, Austevoll | 05.04.2021 | 6,66 | 11OCT22-1 | 74 | 30471 | 362 | 3 | 3 | 1 | 1 | 1 |
| SL34 | *S.latissima* | Trollsøy, Austevoll | 05.04.2021 | 7,32 | 11OCT22-1 | 75 | 14656 | 125 | 3 | 3 | 1 | 1 | 1 |
| SL35 | *S.latissima* | Trollsøy, Austevoll | 05.04.2021 | 3,66 | 11OCT22-1 | 76 | 1228 | 0 | 0 | 2 | 1 | 0 | 1 |
| SL49 | *S.latissima* | Trollsøy, Austevoll | 05.04.2021 | 7,38 | 11OCT22-1 | 77 | 17721 | 145 | 0 | 3 | 1 | 1 | 1 |
| SL54 | *S.latissima* | Trollsøy, Austevoll | 05.04.2021 | 12,6 | 11OCT22-1 | 78 | 15259 | 203 | 5 | 3 | 1 | 1 | 1 |
| K59 | *S.latissima* | Vengsøya, Troms | 01.09.2017 | 4,4 | 02NOV22_1 | 15 | 1605 | 471 | 5 | 2 | 0 | 1 | 1 |
| K63 | *S.latissima* | Vengsøya, Troms | 01.09.2017 | 2,2 | 02NOV22_1 | 16 | 2254 | 484 | 0 | 2 | 0 | 1 | 1 |
| LH23 | *L.hyperborea* | Vikna, Nord-Trondelag | 16.06.2021 | 14,4 | 11OCT22-1 | 66 | 14235 | 3 | 7 | 2 | 1 | 0 | 1 |
| LH28 | *L.hyperborea* | Vikna, Nord-Trondelag | 16.06.2021 | 14,4 | 11OCT22-1 | 67 | 9839 | 0 | 2 | 1 | 0 | 1 | 0 |
| LH29 | *L.hyperborea* | Vikna, Nord-Trondelag | 16.06.2021 | 14,3 | 11OCT22-1 | 68 | 3893 | 991 | 0 | 2 | 0 | 1 | 1 |
| LH36 | *L.hyperborea* | Vikna, Nord-Trondelag | 16.06.2021 | 15,6 | 11OCT22-1 | 69 | 26837 | 6145 | 86 | 3 | 1 | 1 | 1 |
| LH17 | *L.hyperborea* | Vikna, Nord-Trondelag | 16.06.2021 | 15,2 | 11OCT22-1 | 70 | 58514 | 15825 | 333 | 2 | 0 | 1 | 1 |
| LH14 | *L.hyperborea* | Vikna, Nord-Trondelag | 16.06.2021 | 14,5 | 11OCT22-1 | 79 | 39044 | 8138 | 16 | 3 | 1 | 1 | 1 |
| LH15 | *L.hyperborea* | Vikna, Nord-Trondelag | 16.06.2021 | 17,1 | 11OCT22-1 | 80 | 15437 | 962 | 0 | 2 | 0 | 1 | 1 |
| LH33 | *L.hyperborea* | Vikna, Nord-Trondelag | 16.06.2021 | 14,1 | 13JAN23DM1 | 6 | 1745 | 3 | 0 | 1 | 0 | 1 | 0 |
| LH22 | *L.hyperborea* | Vikna, Nord-Trondelag | 16.06.2021 | 16,3 | 13JAN23DM1 | 7 | 10096 | 2409 | 2 | 3 | 1 | 1 | 1 |
| LH20 | *L.hyperborea* | Vikna, Nord-Trondelag | 16.06.2021 | 16,8 | 13JAN23DM1 | 8 | 7113 | 358 | 5 | 3 | 1 | 1 | 1 |

**Table S4: GenBank accession numbers.**

| Name | Genbank Accession |
|---|---|
| Acanthamoeba polyphaga mimivirus | YP_003986929 |
| Acanthamoeba turfacea chlorella virus 1 | ABT16414 |
| Acanthamoeba turfacea chlorella virus MN0810 | AGE55620 |
| Cafeteria roenbergensis virus | ADO67376 |
| Chrysochromulina ericina virus CeV-01B | ABU23712 |
| Ecklonia maxima DH-ZA-D1735 | MG967362 |
| Ecklonia maxima PN-ZA-D1764 | MG967368 |
| Ecklonia maxima PN-ZA-D1765 | MG967369 |
| Ecklonia maxima PN-ZA-D1766 | MG967370 |
| Ecklonia radiata EradKM-ZA-D1760 | MG967367 |
| Ecklonia radiata EradKM-ZA-D1763 | MG967366 |
| Ectocarpus fasciculatus Efas1 | HG003334 |
| Ectocarpus fasciculatus Efas2 | HG003335 |
| Ectocarpus siliculosus EsV1 | AAK14534 |
| Feldmannia irregularis FirrV-1 | AAR26925 |
| Feldmannia simplex Flex1 | HG003340 |
| Feldmannia simplex Flex2 | HG003341 |
| Feldmannia simplex Flex3 | HG003342 |
| Feldmannia simplex Flex8 | HG003337 |
| Feldmannia species virus 158 | YP_002154681 |
| Fowlpox virus | AAZ14082 |
| Heterosigma akashiwo virus 01 | AOM63514 |
| LhypPH10-3 isolate | KP296733 |
| Macrocystis pyrifera MpyrPN-CL-3 | MG967376 |
| Megavirus chilensis | YP_004894515 |
| Megavirus courdo11 | AFX92519 |
| Micromonas pusilla virus SP1 | AET84889 |
| Moumouvirus goulette | AGF85360 |
| Myriotrichia clavaeformis 2 | HG003343 |
| Organic lake phycodnavirus 1 | ADX05938 |
| Organic lake phycodnavirus 2 | ADX06358 |
| Ostreococcus tauri virus 1 | YP_003212988 |
| Ostreococcus tauri virus 2 | YP_004063587 |
| Ostreococcus tauri virus 5 | YP_001648266 |
| Paramecium bursaria Chlorella virus 1 | AAA88828 |
| Paramecium bursaria Chlorella virus AR158 | ABU44077 |
| Paramecium bursaria Chlorella virus CVK2 | BAA35143 |
| Paramecium bursaria Chlorella virus NY2A | ABT14984 |
| Phaeocystis globosa virus PgV | AET73005 |
| Phaeocystis pouchetii PpV-01 | ABU23715 |

| | |
|---|---|
| Pylaiella littoralis 1 | HG003336 |
| Pyramimonas orientalis PoV-01B | ABU23714 |
| SlatPH10-7 isolate | KP296731 |
| SlatPH10-7.2 isolate | KP296734 |
| Undaria pinnatifida IC-SK-1 | MG967371 |
| Undaria pinnatifida IC-SK-2 | MG967372 |
| Undaria pinnatifida IC-SK-3 | MG967373 |
| Undaria pinnatifida IC-SK-4 | MG967374 |
| Undaria pinnatifida IC-SK-5 | MG967375 |
| Yellowstone lake phycodnavirus 1 | YP_009174754 |
| Yellowstone lake phycodnavirus 2 | YP_009174565 |
| Yellowstone lake phycodnavirus 3 | YP_009174294 |

**Table S5: Number of viral positive (+) and negative (-) samples for each kelp species and year, and in total.**

| | ***S.latissima*** | **+** | **-** | ***L.hyperborea*** | **+** | **-** |
|---|---|---|---|---|---|---|
| 2021-2022 | 372 | 350 | 21 | 270 | 263 | 7 |
| 2016-2018 | 157 | 96 | 61 | | | |
| TOTAL | 529 | 446 | 82 | 270 | 263 | 7 |

**Table S6: Number of infected, non-infected and total number of samples of *S.latissima* from the different areas (defined in Table 1) from 2016-2018.**

| | **South** | **North** | **Fjords** |
|---|---|---|---|
| Infected | 57 | 15 | 46 |
| Non infected | 22 | 27 | 20 |
| Total num. samples | 79 | 42 | 66 |
